# Draft genome of the protandrous Chinese black porgy, *Acanthopagrus schlegelii*

**DOI:** 10.1093/gigascience/giy012

**Published:** 2018-02-26

**Authors:** Zhiyong Zhang, Kai Zhang, Shuyin Chen, Zhiwei Zhang, Jinyong Zhang, Xinxin You, Chao Bian, Jin Xu, Chaofeng Jia, Jun Qiang, Fei Zhu, Hongxia Li, Hailin Liu, Dehua Shen, Zhonghong Ren, Jieming Chen, Jia Li, Tianheng Gao, Ruobo Gu, Junmin Xu, Qiong Shi, Pao Xu

**Affiliations:** 1 Jiangsu Marine Fishery Research Institute, Nantong, Jiangsu 226007, China; 2 Freshwater Fishery Research Center, Chinese Academy of Fishery Sciences, Wuxi, Jiangsu 214081, China; 3 Shenzhen Key Lab of Marine Genomics, Guangdong Provincial Key Lab of Molecular Breeding in Marine Economic Animals, BGI Academy of Marine Sciences, BGI Marine, BGI, Shenzhen 518083, China; 4 BGI Education Center, University of Chinese Academy of Sciences, Shenzhen, Guangdong 518083, China; 5 State Key Laboratory of Freshwater Ecology and Biotechnology, Institute of Hydrobiology, Chinese Academy of Sciences, Wuhan, Hubei 430000, China; 6 BGI-Zhenjiang Institute of Hydrobiology, Zhenjiang, Jiangsu 212000, China; 7 College of Oceanography, Hohhai University, Nanjing, Jiangsu 210098, China

**Keywords:** Chinese black porgy, *Acanthopagrus schlegelii*, whole genome sequencing, genome assembly, sex change–related genes

## Abstract

**Background:**

As one of the most popular and valuable commercial marine fishes in China and East Asian countries, the Chinese black porgy (*Acanthopagrus schlegelii*), also known as the blackhead seabream, has some attractive characteristics such as fast growth rate, good meat quality, resistance to diseases, and excellent adaptability to various environments. Furthermore, the black porgy is a good model for investigating sex changes in fish due to its protandrous hermaphroditism. Here, we obtained a high-quality genome assembly of this interesting teleost species and performed a genomic survey on potential genes associated with the sex-change phenomenon.

**Findings:**

We generated 175.4 gigabases (Gb) of clean sequence reads using a whole-genome shotgun sequencing strategy. The final genome assembly is approximately 688.1 megabases (Mb), accounting for 93% of the estimated genome size (739.6 Mb). The achieved scaffold N50 is 7.6 Mb, reaching a relatively high level among sequenced fish species. We identified 19 465 protein-coding genes, which had an average transcript length of 17.3 kb. By performing a comparative genomic analysis, we found 3 types of genes potentially associated with sex change, which are useful for studying the genetic basis of the protandrous hermaphroditism.

**Conclusions:**

We provide a draft genome assembly of the Chinese black porgy and discuss the potential genetic mechanisms of sex change. These data are also an important resource for studying the biology and for facilitating breeding of this economically important fish.

## Data Description

### Background information

As one of the most popular and valuable commercial marine fishes in China and East Asian countries, the Chinese black porgy (*Acanthopagrus schlegelii*), also known as the blackhead seabream, has some interesting characteristics such as fast growth rate, good meat quality, resistance to diseases, and good adaptability to various environments. It is often farmed for food in the South China Sea and the coastal waters around Japan and Korea [1,2]. In addition, it is an eurythermal and euryhaline fish, living in a wide range of water temperatures and salinities. Recently, some basic studies on the genetic improvement of its growth and its disease resistance have been performed in order to increase efficiency of farming [3].

The Chinese black porgy is also a good model for investigating the genetic mechanisms of sex change due to its interesting life cycle. It is a functional male during the first 2 years and subsequently a female during the next couple of years. Recently, a good hybrid of the Japanese seabream (*Pagrosomus major*; ♀) and the Chinese black porgy (♂) has become available [4,5], with better growth performance and higher tolerance against low temperature than its parents.

However, the genetic mechanisms for these interesting biological characteristics are still unclear. Here, we sequenced and assembled the whole genome of the Chinese black porgy, before performing a genomic survey on potential genes associated with the sex-change phenomenon.

### Sample and sequencing

**Figure 1:**
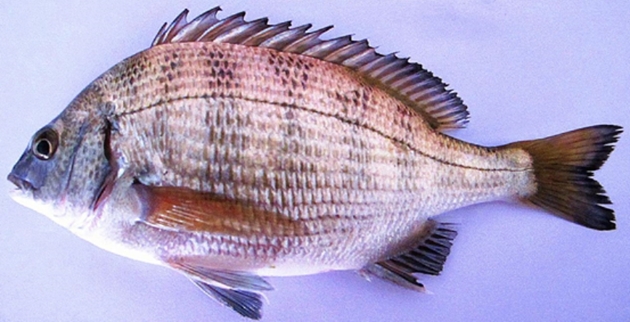
Image of a Chinese black porgy captured from Laizhou Bay in Yantai, Shandong Province, China.

The wild black porgy (National Center for Biotechnology Information [NCBI] Taxonomy ID: 72 011; Fishbase ID: 6531) individuals (Fig. 1) were collected from Laizhou Bay in Yantai, Shandong Province, China. Genomic DNA was extracted from the muscle of a female fish using Qiagen GenomicTip100 (Qiagen, Hilden, USA). We used the whole-genome shotgun sequencing strategy and constructed the subsequent 3 short-insert libraries (250-bp, 500-bp, and 800-bp) and 4 long-insert libraries (2-kb, 5-kb, 10-kb, and 20-kb) in accordance with the standard protocol from Illumina (San Diego, USA). These constructed libraries were sequenced on the Illumina HiSeq 2000 system [6] (the read length is 125 bp). Finally, we generated 272.9-Gb raw reads from all 7 libraries.

Before assembly of the sequencing reads, SOAPfilter v2.2 software [7] with default parameters (-y -p -g 1 -o clean -M 2 -f 0) was used to remove low-quality raw reads (including reads with 10 or more nonsequenced/low-quality bases), polymerase chain reaction duplicates, and adaptor sequences. Subsequently, we obtained approximately 175.4 Gb of clean reads for further genome size prediction and assembling. A *k*-mer analysis with the formula G = *k*_num/*k*_depth [8] was performed to estimate the genome size of Chinese black porgy. In our current study, the achieved total number of *k-*mers and *k*_depth was 2.81 × 10^10^ and 38, respectively. Therefore, the genome size of Chinese black porgy is estimated to be 739.6 Mb. Based on this result, the retained reads were calculated to cover approximately 238-fold of the whole genome.

### Assembly and evaluation

To obtain a genome assembly, we used SOAPdenovo2 v2.04.4 (SOAPdenovo2, RRID:SCR_014986) [9] with optimized parameters (pre-graph -K 27 -p 16 -d 1; contig –M 3; scaff -F -b 1.5 -p 16) to deal with these clean reads. In brief, the reads from short-insert libraries were applied for the contig assembly, before alignment of all the filtered reads onto the contigs for linking these contigs to generate scaffolds. GapCloser v1.12 (GapCloser, RRID:SCR_015026) [7] with default parameters was subsequently used to fill some intra-scaffold gaps in the local assembly, in which the reads were equipped with one end uniquely mapped to a contig and the other end located within a gap. Meanwhile, SSPACE (version 2.0) [10] with default parameters was used to obtain super scaffolds with the reads from the long-insert libraries (2-kb, 5-kb, 10-kb, and 20-kb). The final genome assembly was approximately 688.1 Mb, which accounts for 93.0% of the estimated genome size (739.6 Mb; Table 1).

**Table 1:** Summary of the genome assembly and annotation

Genome assembly parameter	
contig N50 size, kb	17.2
contig number, > 100 bp	115 091
Scaffold N50 size, Mb	7.6
Scaffold number, > 100 bp	31 359
Total length, Mb	688.1
Genome coverage, ×	257.6
Longest scaffold, bp	22 574 836
Genome annotation parameter	
Protein-coding gene number	19 465
Mean transcript length, kb	17.3
Mean exons per gene	11.1
Mean exon length, bp	170.2
Mean intron length, bp	1519.2

The achieved scaffold N50 is 7.64 Mb, reaching a relatively high length among sequenced fish species. In comparison, other scaffolds have levels of 1.55 Mb for the zebrafish [11], 1.1 Mb for platy fish [12], 867 kb for half-smooth tongue sole [13], 1 Mb for common carp [14], 6.4 Mb for grass carp [15], 2.97 Mb for Atlantic salmon [16], 1.8 Mb for a seahorse [17], and 1.15 Mb for a Chinese barbel fish [18]. The Core Eukaryotic Genes Mapping Approach (CEGMA, RRID:SCR_015055), version 2.5 [19], with a set of 248 conserved core eukaryotic genes (CEGs) was used to assess the completeness of the final assembly. The estimates suggest that 90.7% CEGs are complete and 92.3% are partial. Meanwhile, Benchmarking Universal Single-Copy Orthologs (BUSCO, RRID:SCR_015008), version 3, [20] was applied to evaluate the quality of the generated genome assembly. We chose the representative actinopterygian gene set with 4584 single-copy genes as the reference. The BUSCO values were calculated as follows: C: 89.1% [S: 86.2%, D: 2.9%], F: 2.5%, M: 8.4%, n: 4584, in which percentages of the total gene number (n) for the complete (C), single (S), duplicated (D), fragmented (F), and missed (M) are clarified. The results from CEGMA and BUSCO suggest that the assembled genome covers the majority of the gene space.

### Annotation

We used RepeatProteinMask (version 4.0.6) [21] in RepeatMasker (RepeatMasker, RRID:SCR_012954) to identify the repetitive sequences, before using RepeatModeller (version 1.05) [22] and LTR_FINDER.x86_64-1.0.6 to construct a *de novo* repeat library. Additionally, repetitive elements were predicted using Tandem Repeat Finder (version 4.04). Finally, we observed that the identified repeat sequences cover 19.78% of the assembled genome (Table 2).

**Table 2:** Detailed classification of repeat sequences in the assembled genome

	Repbase TEs		TE proteins		*De novo*		Combined TEs	
				
Type	Length, Mb	In genome, %	Length, Mb	In genome, %	Length, Mb	In genome, %	Length, Mb	In genome, %
DNA	20.930	3.041	2.200	0.320	58.340	8.479	68.130	9.902
LINE	10.240	1.488	6.950	1.010	26.760	3.889	33.020	4.789
SINE	1.120	0.163	2.340	0.000	3.780	0.550	4.550	0.661
LTR	7.200	1.046	35.410	0.340	25.980	3.062	31.270	4.544
Other	0.020	0.003	0.000	0.000	0.000	0.000	0.020	0.003
Unknown	0.000	0.000	0.000	0.000	25.370	3.687	25.370	3.687
Total	35.300	5.130	11.480	1.669	124.540	18.099	136.240	19.780

Prediction of protein-coding genes was performed based on the integration of *ab initio* prediction, homologue prediction, and transcriptome-based prediction. The *ab initio* prediction was carried out with Augustus (Augustus: Gene Prediction, RRID:SCR_008417), version 2.5 [23], and GENSCAN (GENSCAN, RRID:SCR_012902), version 1.0, [24], on the repeat-masked assembly. For the homology-based gene prediction, homologous proteins of several reported fishes (zebrafish, Japanese puffer, stickleback, and medaka) were downloaded from Ensembl release 75 and aligned to the assembled genome using tBlastn (version 2.2.19) with e-value ≤ 1e^–5^. Subsequently, all the achieved alignments were analyzed using Genewise (version 2.2.0) software [25] to search for precise gene structures. We further filtered out these short (less than 150 bp), prematurely terminated or frame-shifted genes. For the transcriptome-based prediction, we obtained transcriptome data from a mixture of liver, muscle, skin, gill, and brain of a female fish at cDNA level. Those with low-quality bases, adapter sequences, and duplicated sequences were removed, and we acquired approximately 8 Gb of high-quality clean reads. Subsequently, TopHat2.1.1 [26] and Cufflinks (Cufflinks, RRID:SCR_014597), version 2.2.1 [27], were applied to predict gene structures using these retained reads. Eventually, the 3 gene sets generated from the prediction approaches were integrated into a comprehensive and nonredundant gene set using GLEAN [28]. As summarized in Table 1, the final gene set contains 19 465 genes, with an average transcript length of 17.3 kb. In addition, we ran BUSCO v3 [20] on the predicted coding sequences (CDS), and the final BUSCO score was up to 85.5% (C:85.5% [S:82.3%, D:3.2%], F:2.8%, M:11.7%, n:4584).

Simultaneously, all the protein sequences from the GLEAN analysis were mapped onto several public databases, including Pfam [29], PRINTS [30], ProDom [31], and SMART [32], to detect the known motifs and domains within our genome assembly. The data demonstrated that 99.3% of the predicted genes from the assembled genome contain at least 1 related functional assignment from other public databases, including Swiss-Prot [33], Interpro [34], TrEMBL [35], and KEGG [36].

### Phylogenetic analysis

In order to examine the phylogenetic position of the Chinese black porgy, we downloaded protein sequences of 7 reported fishes, including spotted gar (*Lepisosteus oculatus*), stickleback (*Gasterosteus aculeatus*), Japanses fugu (*Takifugu rubripes*), medaka (*Oryzias latipes*), zebrafish (*Danio rerio*), platyfish (*Xiphophorus maculatus*), and Nile tilapia (*Oreochromis niloticus*) from Ensembl (release 83) [37]. These sequences were used to construct gene families by OrthoMCL (OrthoMCL DB: Ortholog Groups of Protein Sequences, RRID:SCR_007839) [38] and eventually generated 17 431 gene families by the all-to-all Basic Local Alignment Search Tool for Proteins strategy with an e-value of 1e^–5^. In additional, 65 gene families were only presented in the black porgy genome.

**Figure 2:**
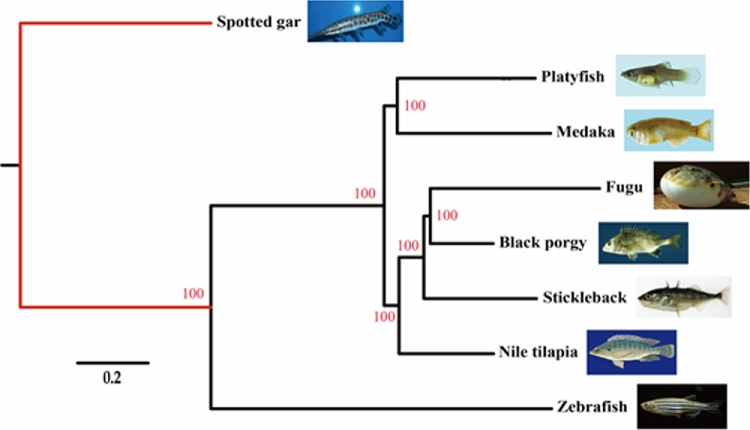
Phylogeny of ray-finned fishes. Spotted gar was used as the outgroup. The bootstrap support value for the topology is 100. The pictures in the phylogenetic tree were downloaded from Fishbase.

Subsequently, 3239 single-copy orthologous genes from these gene families were selected. These single-copy genes were further aligned using MUSCLE (MUSCLE, RRID:SCR_011812), version 3.8.31, with default parameters [39], before the protein alignments were changed to corresponding CDS using an in-house perl script. All nucleotide sequences of each species were integrated into a supergene, which was used to build a phylogenetic tree with PhyML (PhyML, RRID:SCR_014629) [40]. Our final data orientated the phylogenetic position of the black porgy in teleost (Fig. 2).

### Analysis of 3 types of genes for sex change

Sex change (secondary sex determination) is a universal phenomenon in fish but it usually does not occur in amphibians and mammals. The black porgy is a good model for the study on the molecular mechanisms of sex change. For providing a genomic survey on these genes in the assembled genome, protein sequences of 3 main types of genes potentially associated with sex change, including sex determination and differentiation genes, pluripotency factors, and apoptosis factors [41–43], were downloaded from the NCBI database and used for homology searches against the black porgy genome with tBlastn (version 2.2.19) [44]. We chose alignments with coverage >70% and identity >70% for further prediction of gene structures using Genewise (version 2.2.0) [25]. Finally, we obtained homologous sequences of 26 genes in the genome assembly of Chinese black porgy (for more details, see Table 3). All these predicted protein sequences were used to build a phylogenetic tree using PhyML [40], and we eventually observed that they were clustered with each corresponding homologue from other vertebrates.

**Table 3:** Three types of genes potentially related to sex change in the black porgy genome

Sex determination and differentiation genes			
	
Gene	Copy number	Scaffold	Copy number in other teleosts
*fst*	2	10, 17	1 (zebrafish, medaka)
*sox9*	5	11, 13, 16, 19, 27	2 (zebrafish, catfish)
*vasa*	10	11, 14, 16, 20, 27, 34, 37, 47, 53, 68	1 (zebrafish), 3 (Nile tilapia)
*ctnnb1*	4	2, 16, 64, 115	1 (zebrafish)
*piwil1*	1	15	1 (zebrafish, medaka)
*piwil2*	1	15	1 (zebrafish, medaka)
*sf1*	1	108	1 (zebrafish, medaka)
*rspo1*	2	2, 74	1 (zebrafish, medaka)
*foxl2*	2	1, 22	1 (zebrafish, medaka)
*cyp19a1a*	2	8, 28	1 (zebrafish, medaka)
*gsdf*	1	3	1 (medaka)
*figla*	1	32	1 (zebrafish, medaka)
*dmrt1*	1	56	1 (zebrafish, medaka, tongue sole)
*wnt4*	15	1, 2, 5, 6, 7, 8, 9, 18, 19, 20, 32, 34, 62, 67, 122	2 (zebrafish), 3 (rainbow trout)
*dax1*	4	2, 3, 14, 43	1 (medaka, tongue sole)
*cyp11a1*	2	8, 33	1 (zebrafish)
*hsd3b1*	2	7, 36	1 (zebrafish, medaka)
*amhr2*	2	9, 185	1 (medaka)
*jnk1*	9	1, 3, 4, 5, 16, 17, 38, 79, 117	1 (zebrafish)
Pluripotency factors			
*klf4*	5	1, 3, 17, 96, 142	1 (zebrafish, medaka)
*nr5a2*	3	8, 19, 28	1 (zebrafish, medaka)
*lin28a*	2	2, 3	1 (zebrafish)
*pou2*	1	3	1 (zebrafish)
Apoptosis factors			
*traf2*	2	3, 15	1 (zebrafish, medaka)
*casp2*	1	2	1 (zebrafish)
*tnfr1*	1	2	1 (zebrafish, medaka, tilapia)

Note: The last column states the gene copy number in other teleosts based on the phylogenetic trees (uploaded to GigaDB [69]) in this study.

Previous studies have revealed that multiple genes, including *dmrt1*, *cyp19a1a*, *wnt4*, *sox9*, *sf1*, *foxl2*, *figla*, *amhr2*, and *dax1*, are associated with sex change in the black porgy [41,45–47]. These sex determination and differentiation genes were also identified in our assembled scaffolds (in the first batch in Table 3). In the current study, the important male-related *dmrt1* and the steroidogenesis-suppressing factor *dax1* were mapped on scaffolds 56 and 14 of the black porgy genome, respectively.

It was reported that *dmrt1* may play a key role in the sex change of the black porgy, while the male-phase maintenance of male development was regulated by the brain–pituitary–gonadal axis via the GnRH-GtH-Dmrt1 pathway [41]. In the economically important half-smooth tongue sole (*Cynoglossus semilaevis*), *dmrt1* has been proven to be a necessary male sex-determining gene [48,49]. Moreover, previous findings suggest that a duplicate of *dmrt1* is the male sex determinant in medaka and *dmrt1* mutation causes a male–female sex reversal [50,51]. We also validated the existence of *foxl2* and *cyp19a1a*, 2 putative female-related genes, in the black porgy genome. Previous findings revealed that *cyp19a1a* plays dual roles in gonadal development, while both *cyp19a1a* and *foxl2* are related to the sex change of the black porgy [47]. However, *foxl2* has proved to participate in sex differentiation, although it is not essential for the sex determination and sex change in the tongue sole [52].

With only one copy in the black porgy, *figla* is a germ-cell–specific transcription factor related to ovary development and differentiation [53]. However, 2 isotypes (*figla_tv1* and *figla_tv2*) were reported in the tongue sole. It is noted that *figla_tv1* possesses a conserved function in folliculogenesis as found in other vertebrates, while *figla_tv2* may play a role in the spermatogenesis of pseudo-males by regulating the synthesis and metabolism of steroid hormones [53]. Also identified with 1 gene in the black porgy, *sf1* was reported to act as an essential transcriptional factor for steroidogenesis and for development of the reproductive axis (Table 3) [54].

Interestingly, 5 copies of *sox9* were also identified in the black porgy genome. Nevertheless, previous findings reported that only 2 paralogs of *sox9* (*sox9a* and *sox9b*) are present in zebrafish [55] and catfish [56]. Paralog *sox9a* is usually associated with testicular development, while this may be linked with sex reversal in the tong sole [52]. In comparison, *sox9b* possesses a new function in the ovary [55]. In addition, we noticed that female-related genes (*wnt4, vasa*, and *jnk1*) had multiple copies in our current study, which may be retained since the whole-genome duplication in the ancestor of the teleost (Table 3). These genes have been proven to play important roles in ovarian growth and natural sex changes in fishes [57–60]. It was reported that 2 *wnt4* genes (*wnt4a* and *wnt4b*) are present in most teleost fish, while other vertebrates and invertebrates possess only a single *wnt4* gene. Furthermore, 2 copies of *wnt4a*, *wnt4a1*, and *wnt4a2* exist in some teleost species resulting from the additional duplication of the *wnt4* gene [61]. It has been shown that *wnt4a* was mainly expressed in the gonad, gill, and brain of teleost fish (such as zebrafish [62] and rainbow trout [63]), and it was confirmed to be associated with sex reversal in the tongue sole [61]. The *vasa* gene, also called *ddx4*, was reported to play an important role in gametogenesis and germ cell development [64]. Previous findings showed that *vasa* was a single copy gene in the majority of chordates such as zebrafish [65,66]. However, 3 *vasa* genes were also reported in Nile tilapia (*Oreochromis niloticus*) [67]. *Jnk1* is closely associated with ovarian differentiation and development in fish. A previous finding [58] reported that *jnk1* highly transcribed in the ovary of the female ricefield eel (*Monopterus albus*), another teleost with natural sex-change from female to male, and reduced to a substantial level at the subsequent stage of intersex. Hence, the data demonstrated that *jnk1* may play a key role in sexual reversal. Surprisingly, 2 *jnk1* genes (*jnk1a* and *jnk1b*) were reported in the polyploid hybrids of red crucian carp (*Carassius auratus* red var.) and common carp (*Cyprinus carpio* L.) [68].

Interestingly, our data demonstrate that the distribution of these 3 types of genes in the black porgy genome is similar to that in ricefield eel (our unpublished results; data from the *Monopterus* Whole Genome Shotgun Project have been deposited at DDBJ/EMBL/GenBank under accession number AONE00000000). For example, 2 male-related genes (*piwil1* and *piwil2*) are clustered together, while *lin28a* and *rspo1* are adjacent to each other. We also observed that most of these genes are congregated on scaffolds 1, 2, 3, 11, and 15 (Table 3).

## Conclusions

In summary, we sequenced and assembled the whole genome of Chinese black porgy. This is the first genomic report of Sparidae fish. Furthermore, we provided a genomic survey on the 26 genes potentially associated with sex change. The achieved genome data will be helpful for further biological and evolutionary studies. Furthermore, it will be valuable for implementation of molecular breeding, with substantial support from our genomic data, to obtain genetic improvement of this economically important teleost fish.

## Ethics approval and consent to participate

All animal experiments in this study were implemented according to the guidelines of the Animal Ethics Committee and ratified by the Institutional Review Board of Bioethics and Biosafety of BGI, China.

## Availability of supporting data

The raw sequencing reads of all libraries and the transcriptome data have been deposited in the NCBI SRA database (accession numbers SRA541936 and SRA587358). Supporting data are available in the *GigaScience* database, GigaDB [69].

## Competing interests

All authors report no competing interests.

## Abbreviations

BUSCO: Benchmarking Universal Single-Copy Orthologs; CDS: coding sequence; CEGMA: Core Eukaryotic Genes Mapping Approach; CEG: core eukaryotic gene; NCBI, National Center for Biotechnology Information

## Author contributions

Zy.Z., Q.S., and P.X. conceived the project. J.X., C.J., J.Q., F.Z., Hx.L., Hl.L., D.S., Z.R., and J.C. extracted the genomic DNA and performed genome sequencing. K.Z., S.C., Zw.Z., X.Y., J.Z., C.B., and J.L. assembled the genome and analyzed the data. T.G., R.G., and J.X. participated in discussions and provided valuable advice for revision. K.Z., Q.S., Zy.Z., P.X., Zw.Z., and S.C. prepared the manuscript.

## Supplementary Material

GIGA-D-17-00137_Original-Submission.pdf

GIGA-D-17-00137_Revision-1.pdf

GIGA-D-17-00137_Revision-2.pdf

GIGA-D-17-00137_Revision-3.pdf

GIGA-D-17-00137_Revision-4.pdf

Response-to-Reviewer-Comments_Original-Submission.pdf

Response-to-Reviewer-Comments_Revision-1.pdf

Response-to-Reviewer-Comments_Revision-2.pdf

Response-to-Reviewer-Comments_Revision-3.pdf

Reviewer-1-Report_Original-Submission -- Ole K Tørresen 05 Sep 2017 Reviewed

Reviewer-1-Report_Revision-1 -- Ole K Tørresen 07 Nov 2017 Reviewed

Reviewer-2-Report_Original-Submission -- Ingo Braasch 11 Sep 2017 Reviewed

Reviewer-2-Report_Revision-1 -- Ingo Braasch 07 Nov 2017 Reviewed

Reviewer-2-Report_Revision-2 -- Ingo Braasch 08 Dec 2017 Reviewed

## References

[1] Gonzalez EB, Umino T, Nagasawa K Stock enhancement programme for black sea bream, *Acanthopagrus schlegelii* (Bleeker), in Hiroshima Bay, Japan: a review. Aquaculture Research 2008;39(12):1307–15.

[2] Zhang Y, Øverland M, Xie S, Mixtures of lupin and pea protein concentrates can efficiently replace high-quality fish meal in extruded diets for juvenile black sea bream (*Acanthopagrus schlegeli*). Aquaculture 2012;354:68–74.

[3] Guo Z, Zhang W, Zhou Y, Feeding ratio and frequency affects cadmium bioaccumulation in black sea bream *Acanthopagrus schlegeli*. Aquacult Environ Interact 2015;7(2):135–45.

[4] Murata O Studies on the breeding of cultivated marine fishes. Bulletin of Fishery Laboratory, Kinki University 1998;6:1–101.

[5] Kim YS, Biswas A, Ji SC, Phytase in soybean meal diet improves phosphorus availability of hybrid, female red sea bream *Pagrus major*× male black sea bream *Acanthopagrus schlegeli*. Aquaculture Science 2015;63(2):159–67.

[6] Caporaso JG, Lauber CL, Walters WA, Ultra-high-throughput microbial community analysis on the Illumina HiSeq and MiSeq platforms. ISME J 2012;6(8):1621–4. 22402401 10.1038/ismej.2012.8 PMC3400413

[7] Li R, Yu C, Li Y, SOAP2: an improved ultrafast tool for short read alignment. Bioinformatics 2009;25(15):1966–7. 19497933 10.1093/bioinformatics/btp336

[8] Liu B, Shi Y, Yuan J, Estimation of genomic characteristics by analyzing k-mer frequency in denovo genome projects. Quantitative Biology 2013;35(s1-3):62–67.

[9] Luo R, Liu B, Xie Y, SOAPdenovo2: an empirically improved memory-efficient short-read de novo assembler. GigaScience 2012;1(1):18. 23587118 10.1186/2047-217X-1-18 PMC3626529

[10] Boetzer M, Henkel CV, Jansen HJ, Scaffolding pre-assembled contigs using SSPACE. Bioinformatics 2011;27(4):578–9. 21149342 10.1093/bioinformatics/btq683

[11] Howe K, Clark M D, Torroja C F The zebrafish reference genome sequence and its relationship to the human genome. Nature 2013;496(7446):498–503. 23594743 10.1038/nature12111 PMC3703927

[12] Schartl M, Walter R B, Shen Y The genome of the platyfish, *Xiphophorus maculatus*, provides insights into evolutionary adaptation and several complex traits. Nat Genet 2013;45(5):567–72. 23542700 10.1038/ng.2604 PMC3677569

[13] Chen S, Zhang G, Shao C Whole-genome sequence of a flatfish provides insights into ZW sex chromosome evolution and adaptation to a benthic lifestyle. Nat Genet 2014;46(3):253–60. 24487278 10.1038/ng.2890

[14] Xu P, Zhang X, Wang X Genome sequence and genetic diversity of the common carp, *Cyprinus carpio*. Nat Genet 2014;46(11):1212–9. 25240282 10.1038/ng.3098

[15] Wang Y, Lu Y, Zhang Y, The draft genome of the grass carp (*Ctenopharyngodon idellus*) provides insights into its evolution and vegetarian adaptation. Nat Genet 2015;47(6):625–31. 25938946 10.1038/ng.3280

[16] Lien S, Koop BF, Sandve SR, The Atlantic salmon genome provides insights into rediploidization. Nature 2016;533(7602):200–5. 27088604 10.1038/nature17164 PMC8127823

[17] Lin Q, Fan S, Zhang Y, The seahorse genome and the evolution of its specialized morphology. Nature 2016;540(7633):395–9. 27974754 10.1038/nature20595 PMC8127814

[18] Yang J, Chen X, Bai J, The Sinocyclocheilus cavefish genome provides insights into cave adaptation. BMC Biol 2016;14(1):1. 26728391 10.1186/s12915-015-0223-4 PMC4698820

[19] Parra G, Bradnam K, Korf I CEGMA: a pipeline to accurately annotate core genes in eukaryotic genomes. Bioinformatics 2007;23(9):1061–7. 17332020 10.1093/bioinformatics/btm071

[20] Simão FA, Waterhouse RM, Ioannidis P, BUSCO: assessing genome assembly and annotation completeness with single-copy orthologs. Bioinformatics 2015;31(19):3210–2. 26059717 10.1093/bioinformatics/btv351

[21] Tarailo-Graovac M, Chen N Using RepeatMasker to identify repetitive elements in genomic sequences. Current Protocols in Bioinformatics 2009;chapter 4: unit **4** 10. 10.1002/0471250953.bi0410s25 19274634

[22] Maziade M, Bouchard S, Gingras N, Long-term stability of diagnosis and symptom dimensions in a systematic sample of patients with onset of schizophrenia in childhood and early adolescence. II: Positive/negative distinction and childhood predictors of adult outcome. Br J Psychiatry 1996;169(03):371–8. 9004982 10.1192/bjp.169.3.371

[23] Mario S, Oliver K, Irfan G, AUGUSTUS: ab initio prediction of alternative transcripts. Nucl Acids Res 2006;34(Web Server):W435–9. 16845043 10.1093/nar/gkl200 PMC1538822

[24] Burge C, Karlin S Prediction of complete gene structures in human genomic DNA. J Mol Biol 1997;268(1):78–94. 9149143 10.1006/jmbi.1997.0951

[25] Birney E, Clamp M, Durbin R GeneWise and Genomewise. Genome Res 2004;14(5):988–95. 15123596 10.1101/gr.1865504 PMC479130

[26] Trapnell C, Pachter L, Salzberg SL TopHat: discovering splice junctions with RNA-Seq. Bioinformatics 2009;25(9):1105–11. 19289445 10.1093/bioinformatics/btp120 PMC2672628

[27] Trapnell C, Williams BA, Pertea G, Transcript assembly and quantification by RNA-Seq reveals unannotated transcripts and isoform switching during cell differentiation. Nat Biotechnol 2010;28(5):511–5. 20436464 10.1038/nbt.1621 PMC3146043

[28] Elsik CG, Mackey AJ, Reese JT, Creating a honey bee consensus gene set. Genome Biol 2007;8(1):90–105. 10.1186/gb-2007-8-1-r13 PMC1839126 17241472

[29] Finn RD Pfam: the protein families database. Nucl Acids Res 2014;42(D1):D222–30. 24288371 10.1093/nar/gkt1223 PMC3965110

[30] Attwood TK The PRINTS database: A resource for identification of protein families. Brief Bioinform 2002;3(3):252–63. 12230034 10.1093/bib/3.3.252

[31] Bru C, Courcelle E, Beausse Y, The ProDom database of protein domain families: more emphasis on 3D. Nucl Acids Res 2004;33(Database issue):D212–5. 10.1093/nar/gki034 PMC539988 15608179

[32] Letunic I, Copley RR, Schmidt S, SMART 4.0: towards genomic data integration. Nucl Acids Res 2004;32(90001):142D–144. 10.1093/nar/gkh088 PMC308822 14681379

[33] Boeckmann B, Bairoch A, Apweiler R, The SWISS-PROT protein knowledgebase and its supplement TrEMBL in 2003. Nucl Acids Res 2003;31(1):365–70. 12520024 10.1093/nar/gkg095 PMC165542

[34] Hunter S, Apweiler R, Attwood TK, InterPro: the integrative protein signature database. Nucl Acids Res 2009;37(Database):D211–5. 18940856 10.1093/nar/gkn785 PMC2686546

[35] Hingamp P, Broek AE, Stoesser G, The EMBL nucleotide sequence database: contributing and accessing data. Mol Biotechnol 1999;12(3):255–68. 10631682 10.1385/MB:12:3:255

[36] Kanehisa M, Goto S KEGG: kyoto encyclopedia of genes and genomes. Nucl Acids Res 2000;27(1):29–34. 10.1093/nar/27.1.29 PMC148090 9847135

[37] Cunningham F, Amode MR, Barrell D, Ensembl 2015. Nucl Acids Res 2014;43(Database issue):D662–629. 25352552 10.1093/nar/gku1010 PMC4383879

[38] Li L, Stoeckert CJ, Roos DS OrthoMCL: identification of ortholog groups for eukaryotic genomes. Genome Res 2003;13(9):2178–89. 12952885 10.1101/gr.1224503 PMC403725

[39] Edgar RC MUSCLE: multiple sequence alignment with high accuracy and high throughput. Nucl Acids Res 2004;32(5):1792–7. 15034147 10.1093/nar/gkh340 PMC390337

[40] Guindon S, Dufayard JF, Lefort V, New algorithms and methods to estimate maximum-likelihood phylogenies: assessing the performance of PhyML 3.0. Syst Biol 2010;59(3):307–21. 20525638 10.1093/sysbio/syq010

[41] Wu GC, Chang CF The switch of secondary sex determination in protandrous black porgy, *Acanthopagrus schlegeli*. Fish Physiol Biochem 2013;39(1):33–38. 22411079 10.1007/s10695-012-9618-0

[42] Xiao YM, Chen L, Liu J, Contrast expression patterns of *jnk1* during sex reversal of the rice-field eel. J Exp Zool B Mol Dev Evol 2010;314(3):242–56. 19938068 10.1002/jez.b.21332

[43] Webster KA, Schach U, Ordaz A, *Dmrt1* is necessary for male sexual development in zebrafish. Dev Biol 2017;422(1):33–46. 27940159 10.1016/j.ydbio.2016.12.008 PMC5777149

[44] Mount DW Using the basic local alignment search tool (blast). Cold Spring Harb Protoc 2007;2007(7):pdb.top17. 10.1101/pdb.top17 21357135

[45] Wu GC, Du JL, Lee YH, Current status of genetic and endocrine factors in the sex change of protandrous black porgy, *Acanthopagrus schlegeli* (Teleostean). Ann N Y Acad Sci 2005;1040(1):206–14. 15891026 10.1196/annals.1327.026

[46] Wu G C, Chiu P C, Lin C J, Testicular *dmrt1* is involved in the sexual fate of the ovotestis in the protandrous black porgy. Biol Reprod 2012; 86(2). 10.1095/biolreprod.111.095695 22034528

[47] Wu G C, Tomy S, Nakamura M, Dual roles of cyp19a1a in gonadal sex differentiation and development in the protandrous black porgy, *Acanthopagrus schlegeli*. Biol Reprod 2008;79(6):1111–20. 18667752 10.1095/biolreprod.108.069146

[48] Chen S, Zhang G, Shao C, Whole-genome sequence of a flatfish provides insights into ZW sex chromosome evolution and adaptation to a benthic lifestyle. Nat Genet 2014;46(3):253–60. 24487278 10.1038/ng.2890

[49] Cui Z, Liu Y, Wang W, Genome editing reveals *dmrt1* as an essential male sex-determining gene in Chinese tongue sole (*Cynoglossus semilaevis*). Sci Rep 2017;7:42213. 28205594 10.1038/srep42213 PMC5311979

[50] Nanda I, Kondo M, Hornung U A duplicated copy of *dmrt1* in the sex-determining region of the Y chromosome of the medaka, *Oryzias latipes*. Proc Natl Acad Sci 2002; 99(18):11778–83. 12193652 10.1073/pnas.182314699 PMC129345

[51] Masuyama H, Yamada M, Kamei Y *Dmrt1* mutation causes a male-to-female sex reversal after the sex determination by *Dmy* in the medaka. Chromosome Res 2012;20(1):163–76. 22187367 10.1007/s10577-011-9264-x

[52] Dong X, Chen S, Ji X, Molecular cloning, characterization and expression analysis of Sox9a and Foxl2 genes in half-smooth tongue sole (*Cynoglossuss emilaevis*). Acta Oceanol Sin 2011;30(1):68–77.

[53] Li H, Xu W, Zhang N, Two Figla homologues have disparate functions during sex differentiation in half-smooth tongue sole (*Cynoglossus semilaevis*). Sci Rep 2016;6(1):28219. 27313147 10.1038/srep28219 PMC4911598

[54] Xie QP, He X, Sui YN, Haploinsufficiency of SF-1 causes female to male sex reversal in Nile tilapia, *Oreochromis niloticus*. Endocrinology 2016;157(6):2500–14. 27046435 10.1210/en.2015-2049

[55] Rodriguez-Mari A, Yan YL, Bremiller RA, Characterization and expression pattern of zebrafish anti-Müllerian hormone (*Amh*) relative to *sox9a*, *sox9b*, and *cyp19a1a*, during gonad development. Gene Expr Patterns 2005;5(5):655–67. 15939378 10.1016/j.modgep.2005.02.008

[56] Raghuveer K, Garhwal R, Wang DS, Effect of methyl testosterone- and ethynyl estradiol-induced sex differentiation on catfish, *Clarias gariepinus*: expression profiles of DMRT1, cytochrome P450 aromatases and 3 beta-hydroxysteroid dehydrogenase. Fish Physiol Biochem 2005;31(2-3):143–7. 20035448 10.1007/s10695-006-0016-3

[57] Ye D, Lv D, Song P, Cloning and characterization of a rice field eel *vasa-like* gene cDNA and its expression in gonads during natural sex transformation. Biochem Genet 2007;45(3-4):211–24. 17318374 10.1007/s10528-006-9066-6

[58] Xiao YM, Chen L, Liu J, Contrast expression patterns of *jnk1* during sex reversal of the rice field eel. J Exper Zool B 2010;314(3):242–56. 10.1002/jez.b.21332 19938068

[59] Böhne A, Wilson CA, Postlethwait JH, Variations on a theme: genomics of sex determination in the cichlid fish *Astatotilapia burtoni*. BMC Genomics 2016;17(1):883. 27821061 10.1186/s12864-016-3178-0 PMC5100337

[60] Bernard P, Harley V Wnt4 action in gonadal development and sex determination. Int J Biochem Cell Biol 2007;39(1):31–43. 16905353 10.1016/j.biocel.2006.06.007

[61] Hu Q, Zhu Y, Liu Y, Cloning and characterization of *wnt4a* gene and evidence for positive selection in half-smooth tongue sole (*Cynoglossus semilaevis*). Sci Rep 2015;4(1):7167. 10.1038/srep07167 PMC4241513 25418599

[62] Matsui T, Raya Á, Kawakami Y, Noncanonical Wnt signaling regulates midline convergence of organ primordia during zebrafish development. Genes & Development 2005;19(1):164–75. 15630025 10.1101/gad.1253605 PMC540234

[63] Nicol B, Guerin A, Fostier A Ovary-predominant *wnt4* expression during gonadal differentiation is not conserved in the rainbow trout (*Oncorhynchus mykiss*). Mol Reprod Dev 2012;79(1):51–63. 22125114 10.1002/mrd.21404

[64] Lüking A, Stahl U, Schmidt U The protein family of RNA helicases. Crit Rev Biochem Mol Biol 1998;33(4):259–96. 9747670 10.1080/10409239891204233

[65] Yoon C, Kawakami K, Hopkins N Zebrafish *vasa* homologue RNA is localized to the cleavage planes of 2-and 4-cell-stage embryos and is expressed in the primordial germ cells. Development 1997;124(16):3157–65. 9272956 10.1242/dev.124.16.3157

[66] Krøvel AV, Olsen LC Sexual dimorphic expression pattern of a splice variant of zebrafish *vasa* during gonadal development. Dev Biol 2004;271(1):190–7. 15196960 10.1016/j.ydbio.2004.04.004

[67] Fujimura K, Conte MA, Kocher TD Circular DNA intermediate in the duplication of Nile tilapia *vasa* genes. PLoS One 2011;6(12):e29477. 22216289 10.1371/journal.pone.0029477 PMC3245284

[68] Xiao YM, Jiang MG, Luo Z W Identification and analysis of the *jnk1* gene in polyploid hybrids of red crucian carp (*Carassius auratus* red var.) and common carp (*Cyprinus carpio* L.). Genet Mol Res 2014;13(1):906–19. 24634111 10.4238/2014.February.19.1

[69] Zhang Z, Zhang K, Chen S Supporting data for “Draft genome of the protandrous Chinese black porgy, *Acanthopagrus schlegeli*.” GigaScience Database 2018 http://dx.doi.org/10.5524/100409. 10.1093/gigascience/giy012 PMC5893958 29659813

