# Supplementary material for: Draft genome of the protandrous Chinese black porgy, Acanthopagrus schlegelii
Source: Gigascience. 2018 Feb 26;7(4):giy012. doi: 10.1093/gigascience/giy012 (PMC5893958; doi:10.1093/gigascience/giy012)
Supplement: GIGA-D-17-00137_Original-Submission.pdf [file giy012_giga-d-17-00137_original-submission.pdf]

GigaScience

A high-quality genome assembly of the protandrous Chinese black porgy, Acanthopagrus schlegelii

--Manuscript Draft--

**Manuscript Number:**	GIGA-D-17-00137
**Full Title:**	A high-quality genome assembly of the protandrous Chinese black porgy, Acanthopagrus schlegelii
**Article Type:**	Data Note
**Funding Information:**	Nantong Applied Basic Research Program (MS12015071) — Dr. Zhiwei Zhang<br>Key Research and Development (Modern Agriculture) Program of Jiangsu Province (BE2016326) — Dr. Zhiwei Zhang<br>Zhenjiang Leading Talent Program for Innovation and Entrepreneurship — Dr. Qiong Shi<br>Aquatic Sanxin Engineering Major Project of Jiangsu Province (D2015-17) — Dr. Zhiyong Zhang<br>Jiangsu Innovation Ability Construction Program (BM2015017) — Dr. Zhiyong Zhang<br>Aquatic Sanxin Engineering Project of Jiangsu Province (Y2016-23) — Dr. Zhiyong Zhang<br>Nantong Applied Basic Research Program (MS12015070 & MS12016029) — Dr. Zhiyong Zhang

Funding source	Recipient
Nantong Applied Basic Research Program (MS12015071)	Dr. Zhiwei Zhang
Key Research and Development (Modern Agriculture) Program of Jiangsu Province (BE2016326)	Dr. Zhiwei Zhang
Zhenjiang Leading Talent Program for Innovation and Entrepreneurship	Dr. Qiong Shi
Aquatic Sanxin Engineering Major Project of Jiangsu Province (D2015-17)	Dr. Zhiyong Zhang
Jiangsu Innovation Ability Construction Program (BM2015017)	Dr. Zhiyong Zhang
Aquatic Sanxin Engineering Project of Jiangsu Province (Y2016-23)	Dr. Zhiyong Zhang
Nantong Applied Basic Research Program (MS12015070 & MS12016029)	Dr. Zhiyong Zhang

Abstract:

Abstract
Background: As one of the most popular and valuable commercial marine fishes in China and East Asian countries, the Chinese black porgy (Acanthopagrus schlegelii) presents some attractive characteristics, such as high growth rate, good meat quality, resistance to diseases and excellent adaptability to various environments. What's more, the black porgy is a perfect model for investigation on sex change in fish due to its protandrous hermaphrodite. Here, we obtained a high-quality genome of this interesting teleost species, and performed a genomic survey on potential genes associated with the sex-change phenomenon.
Findings: We generated 175.4 gigabases (Gb) of high-quality sequence reads using a whole-genome shotgun sequencing strategy. The final genome assembly is approximately 688 million bases (Mb), accounting for 93% of the estimated genome size (739.6Mb). The achieved Scaffold N50 is 7.64 Mb, reaching a relatively high level among sequenced fish species. Meanwhile, we identified 15,780 protein-coding genes, with an average transcript length of 8.5 Kb. By using a comparative genomics analysis, we figured out 3 types of genes potentially associated with sex change, which are useful for prediction of related genetic basis for the interesting protandrous hermaphrodite.
Conclusion: We provided a high-quality genome assembly of the Chinese black porgy, and discussed about the potential genetic mechanisms of sex change. These data will also offer an important resource for studying the biology as well as facilitating molecular breeding of this economically important fish.

Keywords: Chinese black porgy; Acanthopagrus schlegelii; Whole genome sequencing; Genome assembly; Sex-change related genes

**Corresponding Author:**	Qiong Shi, PhD<br>BGI<br>Shenzhen, CHINA
**Corresponding Author Secondary Information:**	
**Corresponding Author's Institution:**	BGI
**Corresponding Author's Secondary**	

Powered by Editorial Manager® and ProduXion Manager® from Aries Systems Corporation

**Institution:**	
**First Author:**	Qiong Shi, PhD
**First Author Secondary Information:**	
**Order of Authors:**	Qiong Shi, PhD
	Zhiyong Zhang, PhD
	Kai Zhang, PhD
	Shuyin Chen, PhD
	Zhiwei Zhang, PhD
	Xinxin You, PhD
	Jinyong Zhang, PhD
	Chao Bian, PhD
	Jin Xu
	Chaofeng Jia
	Jun Qiang
	Fei Zhu
	Hongxia Li
	Hailin Liu
	Dehua Shen
	Zhonghong Ren
	Jieming Chen
	Jia Li
	Tianheng Gao
	Ruobo Gu
	Junmin Xu
	Pao Xu, PhD
**Order of Authors Secondary Information:**	
**Opposed Reviewers:**	
**Additional Information:**	
**Question**	**Response**
Are you submitting this manuscript to a special series or article collection?	No
**Experimental design and statistics**<br><br>Full details of the experimental design and statistical methods used should be given in the Methods section, as detailed in our Minimum Standards Reporting Checklist. Information essential to interpreting the data presented should be made available in the figure legends.<br><br>Have you included all the information requested in your manuscript?	Yes

Powered by Editorial Manager® and ProduXion Manager® from Aries Systems Corporation

**Resources**<br><br>A description of all resources used, including antibodies, cell lines, animals and software tools, with enough information to allow them to be uniquely identified, should be included in the Methods section. Authors are strongly encouraged to cite Research Resource Identifiers (RRIDs) for antibodies, model organisms and tools, where possible.<br><br>Have you included the information requested as detailed in our Minimum Standards Reporting Checklist?	Yes
**Availability of data and materials**<br><br>All datasets and code on which the conclusions of the paper rely must be either included in your submission or deposited in publicly available repositories (where available and ethically appropriate), referencing such data using a unique identifier in the references and in the “Availability of Data and Materials” section of your manuscript.<br><br>Have you have met the above requirement as detailed in our Minimum Standards Reporting Checklist?	Yes

Powered by Editorial Manager® and ProduXion Manager® from Aries Systems Corporation

Manuscript
Click here to download Manuscript manuscript.docx

A high-quality genome assembly of the protandrous Chinese black porgy, *Acanthopagrus schlegelii*

Zhiyong Zhang[1]†*, Kai Zhang[2,3,4]†, Shuyin Chen[1]†, Zhiwei Zhang[1]†, Xinxin You[3]†, Jinyong Zhang[5]†, Chao Bian[3,6], Jin Xu[1], Chaofeng Jia[1], Jun Qiang[2], Fei Zhu[1], Hongxia Li[2], Hailin Liu[1], Dehua Shen[1], Zhonghong Ren[1], Jieming Chen[3], Jia Li[3], Tianheng Gao[7], Ruobo Gu[3,6], Junmin Xu[3,6], Qiong Shi[3,4,6]*, Pao Xu[2]*

1 Jiangsu Marine Fishery Research Institute, Nantong, Jiangsu 226007, China

2 Freshwater Fishery Research Center, Chinese Academy of Fishery Sciences, Wuxi, Jiangsu 214081, China

3 Shenzhen Key Lab of Marine Genomics, Guangdong Provincial Key Lab of Molecular Breeding in Marine Economic Animals, BGI Academy of Marine Sciences, BGI Marine, BGI, Shenzhen 518083, China

4 BGI Education Center, University of Chinese Academy of Sciences, Shenzhen, Guangdong 518083, China

5 State Key Laboratory of Freshwater Ecology and Biotechnology, Institute of Hydrobiology, Chinese Academy of Sciences, Wuhan, Hubei 430000, China

6 BGI-Zhenjiang Institute of Hydrobiology, Zhenjiang, Jiangsu 212000, China

7 College of Oceanography, Hohai University, Nanjing, Jiangsu 210098, China

* Correspondence address. Pao Xu, Freshwater Fishery Research Center, Chinese Academy of Fishery Sciences, Wuxi, Jiangsu 214081, China (tel: +86-138 0619 0669; email: xup@ffrc.cn) ; Qiong Shi, Shenzhen Key Lab of Marine Genomics, Guangdong Provincial Key Lab of Molecular Breeding in Marine Economic Animals, BGI Academy of Marine Sciences, BGI Marine, BGI, Shenzhen 518083, China (tel:

+86-185 6627 9826; email: shiqiong@genomics.cn); Zhiyong Zhang, Jiangsu Marine Fishery Research Institute, Nantong, Jiangsu 226007, China (tel: +86-13906292412; email: 13906292412@126.com)

† Contributed equally to this work.

Abstract

Background: As one of the most popular and valuable commercial marine fishes in China and East Asian countries, the Chinese black porgy (*Acanthopagrus schlegelii*) also known as the as blackhead seabream, presents some attractive characteristics, such as high growth rate, good meat quality, resistance to diseases and excellent adaptability to various environments. What's more, the black porgy is a perfect model for investigation on sex change in fish due to its protandrous hermaphrodite. Here, we obtained a high-quality genome of this interesting teleost species, and performed a genomic survey on potential genes associated with the sex-change phenomenon.

Findings: We generated 175.4 gigabases (Gb) of high-quality sequence reads using a whole-genome shotgun sequencing strategy. The final genome assembly is approximately 688 million bases (Mb), accounting for 93% of the estimated genome size (739.6Mb). The achieved Scaffold N50 is 7.64 Mb, reaching a relatively high level among sequenced fish species. Meanwhile, we identified 15,780 protein-coding genes, with an average transcript length of 8.5 Kb. By using a comparative genomics analysis, we figured out 3 types of genes potentially associated with sex change, which are useful for prediction of related genetic basis for the interesting protandrous hermaphrodite.

Conclusion: We provided a high-quality genome assembly of the Chinese black porgy, and discussed about the potential genetic mechanisms of sex change. These data will also offer an important resource for studying the biology as well as facilitating molecular breeding of this economically important fish.

Keywords: Chinese black porgy; *Acanthopagrus schlegelii*; Whole genome sequencing; Genome assembly; Sex-change related genes

Data description

Background information

As one of the most popular and valuable commercial marine fishes in China and East Asian countries, the Chinese black porgy (*Acanthopagrus schlegelii*)， also known as the blackhead seabream or sea bread, has presented some interesting characteristics, such as high growth rate, good meat quality, resistance to diseases and excellent adaptability to various environments. It is often farmed for food in the South China Sea and the coastal waters around Japan and Korea [1,2]. In addition, it is an eurythermal and euryhaline fish, living in a wide ranges of water temperatures and salinities. Recently, some basic studies on genetic improvement for its growth and disease resistance have been increasingly performed for an efficient farming purpose [3].

The Chinese black porgy is also an ideal model for investigation on the genetic mechanisms of sex change due to its interesting life cycle, being a functional male at the first 2 years and a subsequent female at the next couple of years. Recently, an excellent hybrid of Japanese seabream (*Pagrosomus major*; ♀) and the Chinese black porgy (♂) is available [4,5], with better growth performance and higher tolerance against low temperature than its parents. However, related genetic mechanisms for these interesting biological characteristics are still unclear. Here, we sequenced and assembled the whole genome of the Chinese black porgy, and performed a genomic survey on potential genes associated with the sex-change phenomenon.

Sample and sequencing

The wild black porgy (NCBI Taxonomy ID: 72011; Fishbase ID: 6531) individuals

(**Figure 1**) were collected from Laizhou Bay in Yantai, Shandong Province, China. All animal experiments in this study were implemented in the light of the guidelines of the Animal Ethics Committee and ratified by the Institutional Review Board on Bioethics and Biosafety of BGI.

Genomic DNA was extracted from the muscle of a female fish using Qiagen GenomicTip100 (Qiagen, Hilden, USA). We used the whole-genome shotgun sequencing strategy and constructed the subsequent 3 short-insert libraries (250-bp, 500-bp and 800-bp) and 4 long-insert libraries (2-kb, 5-kb, 10-kb and 20-kb) in accordance with the standard protocol from Illumina (San Diego, USA). All these constructed libraries were sequenced on the Illumina HiSeq 2000 system [6] (the read length is 125 bp). Finally, we generated a total of 272.9-Gb raw reads from all the 7 libraries.

For improving the quality of sequence reads, those with low-quality bases, adapter sequences and duplicated sequences were discarded. Subsequently, we obtained approximately 175.4 Gb of high-quality clean reads for further genome size prediction and assembling. A *k*-mer analysis with the formula G = *k*_num/*k*_depth [7] was performed to estimate the genome size of Chinese black porgy. In our current study, the achieved total number of *k*-mers and *k*_depth were 2.81×10^{10} and 38, respectively. Therefore, the genome size of Chinese black porgy is estimated to be 739.6 Mb. Based on this result, the retained reads were calculated to cover approximately 238 fold of the whole genome.

Assembly and evaluation

To obtain a good quality of genome assembly, we run SOAPdenovo2 v2.04.4 [8] with optimized parameters (pregraph -K 27 -p 16 -d 1; contig –M 3; scaff -F -b 1.5 -p 16) using these clean reads. In brief, error corrected reads were applied for contig assembly, and all the filtered reads were aligned onto the contigs for linking these contigs to generate scaffolds. GapCloser v1.12 software [9] with default parameters was subsequently used to fill some intra-scaffold gaps in the local assembly, in which

the reads were equipped with one end uniquely mapped to a contig whereas the other end located within a gap. Meanwhile, SSPACE (version 2.0) [10] with optimized parameters (-k 6 -T 4 -g 2) was employed to obtain super scaffolds with the reads from the long-insert libraries (2-kb, 5-kb, 10-kb and 20-kb). The finally generated genome assembly is approximately 688 Mb, which accounts for more 93% of the estimated genome size (739.6Mb; **Table 1**).

The achieved Scaffold N50 is 7.64 Mb, reaching a relatively high level among sequenced fish species, such as 6.4 Mb for grass carp [11], 2.97 Mb for Atlantic salmon [12], 1.8 Mb for a seahorse [13] and 1.15 Mb for a Chinese barbel fish [14]. Core Eukaryotic Genes Mapping Approach (CEGMA; version 2.5) [15] with a set of 248 conserved Core Eukaryotic Genes (CEGs) was employed to assess completeness of the final assembly. The achieved data reveal that our genome assembly possess 228 (92%) of the 248 CEGs. Meantime, Benchmarking Universal Single-Copy Orthologs (BUSCO; version 3) [16] was used to evaluate the quality of the generated genome assembly. The BUSCO score was 87%, containing [D: 4.0%], F: 6.8%, M: 5%, n: 843 (C: complete [D: duplicated], F: fragmented, M: missed, n: genes). These results from CEGMA and BUSCO suggested a high quality of the generated assembly.

Annotation

We used RepeatProteinMask (version 4.0.6) [17] in RepeatMasker to identify the repetitive sequences, and employed RepeatModeller (version 1.05) [18] as well as LTR_FINDER.x86_64-1.0.6 to construct a *de novo* repeat library. Additionally, repeat elements were predicted using Tandem Repeat Finder (TRF, version 4.04). Finally, we observed that the identified repeat sequences cover 23.70% of the assembled genome (**Table 2**), which is relatively low compared with those in other reported fish species, such as 52.2% in zebrafish [19], 25.4% in Atlantic cod [20], and 25.2% in stickleback [21]. It suggests that the Chinese black porgy may have a compact genome.

Prediction of protein coding genes was performed based on the integration of *ab initio* prediction, homologue prediction, and transcriptome-based prediction. The *ab initio*

prediction on the repeat-masked assembly was carried out with Augustus (version2.5) [22] and GENESCAN (version1.0) [23]. For the homology-based gene prediction, homologous proteins of several reported fishes (zebrafish, fugu, stickleback and medaka) were aligned to the assembled genome using TblastN with e-value ≤ 1e-5. Subsequently, all the achieved alignments were analyzed using Genewise (version 2.20) [24] software to search for precise gene structures. We further filtered out these short (less than 150 bp), prematurely terminated or frame-shifted genes. For the transcriptome-based prediction, we obtained transcriptome data from a mixture of liver, muscle, skin, gill and brain of a female fish. Those with low-quality bases, adapter sequences and duplicated sequences were filtered out and we acquired approximately 8 Gb of high-quality clean reads. Subsequently, TopHat1.2 [25] and Cufflinks (version 2.2.1) [26] were applied to predict gene structures using these retained reads. Eventually, the three gene sets generated from the prediction approaches were integrated into a comprehensive and non-redundant gene set using GLEAN [26]. As summarized in **Table 1**, the final gene set contains 15,780 genes, with an average transcript length of 8.5 Kb.

Simultaneously, all the protein sequences from the GLEAN analysis were mapped onto several public databases, including Pfam [28], PRINTS [29], ProDom [30] and SMART [31], to detect the known motifs and domains within our genome assembly. It is proved that more than 90% of these predicted genes from the assembled genome own at least one related functional assignment from other public databases, including Swiss-Prot [32], Interpro [33], TrEMBL [34] and KEGG [35]. These nice data indicate that the achieved gene set for Chinese black porgy is indeed of high quality.

Phylogenetic analysis

In order to examine the phylogenetic position of Chinese black porgy, we downloaded protein sequences of 7 reported fishes, including spotted gar (*Lepisosteus oculatus*), stickleback (*Gasterosteus aculeatus*), Japanses fugu (*Takifugu rubripes*), medaka (*Oryzias latipes*), zebrafish (*Danio rerio*), platyfish (*Xiphophorus maculatus*), and

Nile tilapia (*Oreochromis niloticus*), from Ensembl (release 83) [36]. These sequences were used to construct gene families by OrthoMCL [37] and eventually generated a total of 17,431 gene families by the all-to-all BLASTP strategy with an E-value of 1e-5.

In additional, 65 gene families that were only presented in the black porgy genome and 3,475 single-copy orthologous genes from these gene families were identified. These single-copy genes were subsequently aligned using MUSCLE (version 3.8.31) with default parameters [38], and the protein alignments were changed to corresponding CDS alignments by using an in-house perl script. All the translated CDS sequences of each species were integrated into one “supergene” for a comparative phylogenetic analysis using PhyML [39]. Our final data revealed a close relationship of the black porgy to fugu (**Figure 2**), which also has a compact genome [40].

Analysis of three types of genes for sex change

Sex change (secondary sex determination) is a universal phenomenon in fish, but it usually doesn’t occur in amphibians and mammals. The black porgy is an excellent model for study on the mechanisms of sex change. Protein sequences of 3 main types of genes potentially associated with sex change, including sex determination and differentiation genes, pluripotency factors and apoptosis factors [41,42,43], were downloaded from the NCBI to provide a genomic survey on these genes in the assembled genome using BLAST [44] and Gene Wise [24]. Finally, we detected related homologous sequences of 26 genes in the genome assembly of Chinese black porgy (see more details in **Table 3**).

Previous studies have revealed that multiple genes (*dmrt1*, *cyp19a1a, Wnt4, sox9*, *sf-1, foxl2*, *figla*, *amhr2* and *dax1*) are associated with sex change in the black porgy [41,45], and these sex determination and differentiation genes are existent in our assembled scaffolds (in the first batch of **Table 3**). In current study, the important

male-related *dmrt1* and the steroidogenesis-suppressing factor *dax1* were mapped on scaffold 56 and 14 of the black porgy genome, respectively.

It was reported that *dmrt1* may play a key role in sex change of the black porgy, and male-phase maintenance of male development was regulated by the brain-pituitary-gonadal axis via the GnRH-GtH-Dmrt1 [46]. In the economically important half-smooth tongue sole (*Cynoglossus semilaevis*), *dmrt1* has been proved to be a necessary male sex-determining gene [47,48]. Expression of female-related *foxl2* and *cyp19a1a* were significantly increased in the gonad of ZZ *dmrt1* mutant; in turn, male-related *Sox9a* and *Amh* were significantly decreased [48]. We also validated existence of *foxl2* and *cyp19a1a,* two putative female-related genes, in the black porgy genome. Previous findings revealed that *cyp19a1a* plays dual roles in gonadal development, and both *cyp19a1a* and *foxl2* are related to sex change of the black porgy [49]. However, *Foxl2* has functions in sex differentiation, but is not essential for sex determination and sex change in the tongue sole [50].

Figla, with only one form in the black porgy, is a germ-cell-specific transcription factor related to ovary development and differentiation [49]. However, two isoforms (*Figla_tv1 and Figla_tv2*) were reported in the tongue sole. *Figla_tv1* possesses a conserved function in folliculogenesis as in other vertebrates, while *Figla_tv2* may play a role in the spermatogenesis of pseudomales by regulating the synthesis and metabolism of steroid hormones [51].

Five copies of *sox9* were also identified in the black porgy genome. Nevertheless, previous findings reported only 2 isoforms of *sox9* (*sox9a* and *sox9b*) are present in zebrafish [52] and catfish [53]. *Sox9a* is usually associated with testicular development [45], while in the tong sole may be linked with sex reversal [50]; *sox9b* possesses a new function in ovary [45].

Sf-1, identified with 5 copies in the black porgy (**Table 3**), was reported to act as an essential transcriptional factor for steroidogenesis as well as for development of the

reproductive axis [54]. In addition, we noticed that female-related genes (*Wnt4, vasa and JNK1*) have multiple copies in our current study, which may be resulted from whole-genome duplication. These genes are proved to play important roles in ovarian growth and natural sex change in fishes [55,56,57,58]. It was reported that two *wnt4* genes (*wnt4a* and *wnt4b*) are present in most teleost fish, whereas other vertebrates and invertebrates possess only a single *wnt4* gene [59]. Furthermore, two copies of the *wnt4a*, *wnt4a1* and *wnt4a2*, exist in some teleost species resulting from the additional duplication of *wnt4* gene [60]. *wnt4a* was mainly expressed in the gonad, gill, and brain of teleost fish (such as tongue sole [59], zebrafish [61] and rainbow trout [60]), and it is confirmed to be associated with sex reversal in the tongue sole [59].

Interestingly, our data demonstrate that the distribution of these 3 types of genes in the black porgy genome is similar to that in Chinese ricefield eel (*Monopterus albus*), another teleost with natural sex-change from female to male (our unpublished results; The *Monopterus* Whole Genome Shotgun project has been deposited at DDBJ/EMBL/GenBank under the accession AONE00000000). For example, 2 male-related genes (*piwil1 and piwil2*) are clustered together, and *lin28a* and *rspo1*are adjacent to each other. We also observed that most of these genes are congregated on the scaffold 1, 2, 3, 11 and 15 (**Table 3**), suggesting potential existence of a sex chromosome in the black porgy.

In summary, we sequenced and assembled the whole genome of Chinese black porgy. This is the first genomic report of Sparidae fish. Furthermore, we provided a genomic survey on 26 genes potentially associated with sex change. The achieved genome data will be helpful for further biological and evolutionary studies, and also valuable for implementation of molecular breeding to obtain genetic improvement of this economically important teleost fish.

Table 1. Summary of the achieved genome assembly and annotation.

Genome assembly	
Contig N50 size (kb)	17.2
Contig number (>100bp)	115,091
Scaffold N50 size (Mb)	7.6
Scaffold number (>100bp)	31,359
Total length (Mb)	688.1
Genome coverage (X)	257.6

The longest scaffold (bp)	22,574,836
Genome annotation	
Protein-coding gene number	15,780
Mean transcript length (kb)	8.5
Mean exons per gene	8.7
Mean exon length (bp)	178.5
Mean intron length (bp)	945.2

Table 2. Detailed classification of repeat sequences in the assembled genome.

Type	Repbase TEs		TE proteins		*Denovo*		Combined TEs	
	**Length (Mb)**	**In genome (%)**	**Length (Mb)**	**In genome (%)**	**Length (Mb)**	**In genome (%)**	**Length (Mb)**	**In genome (%)**

DNA	20.930	3.041	2.200	0.320	58.340	8.479	68.130	9.902
LINE	10.240	1.488	6.950	1.010	26.760	3.889	33.020	4.789
SINE	1.120	0.163	2.340	0.000	3.780	0.550	4.550	0.661
LTR	7.200	1.046	35.410	0.340	25.980	3.062	31.270	4.544
Other	0.020	0.003	0.000	0.000	0.000	0.000	0.020	0.003
Unknown	0.000	0.000	0.000	0.000	25.370	3.687	25.370	3.687
Total	35.300	5.130	11.480	1.669	124.540	18.099	136.240	19.780

Table 3. Information of three types of genes for sex change in the black porgy genome

**Sex determination and differentiation genes**		
Gene	Copy number	Scaffold
fst	2	10, 17
Sox9	5	11, 13, 16, 19, 27
vasa	10	11, 14, 16, 20 , 27, 34, 37, 47, 53, 68
Beta-Catenin	4	2, 16, 64, 115
Piwil1	1	15
Piwil2	1	15
Sf-1	5	8, 15, 19, 21, 28
RSPO1	2	2, 74
foxl2	1	22
cyp19a1a	2	8, 28
gsdf	1	3
Figla	1	32
DMRT1	1	56
Wnt4	15	1, 2, 5, 6, 7, 8, 9, 18, 19,

		20, 32, 34, 62 ,67, 122
Dax1	1	14
Cyp11a1	3	2, 8, 33
Hsd3b1	2	7,36
amhr2	1	9
JNK1	12	1, 3, 4, 5, 7, 16, 17, 34, 38, 79, 117, 139
**Pluripotency factors**		
Gene	Copy number	Scaffold
Klf4	2	1, 142
nr5a2	6	8, 10, 15, 19, 21, 28
lin-28a	2	2, 3
4-Oct	1	3
**Apoptosis factors**		
Gene	Copy number	Scaffold
traf2-1	2	3, 15
caspase-2	1	2
tnfr1	1	2

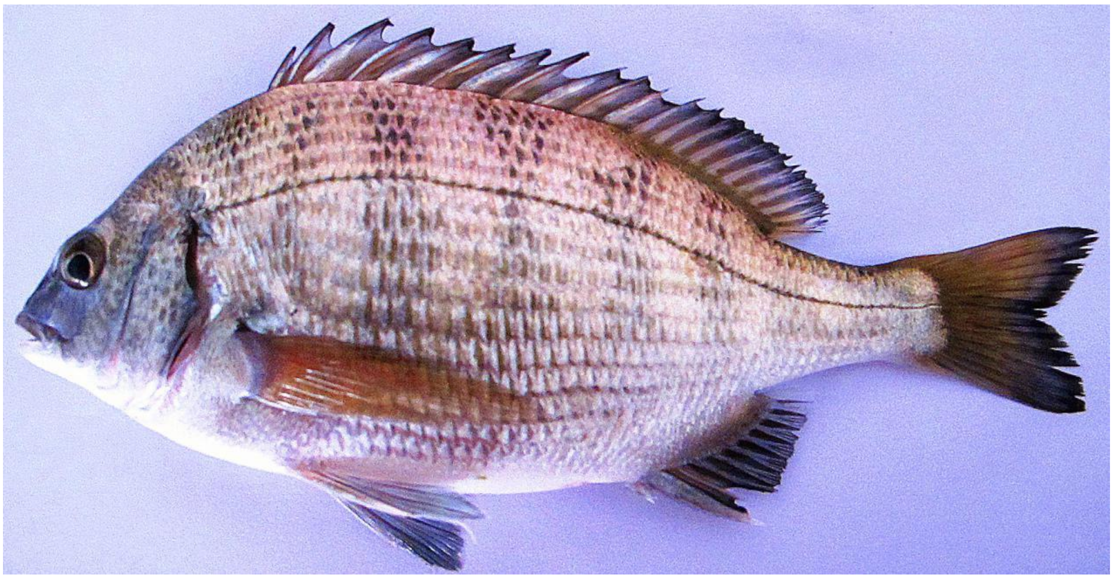

Figure 1. Image of a Chinese black porgy. It was captured from Laizhou Bay in Yantai, Shandong Province, China.

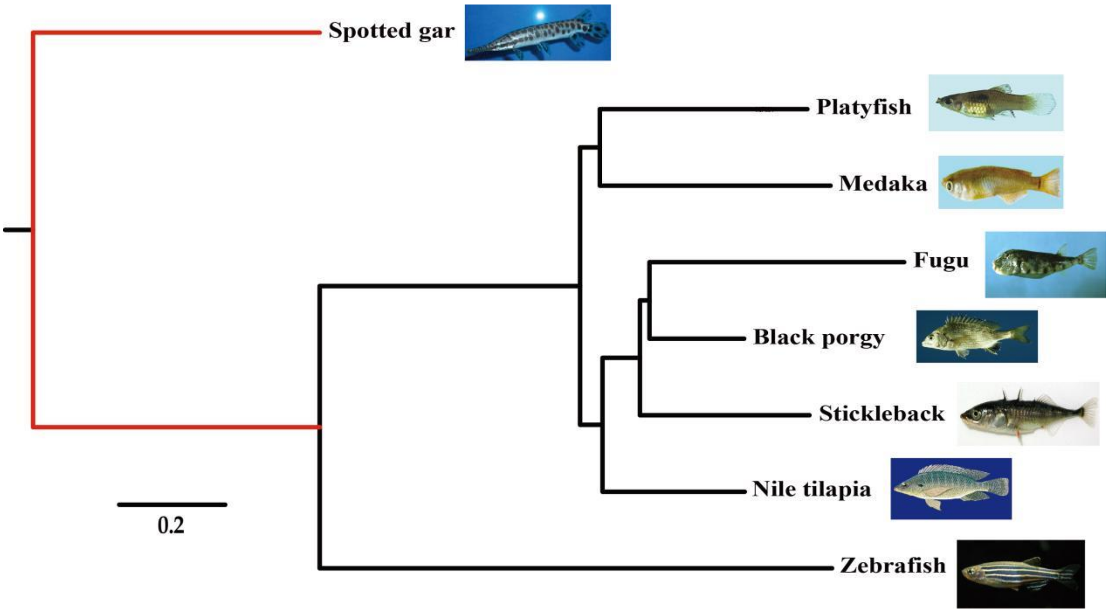

Figure 2. Phylogeny of ray-finned fishes. The Spotted gar was used as the outgroup. These pictures in the phylogenetic tree were downloaded from the Fishbase.

Availability of supporting data

The raw sequencing reads of all libraries and the transcriptome data have been deposited in the NCBI SRA database with accession numbers of SRA541936 and SRA587358. Supporting data are available in the GigaScience database, GigaDB.

Acknowledgements

This work was supported by Aquatic Sanxin Engineering Major Project of Jiangsu Province (No. D2015-17), Key Research and Development (Modern Agriculture) Program of Jiangsu Province (No. BE2016326), Fund for Independent Innovation of Agricultural Science and Technology of Jiangsu Province (No. CX(17)2021), Aquatic Sanxin Engineering Project of Jiangsu Province (No.Y2016-23), Jiangsu Innovation Ability Construction Program (No. BM2015017), Nantong Applied Basic Research Program (No.MS12015071), Nantong Applied Basic Research Program (No. MS12015070 & MS12016029), and Zhenjiang Leading Talent Program for Innovation and Entrepreneurship.

Competing interests

The authors declare that they have no competing interests.

References

1. Gonzalez EB, Umino T, Nagasawa K. Stock enhancement programe for black sea bream, *Acanthopagrus schlegelii* (Bleeker), in Hiroshima Bay, Japan:a Review.Aquaculture Research 2008;**39**:1307-1315.

2. Zhang Y, Øverland M, Xie S et al. Mixtures of lupin and pea protein concentrates can efficiently replace high-quality fish meal in extruded diets for juvenile black sea bream (*Acanthopagrus schlegelii*). Aquaculture 2012;**354**:68-74.

3. Guo Z, Zhang W, Zhou Y et al. Feeding ratio and frequency affects cadmium bioaccumulation in black sea bream *Acanthopagrus schlegelii*. Aquaculture Environment Interactions 2015;**7**(2):135-145.

4. Murata O. Studies on the breeding of cultivated marine fishes. Bull. Fish.Lab. Kinki Univ 1998;**6**:1-101.

5. Kim YS, Biswas A, Ji SC et al. Phytase in soybean meal diet improves phosphorus availability of hybrid, female red sea bream *Pagrus major*× male black sea bream *Acanthopagrus schlegelii*. Aquaculture Science 2015;**63**(2):159-167.

6. Caporaso JG, Lauber CL, Walters WA et al. Ultra-high-throughput microbial community analysis on the Illumina HiSeq and MiSeq platforms. The ISME Journal 2012;**6**(8):1621-1624.

7. Liu B, Shi Y, Yuan J et al. Estimation of genomic characteristics by analyzing k-mer frequency in denovo genome projects. Quantitative Biology 2013;**35(**s1–3):62-67.

8. Luo R, Liu B, Xie Y et al. SOAPdenovo2: an empirically improved memory-efficient short-read de novo assembler. Gigascience 2012;**1**:18.

9. Li R, Yu C, Li Y et al. SOAP2: an improved ultrafast tool for short read alignment. Bioinformatics 2009;**25(**15):1966-1967.

10. Boetzer M, Henkel CV, Jansen HJ et al. Scaffolding pre-assembled contigs using SSPACE. Bioinformatics 2011;**27**:578–579.

11. Wang Y, Lu Y, Zhang Y et al. The draft genome of the grass carp (*Ctenopharyngodon idellus*) provides insights into its evolution and vegetarian adaptation. Nature genetics 2015;**47**(6):625-631.

12. Lien S, Koop BF, Sandve SR et al. The Atlantic salmon genome provides insights into rediploidization. Nature 2016;**533**:200-205.

13. Lin Q, Fan S, Zhang Y et al. The seahorse genome and the evolution of its specialized morphology. Nature 2016;**540**(7633):395-399.

14. Yang J, Chen X, Bai J et al. The Sinocyclocheilus cavefish genome provides insights into cave adaptation. BMC Biology 2016;**14**(1):1.

15. Parra G, Bradnam K, Korf I. CEGMA: a pipeline to accurately annotate core genes in eukaryotic genomes. Bioinformatics 2007;**23**(9):1061-1067.

16. Simão FA, Waterhouse RM, Ioannidis P et al. BUSCO: assessing genome assembly and annotation completeness with single-copy orthologs. Bioinformatics 2015;31(**19**):3210-3212.

17. Tarailo-GraovacM, Chen N. Using RepeatMasker to identify repetitive elements in Genomic sequences. Current Protocols in Bioinformatics 2009;chapter 4: unit **4** 10.

18. Maziade M, Bouchard S, Gingras N et al. Long-term stability of diagnosis and symptom dimensions in a systematic sample of patients with onset of schizophrenia in childhood and early adolescence. II: Postnegative distinction and childhood predictors of adult outcome. The British Journal of Psychiatry 1996;**169**(3):371-378.

19. Howe K, Clark MD, Torroja CF et al. The zebrafish reference genome sequence and its relationship to the human genome. Nature 2013;**496**(7446):498-503.

20. Star B, Nederbragt AJ, Jentoft S et al. The genome sequence of Atlantic cod reveals a unique immune system. Nature 2011;**477**(7363):207-210.

21. Jones F C, Grabherr M G, Chan Y F et al. The genomic basis of adaptive evolution in threespine sticklebacks. Nature 2010;**484**(7392):55-61.

22. Mario S, Oliver K, Irfan G et al. AUGUSTUS: ab initio prediction of alternative transcripts. Nucleic Acids Research 2006;**34**:435-439.

23. Burge C, Karlin S. Prediction of complete gene structures in human genomic DNA. Journal of Molecular Biology 1997;**268**(1):78-94.

24. Birney E, Clamp M, Durbin R. GeneWise and Genomewise. Genome Research 2004;**14**(5):988-995.

25. Trapnell C, Pachter L, Salzberg SL. TopHat: discovering splice junctions with RNA-Seq. Bioinformatics 2009;**25**(9):1105-1111.

26. Trapnell C, Williams BA, Pertea G et al. Transcript assembly and quantification by RNA-Seq reveals unannotated transcripts and isoform switching during cell differentiation. Nature Biotechnology 2010;**28**(5):511-515.

27. Elsik CG, Mackey AJ, Reese JT et al. Creating a honey bee consensus gene set. Genome Biology 2007;**8**(1):90-105.

28. Finn RD. Pfam: the protein families database. Nucleic Acids Research 2014;**42**(Database issue):D222-230.

29. Attwood TK. The PRINTS database: A resource for identification of protein families. Briefings in Bioinformatics 2002;**3**(3):252-263.

30. Bru C, Courcelle E, Beausse Y et al. The ProDom database of protein domain families: more emphasis on 3D. Nucleic Acids Research 2005;**33**(Database issue):212-215.

31. Letunic I, Copley RR, Schmidt S et al. SMART 4.0: towards genomic data integration. Nucleic Acids Research 2004;**32**(Databaseissue):D142-D144.

32. Boeckmann B, Bairoch A, Apweiler R et al. The Swiss-Prot knowledgebase and its supplement TREMBL in 2003. Nucleic Acids Research 2003;**31**(1):365-370.

33. Hunter S, Apweiler R, Attwood TK et al. InterPro: the integrative protein signature database. Nucleic Acids Research 2009;**37**(suppl 1):D211-D215.

34. Hingamp P, Broek AEVD, Stoesser G et al. The EMBL nucleotide sequence database. Molecular Biotechnology 1999;**12**(3):255-267.

35. Kanehisa M, Goto S. KEGG: kyoto encyclopedia of genes and genomes. Nucleic Acids Research 2000;**27**(1):29-34(26).

36. Cunningham F, Amode MR, Barrell D et al. Ensembl 2015. Nucleic Acids Research 2014;**43**(Database issue):D662-629.

37. Li L, Stoeckert CJ, Roos DS. OrthoMCL: identification of ortholog groups for eukaryotic genomes. Genome Research 2003;**13**(9):2178-2189.

38. Edgar RC. MUSCLE: multiple sequence alignment with high accuracy and high throughput. Nucleic Acids Research 2004;**32**(5):1792-1797.

39. Guindon S, Dufayard JF, Lefort V et al. New algorithms and methods to estimate maximum-likelihood phylogenies: assessing the performance of PhyML 3.0. Systematic Biology 2010;**59**(3):307-321.

40. Aparicio S, Chapman J, Stupka E et al. Whole-genome shotgun assembly and analysis of the genome of Fugu rubripes. Science 2002;**297**(5585):1301-1310.

41. Wu GC, Chang CF. The switch of secondary sex determination in protandrous black porgy, *Acanthopagrus schlegeli*. Fish Physiology and Biochemistry 2013;**39**(1):33-38.

42. Xiao YM, Chen L, Liu J et al. Contrast expression patterns of *JNK1* during sex reversal of the rice-field eel. Journal of Experimental Zoology Part B: Molecular and Developmental Evolution 2010;**314**(3):242-256.

43. Webster KA, Schach U, Ordaz A et al. *Dmrt1* is necessary for male sexual

development in Zebrafish. Developmental Biology 2017;**422**(1):33-46.

44. Mount DW. Using the basic local alignment search tool (blast). Cold Spring Harbor Protocols 2007;**2007**(7):pdb.top17.

45. Wu GC, Du JL, Lee YH et al. Current status of genetic and endocrine factors in the sex change of protandrous black porgy, *Acanthopagrus schlegeli* (Teleostean). Annals of the New York Academy of Sciences 2005;**1040**(1):206-214.

46. Wu G C, Chiu P C, Lin C J et al. Testicular dmrt1 is involved in the sexual fate of the ovotestis in the protandrous black porgy. Biology of reproduction 2012; **86**(2).

47. Chen S, Zhang G, Shao C et al. Whole-genome sequence of a flatfish provides insights into ZW sex chromosome evolution and adaptation to a benthic lifestyle. Nature genetics 2014;**46**(3):253-260.

48. Cui Z, Liu Y, Wang W et al. Genome editing reveals dmrt1 as an essential male sex-determining gene in Chinese tongue sole (*Cynoglossus semilaevis*). Scientific Reports 2017;**7**:42213.

49. Wu G C, Tomy S, Nakamura M et al. Dual roles of cyp19a1a in gonadal sex differentiation and development in the protandrous black porgy, *Acanthopagrus schlegeli*. Biology of reproduction 2008;**79**(6):1111-1120.

50. Dong X, Chen S, Ji X et al. Molecular cloning, characterization and expression analysis of Sox9a and Foxl2 genes in half-smooth tongue sole (*Cynoglossus semilaevis*). Acta Oceanologica Sinica 2011;**30**(1):68-77.

51. Li H, Xu W, Zhang N et al. Two Figla homologues have disparate functions during sex differentiation in half-smooth tongue sole (*Cynoglossus semilaevis*). Scientific reports 2016;**6**:28219.

52. Rodrı´guez-Marı´ A, Yan YL, Bremiller RA et al. Characterization and expression pattern of zebrafish Anti-Mu¨llerian hormone (Amh) relative to sox9a,

sox9b, and cyp19a1a, during gonad development. Gene Expression Patterns 2005;**5**:655–667.

53. Raghuveer K, Garhwal R, Wang DS et al. Effect of methyl testosterone-and ethynyl estradiol-induced sex differentiation on catfish, *Clarias gariepinus*: expression profiles of DMRT1, Cytochrome P450aromatases and 3 beta-hydroxysteroid dehydrogenase. Fish Physiology and Biochemistry 2005;**31**(2):143-147.

54. Xie QP, He X, Sui YN et al. Haploinsufficiency of SF-1 Causes Female to Male Sex Reversal in Nile Tilapia, *Oreochromis niloticus*. Endocrinology 2016;**157**(6):2500-2514.

55. Ye D, Lv D, Song P et al. Cloning and characterization of a rice field eel vasa-like gene cDNA and its expression in gonads during natural sex transformation. Biochemical Genetics 2007;**45**(3-4):211-224.

56. Xiao YM, Chen L, Liu J et al. Contrast expression patterns of JNK1 during sex reversal of the rice field eel. Journal of Experimental Zoology Part B 2010;**314**(3):242-256.

57. Böhne A, Wilson CA, Postlethwait JH et al. Variations on a theme:Genomics of sex determination in the cichlid fish *Astatotilapia burtoni*. BMC Genomics 2016;**17**(1):883.

58. Bernard P, Harley V. Wnt4 action in gonadal development and sex determination. The International Journal of Biochemistry & Cell Biology 2007;**39**(1):31–43.

59. Hu Q, Zhu Y, Liu Y et al. Cloning and characterization of wnt4a gene and evidence for positive selection in half-smooth tongue sole (*Cynoglossus semilaevis*). Scientific reports 2014;**4**:7167.

60. Nicol B, Guerin A, Fostier A, et al. Ovary-predominant wnt4 expression during gonadal differentiation is not conserved in the rainbow trout (*Oncorhynchus mykiss*). Molecular reproduction and development 2012;**79**(1):51-63.

61. Matsui T, Raya Á, Kawakami Y et al. Noncanonical Wnt signaling regulates midline convergence of organ primordia during zebrafish development. Genes & development 2005;**19**(1):164-175.
