# Supplementary material for: Draft genome of the protandrous Chinese black porgy, Acanthopagrus schlegelii
Source: Gigascience. 2018 Feb 26;7(4):giy012. doi: 10.1093/gigascience/giy012 (PMC5893958; doi:10.1093/gigascience/giy012)
Supplement: GIGA-D-17-00137_Revision-1.pdf [file giy012_giga-d-17-00137_revision-1.pdf]

GigaScience

Draft Genome of the Protandrous Chinese Black Porgy, Acanthopagrus schlegelii

--Manuscript Draft--

**Manuscript Number:**	GIGA-D-17-00137R1
**Full Title:**	Draft Genome of the Protandrous Chinese Black Porgy, Acanthopagrus schlegelii
**Article Type:**	Data Note
**Funding Information:**	Aquatic Sanxin Engineering Major Project of Jiangsu Province (D2015-17) — Dr. Zhiyong Zhang<br>Jiangsu Innovation Ability Construction Program (BM2015017) — Dr. Zhiyong Zhang<br>Nantong Applied Basic Research Program (MS12015071) — Dr. Zhiwei Zhang<br>Key Research and Development (Modern Agriculture) Program of Jiangsu Province (BE2016326) — Dr. Zhiwei Zhang<br>Aquatic Sanxin Engineering Project of Jiangsu Province (Y2016-23) — Dr. Zhiyong Zhang<br>Nantong Applied Basic Research Program (MS12015070 & MS12016029) — Dr. Zhiyong Zhang<br>Zhenjiang Leading Talent Program for Innovation and Entrepreneurship — Dr. Qiong Shi
**Abstract:**	Abstract<br>Background: As one of the most popular and valuable commercial marine fishes in China and East Asian countries, the Chinese black porgy (Acanthopagrus schlegelii) also known as the blackhead seabream, has some attractive characteristics, such as fast growth rate, good meat quality, resistance to diseases and excellent adaptability to various environments. Furthermore, the black porgy is a good model for investigating sex changes in fish due to its protandrous hermaphrodite. Here, we obtained a high-quality genome assembly of this interesting teleost species and performed a genomic survey on potential genes associated with the sex-change phenomenon.<br>Findings: We generated 175.4 gigabases (Gb) of clean sequence reads using a whole-genome shotgun sequencing strategy. The final genome assembly is approximately 688.1 megabases (Mb), accounting for 93% of the estimated genome size (739.6 Mb). The achieved scaffold N50 is 7.6 Mb, reaching a relatively high level among sequenced fish species. Meanwhile, we identified 19,465 protein-coding genes, which had an average transcript length of 17.3 kb. By performing a comparative genomic analysis, we found three types of genes potentially associated with sex change, which are useful for the prediction of related genetic basis for the interesting protandrous hermaphrodite.<br>Conclusions: We provided a draft genome assembly of the Chinese black porgy and discussed about the potential genetic mechanisms of sex change. These data are also an important resource for studying the biology and facilitating the molecular breeding of this economically important fish.
**Corresponding Author:**	Qiong Shi, PhD<br>BGI<br>Shenzhen, CHINA
**Corresponding Author Secondary Information:**	
**Corresponding Author's Institution:**	BGI
**Corresponding Author's Secondary Institution:**	
**First Author:**	Qiong Shi, PhD
**First Author Secondary Information:**	

Powered by Editorial Manager® and ProduXion Manager® from Aries Systems Corporation

**Order of Authors:**	Qiong Shi, PhD
	Zhiyong Zhang, PhD
	Kai Zhang, PhD
	Shuyin Chen, PhD
	Zhiwei Zhang, PhD
	Xinxin You, PhD
	Jinyong Zhang, PhD
	Chao Bian, PhD
	Jin Xu
	Chaofeng Jia
	Jun Qiang
	Fei Zhu
	Hongxia Li
	Hailin Liu
	Dehua Shen
	Zhonghong Ren
	Jieming Chen
	Jia Li
	Tianheng Gao
	Ruobo Gu
	Junmin Xu
	Pao Xu, PhD
**Order of Authors Secondary Information:**	
**Response to Reviewers:**	Dear Editor,<br>Thanks for your kind help. We also appreciate the instructive comments from the two reviewers. According to their suggestions, we performed additional analyses and made a careful revision on our previous manuscript. Our point-by-point responses are attached for your consideration.<br>We removed the conclusion about compact genome and revised the “high quality” statement. Addition of the section on Author’s contributions was done in accordance with your advice. By the way, the current version was revised with help from MDPI, a professional English editing company.<br>Best regards,<br>Qiong Shi, PhD, Professor<br>BGI<br>Shenzhen 518083<br>China<br><br>Reviewer 1:<br>## General comments ##<br>Hermaphroditism is an interesting method of reproduction, where individuals change sex during their lifetime, or produce both eggs and sperm at the same time. While the genotypic basis for sex-determination in gonochoristic species (those that do not change sex) is being elucidated in more and more species, knowledge about the genotypic basis for sex change in hermaphrodite fishes is currently lacking.<br><br>The authors produce a genome assembly of the protandrous (male first) Chinese black porgy, Acanthopagrus schlegelii, annotates it, and do a survey of genes that are known to be involved in sex determination and differentiation. I greatly support publication of genome assemblies, and teleost genome assemblies in particular. The more data

Powered by Editorial Manager® and ProduXion Manager® from Aries Systems Corporation

available for analysis for scientists, the better. However, I find the claims regarding the high quality of the genome assembly to be unsubstantiated. Quality is a difficult measure, but if it has to be used, then it has to be shown that something is better than something else, or at least equal to something of high quality. If the authors had shown that more complete genes are found with CEGMA and BUSCO than in other teleost genome assemblies, then I would have been convinced that this assembly is of high quality. They do not do that. As the genome assembly is presented in this manuscript, it does not warrant the modifier 'high quality'.

Answer: Thanks for your comments. You are right. We should compare our results, for example the scaffold N50 value, with others. We also replaced “high quality” with a statement of “a relatively high level”. Please see more details about related changes on lines 111-116 of the revised manuscript.

The authors also claim that black porgy has a compact genome, but do not shown much data that supports this. It has a similar genome size to many other teleosts, such as medaka, platyfish, Atlantic cod, Amazon molly, and substantially larger than species with known compact genomes such as fugu, tetraodon, stickleback and pipefishes. If the authors want to make a point of the supposed compactness of the black porgy genome, they need to make a much stronger case than this.

Answer: Thanks for your advice. The claim about compact genome was removed in the revised manuscript.

The authors do a survey of different genes associated with sex determination and differentiation in different species, but it is not clear to me why this is done. No further analysis than stating that these are present was done. Was the presence of these genes surprising? Then that should be stated properly. While the methods of sex determination and differential might not be conserved through teleosts, I do think we would expect the presence of most of these genes in most teleosts. In addition, the purpose for this analysis needs to be justified. Was it performed as a quality control of the genome assembly?

Answer: Thanks for your comments and advice. In fact, some genes have been confirmed to be involved in sex change of the black porgy (lines 188-190). We mentioned them for a quality control of the genome assembly, as you mentioned, and also provide more information (such as scaffold location and copy number, see more details in Table 3) to support the sex-change phenomenon. The latter will be definitely helpful for further investigation on molecular mechanisms of sex change. Please find more explanations on lines 177-241 of the revised manuscript. Interestingly, we also proposed possible existence of sex chromosome in this teleost fish (lines 239-241).

The specific programs, version and settings used for some of the analyses are not stated. Please address this because it is important for assessing the method and approach used, in addition to reproducibility.

Answer: Thanks for your nice advice. Information about version and settings for each specific program was provided in the revised manuscript.

I cannot recommend publication of this manuscript before the issues I highlight above and below are addressed.

Specific comments

Abstract:
Please do not use abbreviations such as "what's" in the abstract.

Answer: Yes, it is done. Thanks for your advice. Please find corresponding change (Furthermore) on line 36 of the revised manuscript.

I am not sure that the black porgy is a "perfect" model for investigating sex change. It might very well be a good model.

Answer: Thanks for your advice. We changed "perfect" to "good" on line 36 of the

revised manuscript.

Please change "…due to its protandrous hermaphrodite" to something like "…due to it being a protandrous hermaphrodite".

Answer: Yes, it is done. Please find corresponding change on line 37 of the revised manuscript.

Please distinguish between a genome and a genome assembly. You can obtain a high-quality genome assembly, but not a high quality genome.

Answer: Yes, you are right. Please find corresponding change on line 38 of the revised manuscript.

How do the authors assess "high-quality sequence reads"? High quality compared to what?

Answer: Thanks for your question. In fact, before assembly of the sequencing reads, SOAPfilter v2.2 software (Li et al. 2009) [7] with default parameters (-y -p -g 1 -o clean -M 2 -f 0) was utilized to remove low-quality raw reads (including reads with 10 or more non-sequenced/low-quality bases), PCR duplicates and adaptor sequences. We hence changed “high-quality” to “clean” on line 40 and added related procedure on lines 88-91 of the revised manuscript.

Please use lower case "scaffold" instead of capital letter in "…achieved Scaffold N50…".

Answer: Yes, it is done (on lines 43 and 111 of the revised manuscript).

Please use correct SI system units. Capital letter K, such as in Kb, is not a valid SI-system prefix (seehttps://en.wikipedia.org/wiki/Metric_prefix if unsure). kb is the correct abbreviation.

Answer: Thanks for your good advice. We corrected them on lines 45 and 150 of the revised manuscript.

The last sentence of the findings part of the abstract should be rewritten to more correct and accurate English.

Answer: Thanks for your nice comments. We rewrote the sentence in the revised manuscript (on lines 45-48) as follows.
By performing a comparative genomic analysis, we found three types of genes potentially associated with sex change, which are useful for the prediction of related genetic basis for the interesting protandrous hermaphrodite.

How do the authors assess the high quality of the genome assembly? High quality compared to what?

Answer: Thanks for your good question. In fact, we ran CEGMA and BUSCOv2 using the actinopterygii dataset on our genome assembly to evaluate the genome quality. The CEGMA results suggest that 90.7% CEGs are complete and 92.3% are partial; The BUSCO score was 91.0%, (C:91.0% [S:88.1%,D:2.9%], F:1.1%, M:7.9%, n:4584). We also compared our results, for example the scaffold N50 value, with others. We already replaced “high quality” with a statement of “a relatively high level” on lines 111-124. Hence, here we provided a draft genome assembly of the Chinese black porgy (please find the statement on line 49 of our revised manuscript).

Data description:
How do the authors assess that the hybrid of Japanese seabream and Chinese black porgy is "excellent"? It can be "good" or "important", but I am unsure of how it can be "excellent".

Answer: Thanks for your advice. We changed "excellent" to "good" on line 70 of the revised manuscript.

I find the description of "improving the quality of sequencing reads" lacking. First, it is not possible to improve the quality of these. The sequencing machine sets the quality of these. I would have written something along the lines of "Before assembly of the sequencing reads, those with low-quality bases…". How was the removal of these reads done? Which program and which settings? Again, in what way is the resulting clean reads of "high-quality"? Is it necessary to specify the quality of these?

Answer: Thanks for the nice comments. We changed “high-quality” to “clean” on line 40. We also added the following sentence in the revised manuscript (on lines 88-91) for a clear statement.
Before assembly of the sequencing reads, SOAPfilter v2.2 software (Li et al. 2009) [7] with default parameters (-y -p -g 1 -o clean -M 2 -f 0) was utilized to remove low-quality raw reads (including reads with 10 or more non-sequenced/low-quality bases), PCR duplicates and adaptor sequences.

How can it be stated that the result will be a good quality genome assembly before running SOAPdenovo2? Better skip the word "quality" here, and just write "To obtain a genome assembly, we ran SOAPdenovo2…"

Answer: Thanks for your nice advice. Yes, it is done. Please find the change on line 99 of the revised manuscript.

In the assembly section, the authors suddenly mention "error corrected [sic]" reads, without mentioning how these were created. Please describe how the error-correction was performed, which program and which settings.

Answer: Thanks for your suggestions. Sorry for the mistake. The sentence was rewritten on lines 101-103, and a clear statement was added on lines 88-91 of the revised manuscript.

Why applying SSPACE on the scaffolds created by SOAPdenovo2? Does it create even longer and better scaffolds? How was the optimized parameters found?

Answer: Yes, the SSPACE was employed to create longer and better scaffolds. In order to find the optimized parameters, we tested multiple different settings. The -K option of SOAPdenovo2 was set at 25, 27, 29, 31 and 35 for trial. Finally, we observed that the –K set at 27 could generate a good genome assembly. The other parameters of SOAPdenovo were defaulted without change.

Again, "scaffold" with lower case first letter, and not capital.

Answer: Yes, it is done (on line 111 of the revised manuscript).

I like that CEGMA and BUSCO was used to validate the genome assembly. However, it is not specified whether or not the 92 % of CEGs found were complete or not. 92 % partial hits of CEGs are not especially impressive, but 92 % of complete hits are of course better.

Answer: The sentence was changed as follows on line 119.
The estimates suggest that 90.7% CEGs are complete and 92.3% are partial.
Hence, we stated that we obtained a draft genome of the black porgy (line 49).

In addition it is not specified which dataset was used for BUSCO. For BUSCOv3, the possible datasets (also called assessment sets) are Eukaryota, Metazoa, Vertebrata or Actinopterygii. However, while BUSCO version 3 is mentioned, it does not look like version 3 was used. From the number of genes found (n: 843), it looks like version 1 of BUSCO was run on the metazoan dataset, see Simão et al 2015. Since BUSCOv3 is mentioned, I would rather see the performance on the Actinopterygii dataset (found at http://busco.ezlab.org/) with BUSCOv3. Then http://www.biorxiv.org/content/early/2017/08/17/177485 should also be referred, where BUSCOv3 is described. For examples of performance of BUSCO on fish genome assembles, see for instance Jansen et al 2017 with BUSCOv3 run on the European eel genome assembly with the vertebrate dataset, finding 79.8 % complete genes (of

2586), or Liu et al 2017 where they find 81.4% complete and 9.1% partial vertebrate BUSCO genes (of 3023) using
BUSCOv1 in the blunt snout bream genome assembly.
With the availability of the genome assembly, I downloaded it and ran CEGMA and BUSCOv2 using the actinopterygii dataset on it. While the results might vary from run to run or computer to computer, I find 86.3 % complete and 89.1 % partial genes with CEGMA. With BUSCOv2 I find 91.0 % complete genes (C:91.0%[S:88.1%,D:2.9%],F:1.1%,M:7.9%,n:4584). This is less than some of the genome assemblies I have looked at, but the authors should do their own comparisons.
After addressing these two issues (partial or complete CEGMA, correct dataset and version of BUSCO), then the authors can then assess whether or not the genome assembly is of high quality by comparing to other fish genome assemblies, for instance those two mentioned or other relevant ones.

Answer: Thanks for your comments. According to your suggestions, we ran CEGMA and BUSCOv2 using the actinopterygii dataset on our genome assembly. The CEGMA estimates suggest that 90.7% CEGs are complete and 92.3% are partial.

Table A. Statistics of the completeness of the genome based on 248 CEGS.
ProtsCompleteness (%)
Complete22590.73
Group 16293.94
Group 24885.71
Group 35590.16
Group 46092.31
Partial 22992.34
Group 16395.45
Group 24987.50
Group 35793.44
Group 46092.31

The final BUSCO score was 91.0%, (C:91.0% [S:88.1%, D:2.9%], F:1.1%, M:7.9%, n:4584). These results from CEGMA and BUSCO suggested the assembled genome covers majority of the gene space.
You are right. The CEGMA and BUSCO scores are good, but not outstanding. We hence revised these sentences (on lines 119-124 of the new manuscript).

It is good a de novo repeat library was created. However, direct comparisons of repeat content between different species are not straightforward. For instance, the difference of 23.70 % repeats in black porgy compared to 25.4 % in Atlantic cod and 25.2 % in stickleback is likely not significant. Different approaches would give different numbers. Also, at 739.6 Mbp Chinese black porgy might have a smaller genome than some other fishes, but it is not especially compact compared to fugu, tetraodon, stickleback or the different pipefish and sea horse genomes.

Answer: Thanks for the nice comment. We removed these sentences about comparisons of repeat content between different fish species in the revised manuscript.

The authors might confuse GENSCAN (identifying gene structures) with GENESCAN (analysis of sequencing data from ABI sequencer).

Answer: Sorry for the mistake. It was corrected on line 134 of the revised manuscript.

Use of transcriptome data can greatly improve the annotation of a genome assembly, but I am missing a better description of the sequencing data. Which program and which settings were used to remove low-quality bases, adapter and duplicated sequences? I am a bit surprised that TopHat1.2 was used. From the website (http://ccb.jhu.edu/software/tophat/index.shtml) it seems it was released in 2011. While researchers should keep up to date on which versions of relevant software is used, there might be specific reasons why some software used is out-of-date. However, 6 years old software is a bit of a stretch. Please use a newer version, and consider using HISAT2 instead.

Powered by Editorial Manager® and ProduXion Manager® from Aries Systems Corporation

Answer: Thanks for the nice advice. SOAPfilter v2.2 software (Li et al. 2009) [7] with default parameters (-y -p -g 1 -o clean -M 2 -f 0) was utilized to remove low-quality raw reads (including reads with 10 or more non-sequenced/low-quality bases), PCR duplicates and adaptor sequences. According to your suggestion, the newer version (TopHat2.1.1) was applied to predict gene structures. Please find more details about the changes on lines 88-92 and 147-147 of the revised manuscript.

The low number of annotated genes surprised me. Many fish species usually have about 20,000 genes or more annotated. These seem few and short, and I wonder if something went wrong during the annotation process. For instance, annotated fish genome assemblies in Ensembl of similar size (platyfish, medaka, and Amazon molly) have mean gene lengths around 13 - 17 kbp. However, the authors write that the average transcript length is 8.5 Kb (should be kb). In addition, the average number of exons per gene in those species is 11-15, while in black porgy it is 8.7. Too much of the assembly might have been masked for instance. I would like the authors to address this by doing a good validation of the annotation or redoing the annotation if it is faulty. BUSCO can also be used to validate the annotation, and I downloaded the predicted CDS and ran BUSCO on them. I found 65.4 % complete genes using the actinopterygii dataset (C:65.4% [S:63.0%, D:2.4%], F:4.6%, M:30.0%, n:4584). I am unsure how this compares to other species. From my experience, it is not unusual to find fewer genes in the predicted transcriptome than in the genome assembly.

Answer: Thanks for the nice comments. Annotation of the genome assembly was redone. The final gene set contains 19,465 genes, with an average transcript length of 17.3 kb (on lines 148-149 of the revised manuscript).
We ran BUSCOv2 again on the predicted CDS, and the final BUSCO score was 85.2% (C:85.2% [S:82.2%, D:3.0%], F:2.9%, M:11.9%, n:4584). For the genome assembly, the BUSCO score reached to 91.0%, (C:91.0% [S:88.1%, D:2.9%], F:1.1%, M:7.9%, n:4584).
By the way, please find more details about Annotation on lines 125-156.

I protest to the use of "proved" and "nice" when describing functional motifs and domains in the predicted genes/proteins. Rather write that "90 % of the predicted genes from the assembled genome contain at least one related functional…". The last sentence of this paragraph (starting with "These nice data...") can be skipped.

Answer: Thanks for your nice advice. We rewrote this sentence (on lines 154-156 of the new manuscript) and removed the last sentence of this paragraph in the revised manuscript.

Phylogenetic analysis:
Why was the phylogenetic analysis done? Was the placement of black porgy among teleosts uncertain? I guess that Betancur-R et al 2017 might have come out too late to be used in the writing of this manuscript, but in that publication several other Acanthopagrus species are investigated and placed in the phylogeny. It is puzzling to perform an analysis without using it to draw any conclusions (expectations in gene content for instance).

Answer: Yes, the phylogenetic analysis, on a genomic view, was performed to investigate the placement of black porgy among teleosts. We removed certain conclusions since they were not supported with solid evidence.

Was the predicted transcript sequence for each protein used, or was each amino acid in the protein sequence from the MUSCLE alignment changed into the most common codon? If the latter, that would be strange and a lot of signal would be lost, if the first, that is a valid and proper approach. After looking at the script, it seems that the first is the approach used here. Please describe this section better.

Answer: Thanks for your nice advice. You are right. The protein alignments were changed to corresponding transcript sequences using an in-house perl script. All these nucleotide sequences of each species were integrated into a supergene, which were used to build a phylogenetic tree using PhyML. Please find the corresponding changes on lines 167-172 of the revised manuscript.

Powered by Editorial Manager® and ProduXion Manager® from Aries Systems Corporation

I do not agree with the assertion that black porgy has a "close relationship" with fugu. These two species are separated by almost 100 million years of evolution (Betancur-R et al 2017) and are not close. The presumed closeness of these two species does not seem to affect any of the conclusions or speculations in this paper. Of the species included in the phylogeny, platyfish and medaka both have around 600-700 Mbp size genome assemblies, tilapia and zebrafish have a bit larger (1-1.4 Gbp), while stickleback and fugu have smaller assemblies around 400-450 Mbp. It seems strange to compare directly to fugu when the size of the genome of black porgy is 200-300 Mbp larger, and quite similar in size to medaka and platyfish. That is, it is not especially compact.

Answer: Thanks for the nice comments. We reconstructed the phylogenetic tree and the bootstrap supporting value for the topology was set at 100. We also referred to the phylogenetic tree, which was built by Betancur-R et al and found that several other Acanthopagrus species (Acanthopagrus latus, Acanthopagrus catenula) have a closer relationship with fugu than other species investigated in this study.
You are right, the genome of Porgy is not especially compact. Hence, we removed this sentence in the revised manuscript.

Analysis of three types of genes for sex change:
What is the purpose of this analysis? Were all of the genes downloaded from NCBI found in the genome assembly? How was homology asserted? Homology presumes common ancestry, which would need to be tested in some way. A highly confident BLAST hit could be enough for this, but the authors do not state what cut-offs were used.

Answer: Thanks for your comments.
The black porgy is a good model for investigation on the molecular mechanisms of sex change. In this present study, we provided a genomic survey on these genes associated with sex-change phenomenon in the assembled genome, which will offer a useful genetic resource for studying the interesting protandrous hermaphrodite.
Protein sequences of the three main types of genes potentially associated with sex change were downloaded from NCBI and used for homology searches against the black porgy genome with tBlastn (version2.2.19). We chose alignments with coverage > 70% and identity > 70% for further prediction of gene structures using Genewise (version 2.2.0).
Please find corresponding changes on lines 179-186 of the revised manuscript.

The authors report that Wnt4, vasa and JNK1 are found in multiple copies in the genome assembly, and claim that these might be related to whole-genome duplication. Is it the teleost specific whole-genome duplication they think of, or do they propose additional genome duplication in the lineage leading to black porgy? If the latter, this should be investigated in more detail and properly argued. There are some genome duplications in teleosts, the lineage leading to carps and salmonids are two well-known examples.

Answer: Thanks for your advice. Black porgy is a diploid species, hence multiple copies of wnt4, vasa and JNK1 in the genome assembly may be resulted from the teleost-specific whole genome duplication. Although the current assembly is the first draft of black porgy genome, more investigations are on-going.

This manuscript lacks several declaration sections. While it is stated in the text that "All animal experiments in this study were implemented in the light of the guidelines of the Animal Ethics Committee and ratified by the Institutional Review Board on Bioethics and Biosafety of BGI", it would be good to have this as a separate section also.

Answer: Yes, it’s done. Please find the separate section on lines 319-322 of the revised manuscript.

With 22 authors, and 6 of them contributing equally, I would also like to see an "author's contributions" section.

Answer: Yes, it’s done. Please find the separate section of Author’s contributions on

Powered by Editorial Manager® and ProduXion Manager® from Aries Systems Corporation

lines 327-332 of the revised manuscript.

Reviewer #2:
The present Data Note reports the genome assembly of the black porgy, a teleost fish species of economic importance for the fisheries industry and of interest to understand ontogenetic sex change among vertebrates.

Standard methods have been used to generate a genome assembly of acceptable quality and the dataset has re-use potential. However, there are several issues that should be addressed before the manuscript is ready for publication. Please see a list below.
In addition, the level of writing needs to be improved with the help of a native speaker.

Answer: Thanks for your advice. The current version was revised with help from MDPI, a professional English editing company.

Specific points:
l. 61: confirm that "sea bread" is indeed a common name for this species

Answer: Sorry for the mistake. It was removed on line 60 of the revised manuscript.

l. 103 and l. 109/110: How were the parameters for SOAPdenovo and SSPACE optimized?

Answer: Thanks for the good question. In fact, in order to find the optimized parameters, we tested multiple different settings. The -K option of SOAPdenovo2 was originally set at 25, 27, 29, 31 and 35 for trial. Finally, we observed that the –K set at 27 could generate a good genome assembly. The other parameters of SOAPdenovo were defaulted without any change. For the SSPACE, we employed its default parameters. We corrected this section with more information on lines 99-101 & 106-108 of the revised manuscript.

l. 114-116: The other fish genome assemblies to which the black porgy assembly is compared appear random. Why did you choose these few assemblies for comparison?

Answer: Thanks for the nice comments. In fact, we referred to the scaffold N50 values of multiple representative fish genome assemblies, which were published in good journals such as Nature and Nature Genetics. More data about zebrafish, platyfish, coelacanth, half-smooth tongue sole, elephant shark and common carp were also provided in the revised manuscript (lines 111-116).

l. 117-124: The CEGMA and BUSCO scores are good, but not outstanding. It is difficult to grasp the quality of the assembly from just providing these numbers and the authors could for example obtain CEGMA/BUSCO scores from the other fish genomes in l.114-116 for a more meaningful evaluation and comparison.

Answer: Thanks for your good advice. Based on your advice, we revised the sentence as follows: These results from CEGMA and BUSCO suggested the assembled genome covered majority of the gene space. Please find the corresponding change on lines 123-124 of the revised manuscript.

l.129-133: I do not follow the conclusion that the black porgy has a particularly compact genome and is prominently sparse in repeat content. It is within the range of e.g. cod and stickleback, while zebrafish is a known outlier with an unusual high repeat content. The estimated porgy genome size also does not imply a specifically compact genome.

Answer: Sorry for the misunderstanding. We remove the statement of compact genome in the revised manuscript.

l. 138: How did you obtain the "homologous proteins of several reported fish"?

Answer: The homologous proteins of several reported fishes, including zebrafish, Japanese puffer, stickleback and medaka, were downloaded from Ensembl release 75.

Powered by Editorial Manager® and ProduXion Manager® from Aries Systems Corporation

Please find the new information on lines 135-138 of the revised manuscript.

l. 142-144: At which level did you generate the mixture of transcriptomic data. Did you mix mRNA, cDNA, RNA-seq libraries or reads in silico?

Answer: Sorry for the misleading description. In fact, we mixed cDNAs from these tissues, which was transcribed from mRNAs. Please see more details on lines 141-143 of the revised manuscript.

Phylogenomic analysis:
- How did you chose the species to include in the analysis?
- Please provide bootstrap values for your tree?
- Do you obtain a similar tree with different, e.g. Bayesian, methods?
- How does the obtained tree compare to the known phylogenetic position of black porgy?

Answer: Thanks for the nice comments. The selected fish species were representative and widely used for genomics study since their data are very good. In additional, we provided bootstrap value for the tree in the revised manuscript (lines 172-173). According to your suggestion, we reconstructed the phylogenetic tree using MrBayes (Version 3.2), and obtained the same tree topology from both the Bayesian phylogenetic and the PhyML phylogenetic methods. We also referred to the phylogenetic tree built by Betancur-R et al and found that several other Acanthopagrus species (Acanthopagrus latus, Acanthopagrus catenula) have a closer relationship with fugu. Hence we removed the statement about closer relationship to fugu.

The grouping of porgy with pufferfish leads the authors to imply that there is shared genome compaction among them. Is this supported by knowledge about genome compaction in the pufferfish lineage? Again, I don't agree with the conclusions that the porgy genome is particularly compact.

Answer: You are right, hence we removed the sentence about compact genome in the revised manuscript.

l. 195-197: It would be important to mention that a duplicate of dmrt1 is the male sex determination gene in medaka.

Answer: Yes, it’s done. Please find the addition of such information on lines 201-203 of the revised manuscript.

l. 213: The term isoform should only be used for splice variants and not for gene duplicates such as sox9a and sox9b, which were generated during the teleost genome duplication.

Answer: You are right. We changed “isoforms” to “isotypes” on lines 210 & 216 of the revised manuscript.

Please follow the established gene nomenclature for fish genes.

Answer: Yes, it’s done. Thanks for your advice.

Table 3: Gene name should be oct4, not 4-Oct.

Answer: Sorry for the mistake. We corrected it in the revised Table 3 of the new manuscript.

Analysis of candidate genes for sex determination and sex change:
It is curious that there are a number of genes in the list with multiple copies in the assembly. Please provide more details on these cases. Are these extra copies different alleles, copy number variants or potential redundancy artifacts of the genome assembly? How do these extra copies relate to the teleost whole genome duplication and other known duplications of these genes in fish and vertebrates in general? Finally, it would be good to provide the predicted sequences of the candidate genes as a separate sequence file.

Powered by Editorial Manager® and ProduXion Manager® from Aries Systems Corporation

	Answer: Thanks for the nice comments. Several genes, such as Wnt4, vasa and JNK1, with multiple copies in the assembly may be copy number variants. We cannot rule out potential redundancy artifacts of the genome assembly, hence we are going to clone them before submission to NCBI for public availability.<br><br>l. 230-238: Is the similarity of genetic linkage of some of the sex-related genes in porgy and rice eel a specific characteristic of these two lineages or are they rather an indication of conserved synteny among teleost fishes in general?<br>Please explain better how genes distributed among several scaffolds (1, 2, 3, 11, 15) are a potential indication for a sex chromosome. Also, would you expect a species with ontogenetic sex change to have a sex chromosome at all? Please explain.<br><br>Answer: Sorry, we didn't check the synteny conservation between the two fish species because the ricefield eel work has not been published yet. However, although we didn't expect existence of a sex chromosome, it is still possible since these genes are distributed on only a few of scaffolds. The data are similar to ore previously reported Asian arowana (Bian et al., 2016, Scientific Reports, 6:24501), which was identified with sex chromosomes.
**Additional Information:**	
**Question**	**Response**
Are you submitting this manuscript to a special series or article collection?	No
**Experimental design and statistics**<br><br>Full details of the experimental design and statistical methods used should be given in the Methods section, as detailed in our Minimum Standards Reporting Checklist. Information essential to interpreting the data presented should be made available in the figure legends.<br><br>Have you included all the information requested in your manuscript?	Yes
**Resources**<br><br>A description of all resources used, including antibodies, cell lines, animals and software tools, with enough information to allow them to be uniquely identified, should be included in the Methods section. Authors are strongly encouraged to cite Research Resource Identifiers (RRIDs) for antibodies, model organisms and tools, where possible.<br><br>Have you included the information requested as detailed in our Minimum Standards Reporting Checklist?	Yes
**Availability of data and materials**	Yes

Powered by Editorial Manager® and ProduXion Manager® from Aries Systems Corporation

All datasets and code on which the conclusions of the paper rely must be either included in your submission or deposited in publicly available repositories (where available and ethically appropriate), referencing such data using a unique identifier in the references and in the "Availability of Data and Materials" section of your manuscript.<br><br>Have you have met the above requirement as detailed in our Minimum Standards Reporting Checklist?	

Manuscript Click here to download Manuscript manuscript.docx

Draft Genome of the Protandrous Chinese Black Porgy, *Acanthopagrus schlegelii*

Zhiyong Zhang[1]†*, Kai Zhang[2,3,4]†, Shuyin Chen[1]†, Zhiwei Zhang[1]†, Xinxin You[3]†, Jinyong Zhang[5]†, Chao Bian[3,6], Jin Xu[1], Chaofeng Jia[1], Jun Qiang[2], Fei Zhu[1], Hongxia Li[2], Hailin Liu[1], Dehua Shen[1], Zhonghong Ren[1], Jieming Chen[3], Jia Li[3], Tianheng Gao[7], Ruobo Gu[3,6], Junmin Xu[3,6], Qiong Shi[3,4,6]*, Pao Xu[2]*

1 Jiangsu Marine Fishery Research Institute, Nantong, Jiangsu 226007, China

2 Freshwater Fishery Research Center, Chinese Academy of Fishery Sciences, Wuxi, Jiangsu 214081, China

3 Shenzhen Key Lab of Marine Genomics, Guangdong Provincial Key Lab of Molecular Breeding in Marine Economic Animals, BGI Academy of Marine Sciences, BGI Marine, BGI, Shenzhen 518083, China

4 BGI Education Center, University of Chinese Academy of Sciences, Shenzhen, Guangdong 518083, China

5 State Key Laboratory of Freshwater Ecology and Biotechnology, Institute of Hydrobiology, Chinese Academy of Sciences, Wuhan, Hubei 430000, China

6 BGI-Zhenjiang Institute of Hydrobiology, Zhenjiang, Jiangsu 212000, China

7 College of Oceanography, Hohai University, Nanjing, Jiangsu 210098, China

* Correspondence address. Pao Xu, Freshwater Fishery Research Center, Chinese Academy of Fishery Sciences, Wuxi, Jiangsu 214081, China (tel: +86-138 0619 0669; email: xup@ffrc.cn) ; Qiong Shi, Shenzhen Key Lab of Marine Genomics, Guangdong Provincial Key Lab of Molecular Breeding in Marine Economic Animals, BGI Academy of Marine Sciences, BGI Marine, BGI, Shenzhen 518083, China (tel: +86-185 6627 9826; email: shiqiong@genomics.cn); Zhiyong Zhang, Jiangsu Marine Fishery Research Institute, Nantong, Jiangsu 226007, China (tel: +86-13906292412; email: 13906292412@126.com)

† Contributed equally to this work.

Abstract

Background: As one of the most popular and valuable commercial marine fishes in China and East Asian countries, the Chinese black porgy (*Acanthopagrus schlegelii*) also known as the blackhead seabream, has some attractive characteristics, such as fast growth rate, good meat quality, resistance to diseases and excellent adaptability to various environments. Furthermore, the black porgy is a good model for investigating sex changes in fish due to its protandrous hermaphrodite. Here, we obtained a high-quality genome assembly of this interesting teleost species and performed a genomic survey on potential genes associated with the sex-change phenomenon.

Findings: We generated 175.4 gigabases (Gb) of clean sequence reads using a whole-genome shotgun sequencing strategy. The final genome assembly is approximately 688.1 megabases (Mb), accounting for 93% of the estimated genome size (739.6 Mb). The achieved scaffold N50 is 7.6 Mb, reaching a relatively high level among sequenced fish species. Meanwhile, we identified 19,465 protein-coding genes, which had an average transcript length of 17.3 kb. By performing a comparative genomic analysis, we found three types of genes potentially associated with sex change, which are useful for the prediction of related genetic basis for the interesting protandrous hermaphrodite.

Conclusions: We provided a draft genome assembly of the Chinese black porgy and discussed about the potential genetic mechanisms of sex change. These data are also an important resource for studying the biology and facilitating the molecular breeding of this economically important fish.

Keywords: Chinese black porgy; *Acanthopagrus schlegelii*; whole genome sequencing; genome assembly; sex-change related genes

Data description

Background information

As one of the most popular and valuable commercial marine fishes in China and East

Asian countries, the Chinese black porgy (*Acanthopagrus schlegelii*), also known as the blackhead seabream, has some interesting characteristics, such as fast growth rate, good meat quality, resistance to diseases and good adaptability to various environments. It is often farmed for food in the South China Sea and the coastal waters around Japan and Korea [1,2]. In addition, it is an eurythermal and euryhaline fish, living in a wide range of water temperatures and salinities. Recently, some basic studies on the genetic improvement for its growth and disease resistance have been increasingly performed in order to increase efficiency of farming [3].

The Chinese black porgy is also a good model for investigating the genetic mechanisms of sex change due to its interesting life cycle. It is a functional male during the first 2 years and a subsequent female during the next couple of years. Recently, a good hybrid of the Japanese seabream (*Pagrosomus major*; ♀) and the Chinese black porgy (♂) has become available [4,5], with better growth performance and higher tolerance against low temperature than its parents. However, related genetic mechanisms for these interesting biological characteristics are still unclear. Here, we sequenced and assembled the whole genome of the Chinese black porgy, before performing a genomic survey on potential genes associated with the sex-change phenomenon.

Sample and Sequencing

The wild black porgy (NCBI Taxonomy ID: 72011; Fishbase ID: 6531) individuals (**Figure 1**) were collected from Laizhou Bay in Yantai, Shandong Province, China. Genomic DNA was extracted from the muscle of a female fish using Qiagen GenomicTip100 (Qiagen, Hilden, USA). We employed the whole-genome shotgun sequencing strategy and constructed the subsequent three short-insert libraries (250-bp, 500-bp and 800-bp) and four long-insert libraries (2-kb, 5-kb, 10-kb and 20-kb) in accordance with the standard protocol from Illumina (San Diego, USA). All these constructed libraries were sequenced on the Illumina HiSeq 2000 system [6] (the read length is 125 bp). Finally, we generated a total of 272.9-Gb raw reads from all seven libraries.

Before assembly of the sequencing reads, SOAPfilter v2.2 software [7] with default parameters (-y -p -g 1 -o clean -M 2 -f 0) was utilized to remove low-quality raw reads (including reads with 10 or more non-sequenced/low-quality bases), PCR duplicates and adaptor sequences. Subsequently, we obtained approximately 175.4 Gb of clean reads for further genome size prediction and assembling. A *k*-mer analysis with the formula G = *k*_num/*k*_depth [8] was performed to estimate the genome size of Chinese black porgy. In our current study, the achieved total number of *k*-mers and *k*_depth was 2.81×10^{10} and 38, respectively. Therefore, the genome size of Chinese black porgy is estimated to be 739.6 Mb. Based on this result, the retained reads were calculated to cover approximately 238-fold of the whole genome.

Assembly and Evaluation

To obtain a genome assembly, we employed the SOAPdenovo2 v2.04.4 [9] with optimized parameters (pre-graph -K 27 -p 16 -d 1; contig –M 3; scaff -F -b 1.5 -p 16) using these clean reads. In brief, the reads from short-insert libraries were applied for the contig assembly, before alignment of all the filtered reads onto the contigs for linking these contigs to generate scaffolds. GapCloser v1.12 software [7] with default parameters was subsequently used to fill some intra-scaffold gaps in the local assembly, in which the reads were equipped with one end uniquely mapped to a contig and the other end located within a gap. Meanwhile, SSPACE (version 2.0) [10] with default parameters was employed to obtain super scaffolds with the reads from the long-insert libraries (2-kb, 5-kb, 10-kb and 20-kb). The final genome assembly was approximately 688.1 Mb, which accounts for 93.0% of the estimated genome size (739.6 Mb; **Table 1**).

The achieved scaffold N50 is 7.64 Mb, reaching a relatively high level among sequenced fish species. In comparison, other scaffolds have levels of 1.55 Mb for the zebrafish [11], 1.1 Mb for platy fish [12], 924 kb for coelacanth [13], 867 kb for half-smooth tongue sole [14], 4.5 Mb for elephant shark [15], 1 Mb for common carp [16], 6.4 Mb for grass carp [17], 2.97 Mb for Atlantic salmon [18], 1.8 Mb for a seahorse [19] and 1.15 Mb for a Chinese barbel fish [20]. Core Eukaryotic Genes

Mapping Approach (CEGMA; version 2.5) [21] with a set of 248 conserved Core Eukaryotic Genes (CEGs) was employed to assess the completeness of the final assembly. The estimates suggest that 90.7% CEGs are complete and 92.3% are partial. Meanwhile, Benchmarking Universal Single-Copy Orthologs (BUSCO; version 2) [22] was employed to evaluate the quality of the generated genome assembly. The final BUSCO score was 91.0%, (C:91.0% [S: 88.1%, D: 2.9%], F: 1.1%, M: 7.9% and n: 4584). These results from CEGMA and BUSCO suggested that the assembled genome covers the majority of the gene space.

Annotation

We used RepeatProteinMask (version 4.0.6) [23] in RepeatMasker to identify the repetitive sequences, before employing RepeatModeller (version 1.05) [24] and LTR_FINDER.x86_64-1.0.6 to construct a *de novo* repeat library. Additionally, repetitive elements were predicted using Tandem Repeat Finder (TRF, version 4.04). Finally, we observed that the identified repeat sequences cover 19.78% of the assembled genome (**Table 2**).

Prediction of protein-coding genes was performed based on the integration of *ab initio* prediction, homologue prediction and transcriptome-based prediction. The *ab initio* prediction was carried out with Augustus (version2.5) [25] and GENSCAN (version1.0) [26] on the repeat-masked assembly. For the homology-based gene prediction, homologous proteins of several reported fishes (zebrafish, Japanese puffer, stickleback and medaka) were downloaded from Ensembl release 75 and aligned to the assembled genome using tBlastn (version2.2.19) with e-value $\leq$ $1e^{-5}$. Subsequently, all the achieved alignments were analyzed using Genewise (version2.2.0) software [27] to search for precise gene structures. We further filtered out these short (less than 150 bp), prematurely terminated or frame-shifted genes. For the transcriptome-based prediction, we obtained transcriptome data from a mixture of liver, muscle, skin, gill and brain of a female fish at cDNA level. Those with low-quality bases, adapter sequences and duplicated sequences were removed and we acquired approximately 8 Gb of high-quality clean reads. Subsequently, TopHat2.1.1

[28] and Cufflinks (version 2.2.1) [29] were applied to predict gene structures using these retained reads. Eventually, the three gene sets generated from the prediction approaches were integrated into a comprehensive and non-redundant gene set using GLEAN [30]. As summarized in **Table 1**, the final gene set contains 19,465 genes, with an average transcript length of 17.3 kb.

Simultaneously, all the protein sequences from the GLEAN analysis were mapped onto several public databases, including Pfam [31], PRINTS [32], ProDom [33] and SMART [34], to detect the known motifs and domains within our genome assembly. The data demonstrated that 99.3% of the predicted genes from the assembled genome contain at least one related functional assignment from other public databases, including Swiss-Prot [35], Interpro [36], TrEMBL [37] and KEGG [38].

Phylogenetic Analysis

In order to examine the phylogenetic position of the Chinese black porgy, we downloaded protein sequences of seven reported fishes, including spotted gar (*Lepisosteus oculatus*), stickleback (*Gasterosteus aculeatus*), Japanses fugu (*Takifugu rubripes*), medaka (*Oryzias latipes*), zebrafish (*Danio rerio*), platyfish (*Xiphophorus maculatus*), and Nile tilapia (*Oreochromis niloticus*) from Ensembl (release 83) [39]. These sequences were used to construct gene families by OrthoMCL [40] and eventually generated a total of 17,431 gene families by the all-to-all BLASTP strategy with an E-value of $1e^{-5}$. In additional, 65 gene families were only presented in the black porgy genome.

Subsequently, 3,239 single-copy orthologous genes from these gene families were selected. These single-copy genes were further aligned using MUSCLE (version 3.8.31) with default parameters [41], before the protein alignments were changed to corresponding coding sequences (CDS) using an in-house perl script. All these nucleotide sequences of each species were integrated into a supergene, which were used to build a phylogenetic tree with PhyML [42]. The bootstrap supporting value for the topology is 100. Our final data revealed a close relationship of the black porgy to fugu (**Figure 2**) [43] and also suggested a lower neutral evolutionary rate than any

other investigated teleost.

Analysis of Three Types of Genes for Sex Change

Sex change (secondary sex determination) is a universal phenomenon in fish, but it usually does not occur in amphibians and mammals. The black porgy is a good model for the study on the molecular mechanisms of sex change. For providing a genomic survey on these genes in the assembled genome, protein sequences of three main types of genes potentially associated with sex change, including sex determination and differentiation genes, pluripotency factors and apoptosis factors [44–46], were downloaded from the NCBI database and used for homology searches against the black porgy genome with tBlastn (version2.2.19) [47]. We chose alignments with coverage > 70% and identity > 70% for further prediction of gene structures using Genewise (version 2.2.0) [27]. Finally, we obtained homologous sequences of 26 genes in the genome assembly of Chinese black porgy (see more details in **Table 3**).

Previous studies have revealed that multiple genes, including *dmrt1*, *cyp19a1a*, *Wnt4*, *sox9*, *sf-1*, *foxl2*, *figla*, *amhr2* and *dax1*, are associated with sex change in the black porgy [44,48]. These sex determination and differentiation genes were also found in our assembled scaffolds (in the first batch of **Table 3**). In the current study, the important male-related *dmrt1* and the steroidogenesis-suppressing factor *dax1* were mapped on the scaffolds 56 and 14 of the black porgy genome, respectively.

It was reported that *dmrt1* may play a key role in the sex change of the black porgy, while the male-phase maintenance of male development was regulated by the brain–pituitary–gonadal axis via the GnRH–GtH–Dmrt1 pathway [49]. In the economically important half-smooth tongue sole (*Cynoglossus semilaevis*), *dmrt1* has been proved to be a necessary male sex-determining gene [50,51]. Expression of female-related *foxl2* and *cyp19a1a* were significantly increased in the gonad of ZZ *dmrt1* mutants. In turn, the male-related *Sox9a* and *Amh* were significantly decreased [51]. Moreover, previous findings suggest that a duplicate of *dmrt1* is the male sex determination gene in medaka and *dmrt1* mutation causes a male–female sex reversal [52,53]. We also validated the existence of *foxl2* and *cyp19a1a*, two

putative female-related genes, in the black porgy genome. Previous findings revealed that *cyp19a1a* plays dual roles in gonadal development, while both *cyp19a1a* and *foxl2* are related to the sex change of the black porgy [54]. However, *Foxl2* has functions in sex differentiation, but it is not essential for sex determination and sex change in the tongue sole [55].

Figla, with only one gene in the black porgy, is a germ-cell-specific transcription factor related to ovary development and differentiation [54]. However, two isotypes (*Figla_tv1* and *Figla_tv2*) were reported in the tongue sole. *Figla_tv1* possesses a conserved function in folliculogenesis as found in other vertebrates, while *Figla_tv2* may play a role in the spermatogenesis of pseudo-males by regulating the synthesis and metabolism of steroid hormones [56].

Five copies of *sox9* were also identified in the black porgy genome. Nevertheless, previous findings reported that only 2 isotypes of *sox9* (*sox9a* and *sox9b*) are present in zebrafish [57] and catfish [58]. *Sox9a* is usually associated with testicular development [48], while this may be linked with sex reversal in the tong sole [51]. In comparison, *sox9b* possesses a new function in the ovary [48].

Sf-1, identified with 5 copies in the black porgy (**Table 3**), was reported to act as an essential transcriptional factor for steroidogenesis and for development of the reproductive axis [59]. In addition, we noticed that female-related genes (*Wnt4*, *vasa* and *JNK1*) have multiple copies in our current study, which may be from whole-genome duplication. These genes have been proven to play important roles in ovarian growth and natural sex changes in fishes [60–63]. It was reported that two *wnt4* genes (*wnt4a* and *wnt4b*) are present in most teleost fish, while other vertebrates and invertebrates possess only a single *wnt4* gene [64]. Furthermore, two copies of the *wnt4a*, *wnt4a1* and *wnt4a2*, exist in some teleost species resulting from the additional duplication of *wnt4* gene [64]. It has been shown that *wnt4a* was mainly expressed in the gonad, gill and brain of teleost fish (such as tongue sole [60], zebrafish [65] and rainbow trout [66]), and it was confirmed to be associated with sex reversal in the tongue sole [64].

Interestingly, our data demonstrate that the distribution of these 3 types of genes in the black porgy genome is similar to that in Chinese ricefield eel (*Monopterus albus*), another teleost with natural sex-change from female to male (our unpublished results; Data of the *Monopterus* Whole Genome Shotgun project have been deposited at DDBJ/EMBL/GenBank under the accession number of AONE00000000). For example, 2 male-related genes (*piwil1 and piwil2*) are clustered together, while *lin28a* and *rspo1* are adjacent to each other. We also observed that most of these genes are congregated on the scaffolds 1, 2, 3, 11 and 15 (**Table 3**), suggesting the possible existence of a sex chromosome in the black porgy.

Conclusions

In summary, we sequenced and assembled the whole genome of Chinese black porgy. This is the first genomic report of Sparidae fish. Furthermore, we provided a genomic survey on the 26 genes potentially associated with sex change. The achieved genome data will be helpful for further biological and evolutionary studies. Furthermore, it will be valuable for implementation of molecular breeding, with support from our genomic data, to obtain genetic improvement of this economically important teleost fish.

Table 1. Summary of the achieved genome assembly and annotation.

**Genome assembly**	**Parameter**
contig N50 size (kb)	17.2
contig number (> 100 bp)	115,091
scaffold N50 size (Mb)	7.6
scaffold number (> 100 bp)	31,359
Total length (Mb)	688.1
Genome coverage (×)	257.6
The longest scaffold (bp)	22,574,836
Genome annotation	
Protein-coding gene number	19,465
Mean transcript length (kb)	17.3
Mean exons per gene	11.1
Mean exon length (bp)	170.2
Mean intron length (bp)	1519.2

Table 2. Detailed classification of repeat sequences in the assembled genome.

Type	Repbase TEs		TE proteins		*Denovo*		Combined TEs	
	Length (Mb)	In genome (%)	Length (Mb)	In genome (%)	Length (Mb)	In genome (%)	Length (Mb)	In genome (%)
DNA	20.930	3.041	2.200	0.320	58.340	8.479	68.130	9.902
LINE	10.240	1.488	6.950	1.010	26.760	3.889	33.020	4.789
SINE	1.120	0.163	2.340	0.000	3.780	0.550	4.550	0.661
LTR	7.200	1.046	35.410	0.340	25.980	3.062	31.270	4.544
Other	0.020	0.003	0.000	0.000	0.000	0.000	0.020	0.003
Unknown	0.000	0.000	0.000	0.000	25.370	3.687	25.370	3.687
Total	35.300	5.130	11.480	1.669	124.540	18.099	136.240	19.780

Table 3. Three types of genes potentially related to sex change in the black porgy genome

**Sex determination and differentiation genes**		
**Gene**	**Copy number**	**Scaffold**
fst	2	10, 17
Sox9	5	11, 13, 16, 19, 27
vasa	10	11, 14, 16, 20 , 27, 34, 37, 47, 53, 68
Beta-Catenin	4	2, 16, 64, 115
Piwil1	1	15
Piwil2	1	15
Sf-1	5	8, 15, 19, 21, 28
RSPO1	2	2, 74
foxl2	1	22
cyp19a1a	2	8, 28
gsdf	1	3
Figla	1	32
DMRT1	1	56
Wnt4	15	1, 2, 5, 6, 7, 8, 9, 18, 19, 20, 32, 34, 62 ,67, 122
Dax-1	1	14
Cyp11a1	3	2, 8, 33
Hsd3b1	2	7,36
amhr2	1	9
JNK1	12	1, 3, 4, 5, 7, 16, 17, 34, 38, 79, 117, 139
**Pluripotency factors**		
**Gene**	**Copy number**	**Scaffold**
Klf4	2	1, 142
nr5a2	6	8, 10, 15, 19, 21, 28
lin-28a	2	2, 3
oct4	1	3
**Apoptosis factors**		
**Gene**	**Copy number**	**Scaffold**
traf2-1	2	3, 15
caspase-2	1	2
tnfr1	1	2

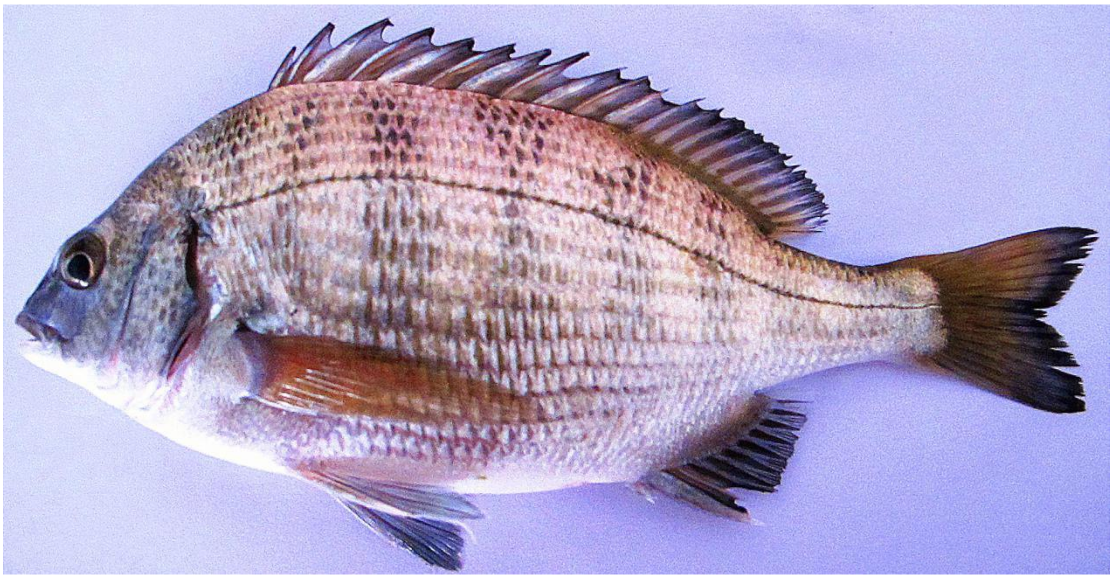

Figure 1. Image of a Chinese black porgy. It was captured from Laizhou Bay in Yantai, Shandong Province, China.

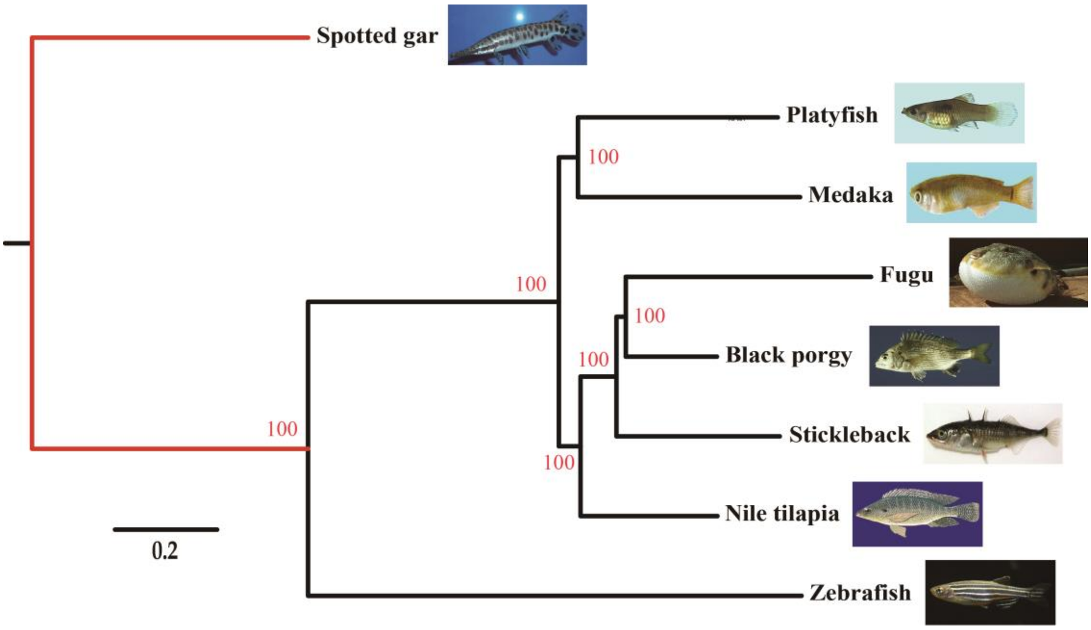

Figure 2. Phylogeny of ray-finned fishes. Spotted gar was used as the outgroup. These pictures in the phylogenetic tree were downloaded from the Fishbase.

Ethics approval and consent to participate

All animal experiments in this study were implemented in the light of the guidelines of the Animal Ethics Committee and ratified by the Institutional Review Board on

Bioethics and Biosafety of BGI, China

Availability of supporting data

The raw sequencing reads of all libraries and the transcriptome data have been deposited in the NCBI SRA database with accession numbers of SRA541936 and SRA587358. Supporting data are available in the GigaScience database, GigaDB.

Author's contributions

ZyZ, QS, and PX conceived the project. JX, CJ, JQ, FZ, HxL, HlL, DS, ZR and JC extracted the genomic DNA and performed genome sequencing. KZ, SC, ZwZ, XY, JZ, CB and JL assembled the genome and analyzed the data. TG, RG and JX participated discussions and provided valuable advice for revision. KZ, QS, ZyZ, PX, ZwZ and SC prepared the manuscript.

Acknowledgements

This work was supported by Aquatic Sanxin Engineering Major Project of Jiangsu Province (No. D2015-17), Key Research and Development (Modern Agriculture) Program of Jiangsu Province (No. BE2016326), Fund for Independent Innovation of Agricultural Science and Technology of Jiangsu Province (No. CX(17)2021), Aquatic Sanxin Engineering Project of Jiangsu Province (No.Y2016-23), Jiangsu Innovation Ability Construction Program (No. BM2015017), Nantong Applied Basic Research Program (No.MS12015071), Nantong Applied Basic Research Program (No. MS12015070 & MS12016029), and Zhenjiang Leading Talent Program for Innovation and Entrepreneurship.

Competing interests

The authors declare that they have no competing interests.

References

1. Gonzalez EB, Umino T, Nagasawa K. Stock enhancement programe for black sea bream, *Acanthopagrus schlegelii* (Bleeker), in Hiroshima Bay, Japan: a Review. Aquaculture Research 2008;**39**:1307-1315.

2. Zhang Y, Øverland M, Xie S et al. Mixtures of lupin and pea protein concentrates can efficiently replace high-quality fish meal in extruded diets for juvenile black sea bream (*Acanthopagrus schlegelii*). Aquaculture 2012;**354**:68-74.

3. Guo Z, Zhang W, Zhou Y et al. Feeding ratio and frequency affects cadmium bioaccumulation in black sea bream *Acanthopagrus schlegelii*. Aquaculture Environment Interactions 2015;**7**(2):135-145.

4. Murata O. Studies on the breeding of cultivated marine fishes. Bulletin of Fishery Laboratory, Kinki University 1998;**6**:1-101.

5. Kim YS, Biswas A, Ji SC et al. Phytase in soybean meal diet improves phosphorus availability of hybrid, female red sea bream *Pagrus major*× male black sea bream *Acanthopagrus schlegelii*. Aquaculture Science 2015;**63**(2):159-167.

6. Caporaso JG, Lauber CL, Walters WA et al. Ultra-high-throughput microbial community analysis on the Illumina HiSeq and MiSeq platforms. The ISME Journal 2012;**6**(8):1621-1624.

7. Li R, Yu C, Li Y et al. SOAP2: an improved ultrafast tool for short read alignment. Bioinformatics 2009;25(15):1966-1967.

8. Liu B, Shi Y, Yuan J et al. Estimation of genomic characteristics by analyzing k-mer frequency in denovo genome projects. Quantitative Biology 2013;**35**(s1-3):62-67.

9. Luo R, Liu B, Xie Y et al. SOAPdenovo2: an empirically improved memory-efficient short-read de novo assembler. Gigascience 2012;**1**:18.

10. Boetzer M, Henkel CV, Jansen HJ et al. Scaffolding pre-assembled contigs using SSPACE. Bioinformatics 2011;**27**:578-579.

11. Howe K, Clark M D, Torroja C F, et al. The zebrafish reference genome sequence and its relationship to the human genome. Nature 2013;**496**(7446):498.

12. Schartl M, Walter R B, Shen Y, et al. The genome of the platyfish, *Xiphophorus maculatus*, provides insights into evolutionary adaptation and several complex traits. Nature Genetics 2013;**45**(5):567-572.

13. Lee A P, Fan S, Philippe H, et al. The African coelacanth genome provides

insights into tetrapod evolution. Nature 2013;**7445** (496):311-316.

14. Chen S, Zhang G, Shao C, et al. Whole-genome sequence of a flatfish provides insights into ZW sex chromosome evolution and adaptation to a benthic lifestyle. Nature Genetics 2014;**46**(3):253-260
15. Venkatesh B, Lee A P, Ravi V, et al. Elephant shark genome provides unique insights into gnathostome evolution. Nature 2014,;**505**(7482):174.
16. Xu P, Zhang X, Wang X, et al. Genome sequence and genetic diversity of the common carp, *Cyprinus carpio*. Nature Genetics 2014;**46**(11):1212-1219.
17. Wang Y, Lu Y, Zhang Y et al. The draft genome of the grass carp (*Ctenopharyngodon idellus*) provides insights into its evolution and vegetarian adaptation. Nature Genetics 2015;**47**(6):625-631.
18. Lien S, Koop BF, Sandve SR et al. The Atlantic salmon genome provides insights into rediploidization. Nature 2016;**533**:200-205.
19. Lin Q, Fan S, Zhang Y et al. The seahorse genome and the evolution of its specialized morphology. Nature 2016;**540**(7633):395-399.
20. Yang J, Chen X, Bai J et al. The Sinocyclocheilus cavefish genome provides insights into cave adaptation. BMC Biology 2016;**14**(1):1.
21. Parra G, Bradnam K, Korf I. CEGMA: a pipeline to accurately annotate core genes in eukaryotic genomes. Bioinformatics 2007;**23**(9):1061-1067.
22. Simão FA, Waterhouse RM, Ioannidis P et al. BUSCO: assessing genome assembly and annotation completeness with single-copy orthologs. Bioinformatics 2015;**31**(19):3210-3212.
23. Tarailo-GraovacM, Chen N. Using RepeatMasker to identify repetitive elements in Genomic sequences. Current Protocols in Bioinformatics 2009;chapter 4: unit **4** 10.
24. Maziade M, Bouchard S, Gingras N et al. Long-term stability of diagnosis and symptom dimensions in a systematic sample of patients with onset of schizophrenia in childhood and early adolescence. II: Postnegative distinction and childhood predictors of adult outcome. The British Journal of Psychiatry 1996;**169**(3):371-378.

25. Mario S, Oliver K, Irfan G et al. AUGUSTUS: ab initio prediction of alternative transcripts. Nucleic Acids Research 2006;**34**:435-439.

26. Burge C, Karlin S. Prediction of complete gene structures in human genomic DNA. Journal of Molecular Biology 1997;**268**(1):78-94.

27. Birney E, Clamp M, Durbin R. GeneWise and Genomewise. Genome Research 2004;**14**(5):988-995.

28. Trapnell C, Pachter L, Salzberg SL. TopHat: discovering splice junctions with RNA-Seq. Bioinformatics 2009;**25**(9):1105-1111.

29. Trapnell C, Williams BA, Pertea G et al. Transcript assembly and quantification by RNA-Seq reveals unannotated transcripts and isoform switching during cell differentiation. Nature Biotechnology 2010;**28**(5):511-515.

30. Elsik CG, Mackey AJ, Reese JT et al. Creating a honey bee consensus gene set. Genome Biology 2007;**8**(1):90-105.

31. Finn RD. Pfam: the protein families database. Nucleic Acids Research 2014;**42**(Database issue):D222-230.

32. Attwood TK. The PRINTS database: A resource for identification of protein families. Briefings in Bioinformatics 2002;**3**(3):252-263.

33. Bru C, Courcelle E, Beausse Y et al. The ProDom database of protein domain families: more emphasis on 3D. Nucleic Acids Research 2005;**33**(Database issue):212-215.

34. Letunic I, Copley RR, Schmidt S et al. SMART 4.0: towards genomic data integration. Nucleic Acids Research 2004;**32**(Databaseissue):D142-D144.

35. Boeckmann B, Bairoch A, Apweiler R et al. The Swiss-Prot knowledgebase and its supplement TREMBL in 2003. Nucleic Acids Research 2003;**31**(1):365-370.

36. Hunter S, Apweiler R, Attwood TK et al. InterPro: the integrative protein signature database. Nucleic Acids Research 2009;**37**(suppl 1):D211-D215.

37. Hingamp P, Broek AEVD, Stoesser G et al. The EMBL nucleotide sequence database. Molecular Biotechnology 1999;**12**(3):255-267.

38. Kanehisa M, Goto S. KEGG: kyoto encyclopedia of genes and genomes. Nucleic Acids Research 2000;**27**(1):29-34.

39. Cunningham F, Amode MR, Barrell D et al. Ensembl 2015. Nucleic Acids Research 2014;**43**(Database issue):D662-629.

40. Li L, Stoeckert CJ, Roos DS. OrthoMCL: identification of ortholog groups for eukaryotic genomes. Genome Research 2003;**13**(9):2178-2189.

41. Edgar RC. MUSCLE: multiple sequence alignment with high accuracy and high throughput. Nucleic Acids Research 2004;**32**(5):1792-1797.

42. Guindon S, Dufayard JF, Lefort V et al. New algorithms and methods to estimate maximum-likelihood phylogenies: assessing the performance of PhyML 3.0. Systematic Biology 2010;**59**(3):307-321.

43. Aparicio S, Chapman J, Stupka E et al. Whole-genome shotgun assembly and analysis of the genome of Fugu rubripes. Science 2002;**297**(5585):1301-1310.

44. Wu GC, Chang CF. The switch of secondary sex determination in protandrous black porgy, *Acanthopagrus schlegeli*. Fish Physiology and Biochemistry 2013;**39**(1):33-38.

45. Xiao YM, Chen L, Liu J et al. Contrast expression patterns of *JNK1* during sex reversal of the rice-field eel. Journal of Experimental Zoology Part B: Molecular and Developmental Evolution 2010;**314**(3):242-256.

46. Webster KA, Schach U, Ordaz A et al. *Dmrt1* is necessary for male sexual development in Zebrafish. Developmental Biology 2017;**422**(1):33-46.

47. Mount DW. Using the basic local alignment search tool (blast). Cold Spring Harbor Protocols 2007;**2007**(7):pdb.top17.

48. Nanda I, Kondo M, Hornung U, et al. A duplicated copy of DMRT1 in the sex-determining region of the Y chromosome of the medaka, *Oryzias latipes*. Proceedings of the National Academy of Sciences 2002; **99**(18):11778-11783.

49. Masuyama H, Yamada M, Kamei Y, et al. Dmrt1 mutation causes a male-to-female sex reversal after the sex determination by Dmy in the medaka. Chromosome Research 2012;**20**(1):163-176.

50. Wu GC, Du JL, Lee YH et al. Current status of genetic and endocrine factors in the sex change of protandrous black porgy, *Acanthopagrus schlegeli* (Teleostean). Annals of the New York Academy of Sciences 2005;**1040**(1):206-214.

51. Wu G C, Chiu P C, Lin C J et al. Testicular dmrt1 is involved in the sexual fate of the ovotestis in the protandrous black porgy. Biology of Reproduction 2012; **86**(2).

52. Chen S, Zhang G, Shao C et al. Whole-genome sequence of a flatfish provides insights into ZW sex chromosome evolution and adaptation to a benthic lifestyle. Nature Genetics 2014;**46**(3):253-260.

53. Cui Z, Liu Y, Wang W et al. Genome editing reveals dmrt1 as an essential male sex-determining gene in Chinese tongue sole (*Cynoglossus semilaevis*). Scientific Reports 2017;**7**:42213.

54. Wu G C, Tomy S, Nakamura M et al. Dual roles of cyp19a1a in gonadal sex differentiation and development in the protandrous black porgy, *Acanthopagrus schlegeli*. Biology of Reproduction 2008;**79**(6):1111-1120.

55. Dong X, Chen S, Ji X et al. Molecular cloning, characterization and expression analysis of Sox9a and Foxl2 genes in half-smooth tongue sole (*Cynoglossus semilaevis*). Acta Oceanologica Sinica 2011;**30**(1):68-77.

56. Li H, Xu W, Zhang N et al. Two Figla homologues have disparate functions during sex differentiation in half-smooth tongue sole (*Cynoglossus semilaevis*). Scientific Reports 2016;**6**:28219.

57. Rodriguez-Mari A, Yan YL, Bremiller RA et al. Characterization and expression pattern of zebrafish Anti-Mu¨llerian hormone (Amh) relative to sox9a, sox9b, and cyp19a1a, during gonad development. Gene Expression Patterns 2005;**5**:655–667.

58. Raghuveer K, Garhwal R, Wang DS et al. Effect of methyl testosterone-and ethynyl estradiol-induced sex differentiation on catfish, *Clarias gariepinus*: expression profiles of DMRT1, Cytochrome P450aromatases and 3 beta-hydroxysteroid dehydrogenase. Fish Physiology and Biochemistry 2005;**31**(2):143-147.

59. Xie QP, He X, Sui YN et al. Haploinsufficiency of SF-1 Causes Female to Male Sex Reversal in Nile Tilapia, *Oreochromis niloticus*. Endocrinology 2016;**157**(6):2500-2514.

60. Ye D, Lv D, Song P et al. Cloning and characterization of a rice field eel vasa-like gene cDNA and its expression in gonads during natural sex transformation. Biochemical Genetics 2007;**45**(3-4):211-224.
61. Xiao YM, Chen L, Liu J et al. Contrast expression patterns of JNK1 during sex reversal of the rice field eel. Journal of Experimental Zoology Part B 2010;**314**(3):242-256.
62. Böhne A, Wilson CA, Postlethwait JH et al. Variations on a theme:Genomics of sex determination in the cichlid fish *Astatotilapia burtoni*. BMC Genomics 2016;**17**(1):883.
63. Bernard P, Harley V. Wnt4 action in gonadal development and sex determination. The International Journal of Biochemistry & Cell Biology 2007;**39**(1):31–43.
64. Hu Q, Zhu Y, Liu Y et al. Cloning and characterization of wnt4a gene and evidence for positive selection in half-smooth tongue sole (*Cynoglossus semilaevis*). Scientific Reports 2014;**4**:7167.
65. Matsui T, Raya Á, Kawakami Y et al. Noncanonical Wnt signaling regulates midline convergence of organ primordia during zebrafish development. Genes & Development 2005;**19**(1):164-175.
66. Nicol B, Guerin A, Fostier A, et al. Ovary-predominant wnt4 expression during gonadal differentiation is not conserved in the rainbow trout (*Oncorhynchus mykiss*). Molecular Reproduction and Development 2012;**79**(1):51-63.

Click here to access/download
Supplementary Material
response to reviewers .doc
