# Supplementary material for: Draft genome of the protandrous Chinese black porgy, Acanthopagrus schlegelii
Source: Gigascience. 2018 Feb 26;7(4):giy012. doi: 10.1093/gigascience/giy012 (PMC5893958; doi:10.1093/gigascience/giy012)
Supplement: GIGA-D-17-00137_Revision-2.pdf [file giy012_giga-d-17-00137_revision-2.pdf]

GigaScience

Draft Genome of the Protandrous Chinese Black Porgy, Acanthopagrus schlegelii

--Manuscript Draft--

Manuscript Number:	GIGA-D-17-00137R2
Full Title:	Draft Genome of the Protandrous Chinese Black Porgy, Acanthopagrus schlegelii
Article Type:	Data Note
Funding Information:	Aquatic Sanxin Engineering Major Project of Jiangsu Province (D2015-17) — Dr. Zhiyong Zhang<br>Jiangsu Innovation Ability Construction Program (BM2015017) — Dr. Zhiyong Zhang<br>Nantong Applied Basic Research Program (MS12015071) — Dr. Zhiwei Zhang<br>Key Research and Development (Modern Agriculture) Program of Jiangsu Province (BE2016326) — Dr. Zhiwei Zhang<br>Aquatic Sanxin Engineering Project of Jiangsu Province (Y2016-23) — Dr. Zhiyong Zhang<br>Nantong Applied Basic Research Program (MS12015070 & MS12016029) — Dr. Zhiyong Zhang<br>Zhenjiang Leading Talent Program for Innovation and Entrepreneurship — Dr. Qiong Shi
Abstract:	Background: As one of the most popular and valuable commercial marine fishes in China and East Asian countries, the Chinese black porgy (Acanthopagrus schlegelii) also known as the blackhead seabream, has some attractive characteristics, such as fast growth rate, good meat quality, resistance to diseases and excellent adaptability to various environments. Furthermore, the black porgy is a good model for investigating sex changes in fish due to its protandrous hermaphrodite. Here, we obtained a high-quality genome assembly of this interesting teleost species and performed a genomic survey on potential genes associated with the sex-change phenomenon.<br>Findings: We generated 175.4 gigabases (Gb) of clean sequence reads using a whole-genome shotgun sequencing strategy. The final genome assembly is approximately 688.1 megabases (Mb), accounting for 93% of the estimated genome size (739.6 Mb). The achieved scaffold N50 is 7.6 Mb, reaching a relatively high level among sequenced fish species. Meanwhile, we identified 19,465 protein-coding genes, which had an average transcript length of 17.3 kb. By performing a comparative genomic analysis, we found three types of genes potentially associated with sex change, which are useful for the prediction of related genetic basis for the interesting protandrous hermaphrodite.<br>Conclusions: We provided a draft genome assembly of the Chinese black porgy and discussed about the potential genetic mechanisms of sex change. These data are also an important resource for studying the biology and facilitating the molecular breeding of this economically important fish.
Corresponding Author:	Qiong Shi, PhD<br>BGI<br>Shenzhen, CHINA
Corresponding Author Secondary Information:	
Corresponding Author's Institution:	BGI
Corresponding Author's Secondary Institution:	
First Author:	Qiong Shi, PhD
First Author Secondary Information:	
Order of Authors:	Qiong Shi, PhD

Powered by Editorial Manager® and ProduXion Manager® from Aries Systems Corporation

	Zhiyong Zhang, PhD
	Kai Zhang, PhD
	Shuyin Chen, PhD
	Zhiwei Zhang, PhD
	Xinxin You, PhD
	Jinyong Zhang, PhD
	Chao Bian, PhD
	Jin Xu
	Chaofeng Jia
	Jun Qiang
	Fei Zhu
	Hongxia Li
	Hailin Liu
	Dehua Shen
	Zhonghong Ren
	Jieming Chen
	Jia Li
	Tianheng Gao
	Ruobo Gu
	Junmin Xu
	Pao Xu, PhD
**Order of Authors Secondary Information:**	
**Response to Reviewers:**	Dear Editor,<br>Thanks for your kind help. We also appreciate the instructive comments from the two reviewers. According to their suggestions, we performed additional analyses and made a careful revision on our previous manuscript. Our point-by-point responses are attached for your consideration.<br><br>Please also include the details on your BUSCO analysis in the manuscript (and we will also ask you to upload BUSCO results to GigaDB). Why was Busco v2 used, when the latest version is v3?<br><br>Answer: Thanks for your good advice. We also ran BUSCO v3 on the genome assembly, and the final BUSCO score was 89.1% (C:89.1% [S:86.2%, D:2.9%], F:2.5%, M:8.4%, n:4584). For the predicted gene set, the new BUSCO score reached 85.5%, (C:85.5% [S:82.3%, D:3.2%], F:2.8%, M:11.7%, n:4584). Please find more details about these revisions on lines 118-121 and 148-150 in the revised manuscript. In addition, we uploaded corresponding BUSCO results to the GigaDB.<br><br>Highlighting revisions to the manuscript in color can help us and the reviewers to quickly assess your next version.<br><br>Answer: Yes, it’s done. We highlighted all the revisions in yellow for your convenience.<br><br>Finally, please note that GigaScience allows a maximum number of 3 indications of co-first authorship and a maximum number of 2 co-corresponding authors. Please revise accordingly.<br><br>Answer: Thanks for your comments. We revised the authorship information in accordance with your instructions. Three co-first authors (Prof. Zhang, Mr. Zhang and

Dr. Chen) and two co-corresponding authors (Profs. Xu and Shi) are marked in the revised manuscript.

Best regards,
Qiong Shi, PhD, Professor
BGI
Shenzhen 518083
China

P.S. point-by-point responses to the reviewers' comments.

Reviewer #1: I thank the authors for addressing my comments in a satisfactory fashion. I have a couple of comments, more regarding choice of words.
Lines 111-112, and elsewhere in the manuscript: I am unsure if I would have chosen to discuss the N50 length of the scaffolds as "reaching a relatively high level among sequenced fish species". Do you mean length instead of level?

Answer: Thanks for your question and advice. You are right. We changed "level" to "length" on line 110 of the revised manuscript.

While the manuscript discusses the manner of whole-genome duplication well, I found the wording in the answer a bit strange: "Black porgy is a diploid species, hence multiple copies of wnt4, vasa and JNK1 in the genome assembly may be resulted from the teleost-specific whole genome duplication." Most gene assemblers output a haploid assembly, based on a diploid genome, but sometimes both haplotypes are output. Do you mean that this has happened in this case? Or that the multiple copies have been retained since the whole-genome duplication in the ancestor to the teleosts?

Answer: Thanks for your good questions. Yes, you are right. The multiple copies may be retained since the whole-genome duplication in the ancestor to the teleost. Please find corresponding changes on lines 222-223 in the revised manuscript.

Reviewer #2: The revised version has been improved, but several of the reviewer comments have not been addressed appropriately. I therefore encourage the authors to take the following points into full consideration when revising the current version of the study:good questions
1. Comparison to other fish genome assemblies: I think that the assembly should only be compared to other teleost fishes. Elephant shark (cartilaginous fish) and coelacanth (lobe-finned fish), although being 'fish' in the broader sense, are no more justifiable to compare to than tetrapods.

Answer: Thanks for your comments and advice. We removed elephant shark and coelacanth in the revised manuscript. Please find more details on lines 111-114 in the revised manuscript.

2. BUSCO analysis: You mention that you used the BUSCOv2 actinopterygii dataset in the response to the reviewers, but this information needs to be included in the manuscript as well.

Answer: Thanks for your advice. According to the reviewer one's opinions, we updated BUSCO v2 to v3, although the final results are similar. We hence added the updated information to the revised manuscript (on lines 118-121 and 148-150 of the revised manuscript).

3. Phylogenetic analysis:
3.1 The phylogenetic tree obtained with MrBayes should be included as a supplementary figure.

Answer: Thanks for your advice. However, please be informed that a Data Note paper do not provide space for any supplementary document. We rebuilt the phylogenetic tree using MrBayes (Version 3.2, with the GTR+gamma model), which is the same in topology as our Figure 2 in the main content.

Powered by Editorial Manager® and ProduXion Manager® from Aries Systems Corporation

3.2 In the response to the reviewers you mention that you removed the statement of a 'close relationship' of black porgy to fugu, but it is still in the manuscript (l. 174).

Answer: Sorry for the mistake. We removed it now in the new manuscript. Please see more details on lines 172-173 of the revised manuscript.

3.3 Furthermore, in the revised version it is newly stated that the phylogenetic tree 'suggests a lower neutral evolutionary rate than any other investigated teleost' l. 175). It is unclear how this statement is substantiated by the data presented. Is this referring to the branch lengths in the phylogenetic tree? Porgy and tilapia do not seem to be very different. The conclusion of a specifically slow rate of molecular evolution in black porgy has to be supported with a statistical tests such as relative rate and two-cluster tests.

Answer: Thanks for the nice comments and advice. Yes, you are right. We hence removed the conclusions due to the lack of sufficient evidence in the revised manuscript. Please see more details on lines 172-173.

4. Survey of sex related genes: This part still needs major improvement.
4.1. First of all, it still remains unresolved why there are multiple copies of many of these genes in the genome assembly. In the response to reviewer 1, it is stated that '… hence multiple copies of wnt4, vasa and JNK1 in the genome assembly may be resulted from the teleost-specific whole genome duplication.'; in response to reviewer 2: 'Several genes, such as Wnt4, vasa and JNK1, with multiple copies in the assembly may be copy number variants.' As mentioned in the previous reviews, this really needs to be addressed in more detail. For example, the table states that there are 15 copies of wnt4 in porgy. As the authors correctly state, there is wnt4a and wnt4b in vertebrates, as well as potential additional paralogs from the teleost genome duplication etc. All genes reported here need to be analyzed by phylogenetic methods to appropriately establish their orthology to other fishes and vertebrates. While I appreciate that the authors are in the process of cloning these individual copies, their sequences from the genome assembly need be submitted to NCBI and/or provided as supplements as part of the current article.

Answer: Thanks for your nice comments and advice. Sorry for the misleading statements. In fact, the multiple copies of wnt4, vasa and jnk1 in the genome assembly may be retained since the whole-genome duplication in the ancestor to the teleost. Please find more details of the corrections on lines 222-249 of the revised manuscript. According to your advice, we employed all the predicted protein sequences to rebuild a new phylogenetic tree using PhyML, and we still observed that they were clustered with each corresponding homologue from other vertebrates (data not shown). Please find corresponding changes on lines 186-188 of the revised manuscript.
We also uploaded these data, including the ML tree and the predicted coding sequences and protein sequences of these genes, to the GigaDB.

4.2 Secondly, as mentioned in my previous report, the reasoning for the existence of a putative sex chromosome in black porgy needs to be supported beyond the fact that some of the sex-related genes are located on the same scaffold. Is the linkage of these specific genes in the black genome assembly unique or are these general blocks of conserved synteny across teleosts? The authors state: "our data demonstrate that the distribution of these 3 types of genes in the black porgy genome is similar to that in Chinese ricefield eel" but give no further explanation in which ways the porgy is similar to the rice eel. Again, if this refers to linkage of specific genes, it would need to be demonstrated that the linkage of these genes is unique to both porgy and ricefield eel. In their response to the reviewer, the authors further mention that the data are similar to arowana, which has identified sex chromosomes, but give no further explanation in which ways porgy and arowana are similar to support the conclusion that a sex chromosome might be present in porgy. Without further details, such conclusion remains unsubstantiated and should be removed.

Answer: Thanks for your nice comments and advice. The linkage of these specific genes in the black genome assembly seems to be unique. However, we don't have solid evidence to support existence of a sex chromosome in the porgy. We hence

Powered by Editorial Manager® and ProduXion Manager® from Aries Systems Corporation

	removed these conclusions in the revised manuscript (on lines 243-249). Meanwhile, more details about the similarity to ricefield eel were provided on lines 246-248 in the revised manuscript.<br><br>5. It would be better to mention the information on medaka dmrt1 after the statement on tongue sole dmrt1 (l. 198).<br><br>Answer: Thanks for your advice. It’s done on lines 200-202 of the revised manuscript.<br><br>6. Gene duplicates should be called paralogs, not 'isotypes'.<br><br>Answer: Sorry for our mistake. We corrected it on line 217 in the revised manuscript<br><br>7. Citations in the later part of the main text seem to out of order (starting at about citation 48 and following). Please double-check that the citations in the text and the bibliography correspond to each other.<br><br>Answer: Thanks for your careful checking. We corrected the citation orders in the revised manuscript.<br><br>8. Throughout the manuscript and tables, again, please follow the gene nomenclature conventions for teleost fish (see https://wiki.zfin.org/display/general/ZFIN+Zebrafish+Nomenclature+Guidelines).<br><br>Answer: Thanks for your comments. It’s done for all the gene names.
**Additional Information:**	
**Question**	**Response**
Are you submitting this manuscript to a special series or article collection?	No
**Experimental design and statistics**<br><br>Full details of the experimental design and statistical methods used should be given in the Methods section, as detailed in our Minimum Standards Reporting Checklist. Information essential to interpreting the data presented should be made available in the figure legends.<br><br>Have you included all the information requested in your manuscript?	Yes
**Resources**<br><br>A description of all resources used, including antibodies, cell lines, animals and software tools, with enough information to allow them to be uniquely identified, should be included in the Methods section. Authors are strongly encouraged to cite Research Resource Identifiers (RRIDs) for antibodies, model organisms and tools, where possible.<br><br>Have you included the information requested as detailed in our Minimum	Yes

Standards Reporting Checklist?	
**Availability of data and materials**<br><br>All datasets and code on which the conclusions of the paper rely must be either included in your submission or deposited in publicly available repositories (where available and ethically appropriate), referencing such data using a unique identifier in the references and in the “Availability of Data and Materials” section of your manuscript.<br><br>Have you have met the above requirement as detailed in our Minimum Standards Reporting Checklist?	Yes

Manuscript Click here to download Manuscript manuscript-revised.docx

Draft Genome of the Protandrous Chinese Black Porgy, *Acanthopagrus schlegelii*

Zhiyong Zhang[1]†, Kai Zhang[2,3,4]†, Shuyin Chen[1]†, Zhiwei Zhang[1], Jinyong Zhang[5], Xinxin You[3], Chao Bian[3,6], Jin Xu[1], Chaofeng Jia[1], Jun Qiang[2], Fei Zhu[1], Hongxia Li[2], Hailin Liu[1], Dehua Shen[1], Zhonghong Ren[1], Jieming Chen[3], Jia Li[3], Tianheng Gao[7], Ruobo Gu[3,6], Junmin Xu[3,6], Qiong Shi[3,4,6]*, Pao Xu[2]*

1 Jiangsu Marine Fishery Research Institute, Nantong, Jiangsu 226007, China

2 Freshwater Fishery Research Center, Chinese Academy of Fishery Sciences, Wuxi, Jiangsu 214081, China

3 Shenzhen Key Lab of Marine Genomics, Guangdong Provincial Key Lab of Molecular Breeding in Marine Economic Animals, BGI Academy of Marine Sciences, BGI Marine, BGI, Shenzhen 518083, China

4 BGI Education Center, University of Chinese Academy of Sciences, Shenzhen, Guangdong 518083, China

5 State Key Laboratory of Freshwater Ecology and Biotechnology, Institute of Hydrobiology, Chinese Academy of Sciences, Wuhan, Hubei 430000, China

6 BGI-Zhenjiang Institute of Hydrobiology, Zhenjiang, Jiangsu 212000, China

7 College of Oceanography, Hohai University, Nanjing, Jiangsu 210098, China

* Correspondence address. Pao Xu, Freshwater Fishery Research Center, Chinese Academy of Fishery Sciences, Wuxi, Jiangsu 214081, China (tel: +86-138 0619 0669; email: xup@ffrc.cn) ; Qiong Shi, Shenzhen Key Lab of Marine Genomics, Guangdong Provincial Key Lab of Molecular Breeding in Marine Economic Animals, BGI Academy of Marine Sciences, BGI Marine, BGI, Shenzhen 518083, China (tel: +86-185 6627 9826; email: shiqiong@genomics.cn)

† Contributed equally to this work.

Abstract

Background: As one of the most popular and valuable commercial marine fishes in China and East Asian countries, the Chinese black porgy (*Acanthopagrus schlegelii*) also known as the blackhead seabream, has some attractive characteristics, such as fast growth rate, good meat quality, resistance to diseases and excellent adaptability to various environments. Furthermore, the black porgy is a good model for investigating sex changes in fish due to its protandrous hermaphrodite. Here, we obtained a high-quality genome assembly of this interesting teleost species and performed a genomic survey on potential genes associated with the sex-change phenomenon.

Findings: We generated 175.4 gigabases (Gb) of clean sequence reads using a whole-genome shotgun sequencing strategy. The final genome assembly is approximately 688.1 megabases (Mb), accounting for 93% of the estimated genome size (739.6 Mb). The achieved scaffold N50 is 7.6 Mb, reaching a relatively high level among sequenced fish species. Meanwhile, we identified 19,465 protein-coding genes, which had an average transcript length of 17.3 kb. By performing a comparative genomic analysis, we found three types of genes potentially associated with sex change, which are useful for the prediction of related genetic basis for the interesting protandrous hermaphrodite.

Conclusions: We provided a draft genome assembly of the Chinese black porgy and discussed about the potential genetic mechanisms of sex change. These data are also an important resource for studying the biology and facilitating the molecular breeding of this economically important fish.

Keywords: Chinese black porgy; *Acanthopagrus schlegelii*; whole genome sequencing; genome assembly; sex-change related genes

Data description

Background information

As one of the most popular and valuable commercial marine fishes in China and East

Asian countries, the Chinese black porgy (*Acanthopagrus schlegelii*), also known as the blackhead seabream, has some interesting characteristics, such as fast growth rate, good meat quality, resistance to diseases and good adaptability to various environments. It is often farmed for food in the South China Sea and the coastal waters around Japan and Korea [1,2]. In addition, it is an eurythermal and euryhaline fish, living in a wide range of water temperatures and salinities. Recently, some basic studies on the genetic improvement for its growth and disease resistance have been increasingly performed in order to increase efficiency of farming [3].

The Chinese black porgy is also a good model for investigating the genetic mechanisms of sex change due to its interesting life cycle. It is a functional male during the first 2 years and a subsequent female during the next couple of years. Recently, a good hybrid of the Japanese seabream (*Pagrosomus major*; ♀) and the Chinese black porgy (♂) has become available [4,5], with better growth performance and higher tolerance against low temperature than its parents. However, related genetic mechanisms for these interesting biological characteristics are still unclear. Here, we sequenced and assembled the whole genome of the Chinese black porgy, before performing a genomic survey on potential genes associated with the sex-change phenomenon.

Sample and Sequencing

The wild black porgy (NCBI Taxonomy ID: 72011; Fishbase ID: 6531) individuals (**Figure 1**) were collected from Laizhou Bay in Yantai, Shandong Province, China. Genomic DNA was extracted from the muscle of a female fish using Qiagen GenomicTip100 (Qiagen, Hilden, USA). We employed the whole-genome shotgun sequencing strategy and constructed the subsequent three short-insert libraries (250-bp, 500-bp and 800-bp) and four long-insert libraries (2-kb, 5-kb, 10-kb and 20-kb) in accordance with the standard protocol from Illumina (San Diego, USA). All these constructed libraries were sequenced on the Illumina HiSeq 2000 system [6] (the read length is 125 bp). Finally, we generated a total of 272.9-Gb raw reads from all seven libraries.

Before assembly of the sequencing reads, SOAPfilter v2.2 software [7] with default parameters (-y -p -g 1 -o clean -M 2 -f 0) was utilized to remove low-quality raw reads (including reads with 10 or more non-sequenced/low-quality bases), PCR duplicates and adaptor sequences. Subsequently, we obtained approximately 175.4 Gb of clean reads for further genome size prediction and assembling. A *k*-mer analysis with the formula G = *k*_num/*k*_depth [8] was performed to estimate the genome size of Chinese black porgy. In our current study, the achieved total number of *k*-mers and *k*_depth was 2.81×10^{10} and 38, respectively. Therefore, the genome size of Chinese black porgy is estimated to be 739.6 Mb. Based on this result, the retained reads were calculated to cover approximately 238-fold of the whole genome.

Assembly and Evaluation

To obtain a genome assembly, we employed the SOAPdenovo2 v2.04.4 [9] with optimized parameters (pre-graph -K 27 -p 16 -d 1; contig –M 3; scaff -F -b 1.5 -p 16) using these clean reads. In brief, the reads from short-insert libraries were applied for the contig assembly, before alignment of all the filtered reads onto the contigs for linking these contigs to generate scaffolds. GapCloser v1.12 software [7] with default parameters was subsequently used to fill some intra-scaffold gaps in the local assembly, in which the reads were equipped with one end uniquely mapped to a contig and the other end located within a gap. Meanwhile, SSPACE (version 2.0) [10] with default parameters was employed to obtain super scaffolds with the reads from the long-insert libraries (2-kb, 5-kb, 10-kb and 20-kb). The final genome assembly was approximately 688.1 Mb, which accounts for 93.0% of the estimated genome size (739.6 Mb; **Table 1**).

The achieved scaffold N50 is 7.64 Mb, reaching a relatively high length among sequenced fish species. In comparison, other scaffolds have levels of 1.55 Mb for the zebrafish [11], 1.1 Mb for platy fish [12], 867 kb for half-smooth tongue sole [13], 1 Mb for common carp [14], 6.4 Mb for grass carp [15], 2.97 Mb for Atlantic salmon [16], 1.8 Mb for a seahorse [17] and 1.15 Mb for a Chinese barbel fish [18]. Core Eukaryotic Genes Mapping Approach (CEGMA; version 2.5) [19] with a set of 248

conserved Core Eukaryotic Genes (CEGs) was employed to assess the completeness of the final assembly. The estimates suggest that 90.7% CEGs are complete and 92.3% are partial. Meanwhile, Benchmarking Universal Single-Copy Orthologs (BUSCO; version 3) [20] was applied to evaluate the quality of the generated genome assembly. The final BUSCO score reached 89.1%, (C:89.1% [S:86.2%, D:2.9%], F:2.5%, M:8.4%, n:4584). These results from CEGMA and BUSCO suggested that the assembled genome covers the majority of the gene space.

Annotation

We used RepeatProteinMask (version 4.0.6) [21] in RepeatMasker to identify the repetitive sequences, before employing RepeatModeller (version 1.05) [22] and LTR_FINDER.x86_64-1.0.6 to construct a *de novo* repeat library. Additionally, repetitive elements were predicted using Tandem Repeat Finder (TRF, version 4.04). Finally, we observed that the identified repeat sequences cover 19.78% of the assembled genome (**Table 2**).

Prediction of protein-coding genes was performed based on the integration of *ab initio* prediction, homologue prediction and transcriptome-based prediction. The *ab initio* prediction was carried out with Augustus (version2.5) [23] and GENSCAN (version1.0) [24] on the repeat-masked assembly. For the homology-based gene prediction, homologous proteins of several reported fishes (zebrafish, Japanese puffer, stickleback and medaka) were downloaded from Ensembl release 75 and aligned to the assembled genome using tBlastn (version2.2.19) with e-value $\leq 1e^{-5}$. Subsequently, all the achieved alignments were analyzed using Genewise (version2.2.0) software [25] to search for precise gene structures. We further filtered out these short (less than 150 bp), prematurely terminated or frame-shifted genes. For the transcriptome-based prediction, we obtained transcriptome data from a mixture of liver, muscle, skin, gill and brain of a female fish at cDNA level. Those with low-quality bases, adapter sequences and duplicated sequences were removed and we acquired approximately 8 Gb of high-quality clean reads. Subsequently, TopHat2.1.1 [26] and Cufflinks (version 2.2.1) [27] were applied to predict gene structures using

these retained reads. Eventually, the three gene sets generated from the prediction approaches were integrated into a comprehensive and non-redundant gene set using GLEAN [28]. As summarized in **Table 1**, the final gene set contains 19,465 genes, with an average transcript length of 17.3 kb. In addition, we ran BUSCO v3 [20] on the predicted coding sequences (CDS), and the final BUSCO score was up to 85.5%, (C:85.5% [S:82.3%, D:3.2%], F:2.8%, M:11.7%, n:4584). Simultaneously, all the protein sequences from the GLEAN analysis were mapped onto several public databases, including Pfam [29], PRINTS [30], ProDom [31] and SMART [32], to detect the known motifs and domains within our genome assembly. The data demonstrated that 99.3% of the predicted genes from the assembled genome contain at least one related functional assignment from other public databases, including Swiss-Prot [33], Interpro [34], TrEMBL [35] and KEGG [36].

Phylogenetic Analysis

In order to examine the phylogenetic position of the Chinese black porgy, we downloaded protein sequences of seven reported fishes, including spotted gar (*Lepisosteus oculatus*), stickleback (*Gasterosteus aculeatus*), Japanses fugu (*Takifugu rubripes*), medaka (*Oryzias latipes*), zebrafish (*Danio rerio*), platyfish (*Xiphophorus maculatus*), and Nile tilapia (*Oreochromis niloticus*) from Ensembl (release 83) [37]. These sequences were used to construct gene families by OrthoMCL [38] and eventually generated a total of 17,431 gene families by the all-to-all BLASTP strategy with an E-value of 1e^{-5}. In additional, 65 gene families were only presented in the black porgy genome.

Subsequently, 3,239 single-copy orthologous genes from these gene families were selected. These single-copy genes were further aligned using MUSCLE (version 3.8.31) with default parameters [39], before the protein alignments were changed to corresponding CDS using an in-house perl script. All these nucleotide sequences of each species were integrated into a supergene, which were used to build a phylogenetic tree with PhyML [40]. Our final data orientated the phylogenetic position of the black porgy in teleost (**Figure 2**).

Analysis of Three Types of Genes for Sex Change

Sex change (secondary sex determination) is a universal phenomenon in fish, but it usually does not occur in amphibians and mammals. The black porgy is a good model for the study on the molecular mechanisms of sex change. For providing a genomic survey on these genes in the assembled genome, protein sequences of three main types of genes potentially associated with sex change, including sex determination and differentiation genes, pluripotency factors and apoptosis factors [41–43], were downloaded from the NCBI database and used for homology searches against the black porgy genome with tBlastn (version2.2.19) [44]. We chose alignments with coverage > 70% and identity > 70% for further prediction of gene structures using Genewise (version 2.2.0) [25]. Finally, we obtained homologous sequences of 26 genes in the genome assembly of Chinese black porgy (see more details in **Table 3**). All these predicted protein sequences were employed to build a phylogenetic tree using PhyML [40], and we eventually observed that they were clustered with each corresponding homologue from other vertebrates.

Previous studies have revealed that multiple genes, including *dmrt1*, *cyp19a1a*, *wnt4*, *sox9*, *sf1*, *foxl2*, *figla*, *amhr2* and *dax1*, are associated with sex change in the black porgy [41,45-47]. These sex determination and differentiation genes were also identified in our assembled scaffolds (in the first batch of **Table 3**). In the current study, the important male-related *dmrt1* and the steroidogenesis-suppressing factor *dax1* were mapped on the scaffolds 56 and 14 of the black porgy genome, respectively.

It was reported that *dmrt1* may play a key role in the sex change of the black porgy, while the male-phase maintenance of male development was regulated by the brain–pituitary–gonadal axis via the GnRH-GtH-Dmrt1 pathway [41]. In the economically important half-smooth tongue sole (*Cynoglossus semilaevis*), *dmrt1* has been proven to be a necessary male sex-determining gene [48,49]. Moreover, previous findings suggest that a duplicate of *dmrt1* is the male sex determinant in medaka and *dmrt1* mutation causes a male–female sex reversal [50,51]. We also validated the existence

of *foxl2* and *cyp19a1a*, two putative female-related genes, in the black porgy genome. Previous findings revealed that *cyp19a1a* plays dual roles in gonadal development, while both *cyp19a1a* and *foxl2* are related to the sex change of the black porgy [47]. However, *foxl2* has functions in sex differentiation, but it is not essential for sex determination and sex change in the tongue sole [52].

figla, with only one copy in the black porgy, is a germ-cell-specific transcription factor related to ovary development and differentiation [53]. However, two isotypes (*figla_tv1* and *figla_tv2*) were reported in the tongue sole. *figla_tv1* possesses a conserved function in folliculogenesis as found in other vertebrates, while *figla_tv2* may play a role in the spermatogenesis of pseudo-males by regulating the synthesis and metabolism of steroid hormones [53]. *sf1*, also identified with one gene in the black porgy (**Table 3**), was reported to act as an essential transcriptional factor for steroidogenesis and for development of the reproductive axis [54].

Interestingly, five copies of *sox9* were also identified in the black porgy genome. Nevertheless, previous findings reported that only 2 paralogs of *sox9* (*sox9a* and *sox9b*) are present in zebrafish [55] and catfish [56]. *Sox9a* is usually associated with testicular development, while this may be linked with sex reversal in the tong sole [52]. In comparison, *sox9b* possesses a new function in the ovary [55]. In addition, we noticed that female-related genes (*wnt4*, *vasa* and *jnk1*) have multiple copies in our current study, which may be retained since the whole-genome duplication in the ancestor to the teleost. These genes have been proven to play important roles in ovarian growth and natural sex changes in fishes [57–60]. It was reported that two *wnt4* genes (*wnt4a* and *wnt4b*) are present in most teleost fish, while other vertebrates and invertebrates possess only a single *wnt4* gene. Furthermore, two copies of the *wnt4a*, *wnt4a1* and *wnt4a2*, exist in some teleost species resulting from the additional duplication of *wnt4* gene [61]. It has been shown that *wnt4a* was mainly expressed in the gonad, gill and brain of teleost fish (such as zebrafish [62] and rainbow trout [63]), and it was confirmed to be associated with sex reversal in the tongue sole [61]. The *vasa* gene, also called *ddx4*, was reported to

play an important role in gametogenesis and germ cell development [64]. Previous findings showed that *vasa* was a single copy gene in the majority of chordates, such as zebrafish [65,66]. However, 3 *vasa* genes were also reported in Nile tilapia (*Oreochromis niloticus*) [67]. *Jnk1* is closely associated with ovarian differentiation and development in fish. A previous finding [58] reported that *jnk1* highly transcribed in the ovary of the female ricefield eel (*Monopterus albus*), another teleost with natural sex-change from female to male, and reduced to a substantial level at the subsequent stage of intersex; hence, the data demonstrated that *jnk1* may play a key role in the sexual reversal. Surprisingly, two *jnk1* genes (*jnk1a* and *jnk1b*) were reported in the polyploid hybrids of red crucian carp (*Carassius auratus* red var.) and common carp (*Cyprinus carpio* L.) [68]. Interestingly, our data demonstrate that the distribution of these 3 types of genes in the black porgy genome is similar to that in ricefield eel (our unpublished results; Data of the *Monopterus* Whole Genome Shotgun project have been deposited at DDBJ/EMBL/GenBank under the accession number of AONE00000000). For example, 2 male-related genes (*piwil1 and piwil2*) are clustered together, while *lin28a* and *rspo1* are adjacent to each other. We also observed that most of these genes are congregated on the scaffolds 1, 2, 3, 11 and 15 (**Table 3**).

Conclusions

In summary, we sequenced and assembled the whole genome of Chinese black porgy. This is the first genomic report of Sparidae fish. Furthermore, we provided a genomic survey on the 26 genes potentially associated with sex change. The achieved genome data will be helpful for further biological and evolutionary studies. Furthermore, it will be valuable for implementation of molecular breeding, with substantial support from our genomic data, to obtain genetic improvement of this economically important teleost fish.

Table 1. Summary of the achieved genome assembly and annotation.

Genome assembly	Parameter
contig N50 size (kb)	17.2
contig number (> 100 bp)	115,091
scaffold N50 size (Mb)	7.6
scaffold number (> 100 bp)	31,359
Total length (Mb)	688.1
Genome coverage (×)	257.6
The longest scaffold (bp)	22,574,836
**Genome annotation**	**Parameter**
Protein-coding gene number	19,465
Mean transcript length (kb)	17.3
Mean exons per gene	11.1
Mean exon length (bp)	170.2
Mean intron length (bp)	1519.2

Table 2. Detailed classification of repeat sequences in the assembled genome.

Type	Repbase TEs		TE proteins		*Denovo*		Combined TEs	
	Length (Mb)	In genome (%)	Length (Mb)	In genome (%)	Length (Mb)	In genome (%)	Length (Mb)	In genome (%)
DNA	20.930	3.041	2.200	0.320	58.340	8.479	68.130	9.902
LINE	10.240	1.488	6.950	1.010	26.760	3.889	33.020	4.789
SINE	1.120	0.163	2.340	0.000	3.780	0.550	4.550	0.661
LTR	7.200	1.046	35.410	0.340	25.980	3.062	31.270	4.544
Other	0.020	0.003	0.000	0.000	0.000	0.000	0.020	0.003
Unknown	0.000	0.000	0.000	0.000	25.370	3.687	25.370	3.687
Total	35.300	5.130	11.480	1.669	124.540	18.099	136.240	19.780

Table 3. Three types of genes potentially related to sex change in the black porgy genome

**Sex determination and differentiation genes**		
**Gene**	**Copy number**	**Scaffold**
fst	2	10, 17
sox9	5	11, 13, 16, 19, 27
vasa	10	11, 14, 16, 20 , 27, 34, 37, 47, 53, 68
ctnnb1	4	2, 16, 64, 115
piwil1	1	15
piwil2	1	15
sf1	1	108
rspo1	2	2, 74
foxl2	2	1, 22
cyp19a1a	2	8, 28
gsdf	1	3
figla	1	32
dmrt1	1	56
wnt4	15	1, 2, 5, 6, 7, 8, 9, 18, 19, 20, 32, 34, 62 ,67, 122
dax1	4	2, 3, 14, 43
cyp11a1	2	8, 33
hsd3b1	2	7, 36
amhr2	2	9, 185
jnk1	9	1, 3, 4, 5, 16, 17, 38, 79, 117
**Pluripotency factors**		
**Gene**	**Copy number**	**Scaffold**
klf4	5	1, 3, 17, 96, 142
nr5a2	3	8, 19, 28
lin28a	2	2, 3
oct4	1	3
**Apoptosis factors**		
**Gene**	**Copy number**	**Scaffold**
traf2	2	3, 15
casp2	1	2
tnfr1	1	2

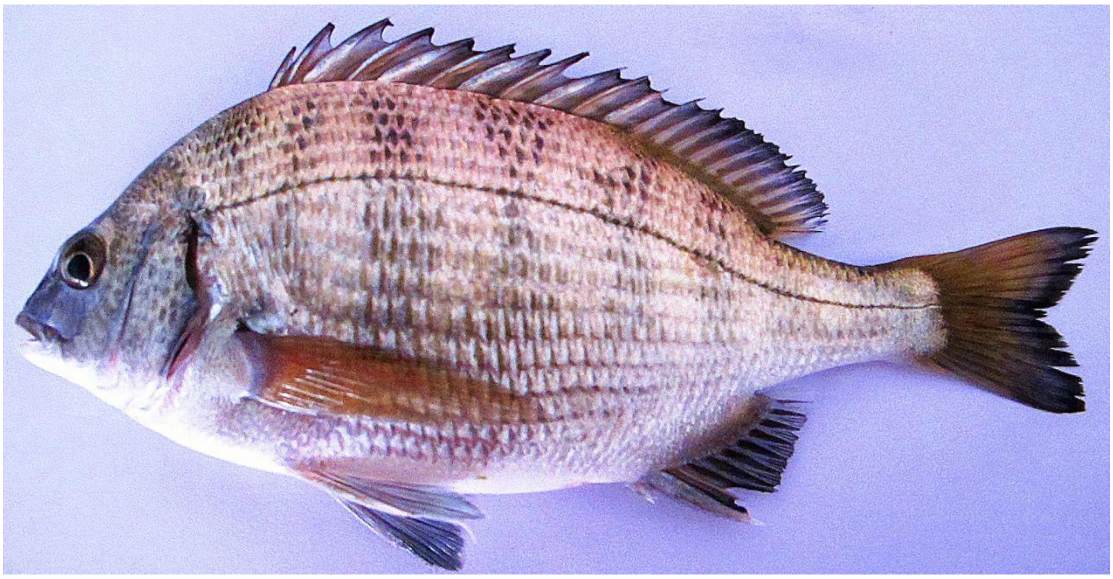

Figure 1. Image of a Chinese black porgy. It was captured from Laizhou Bay in Yantai, Shandong Province, China.

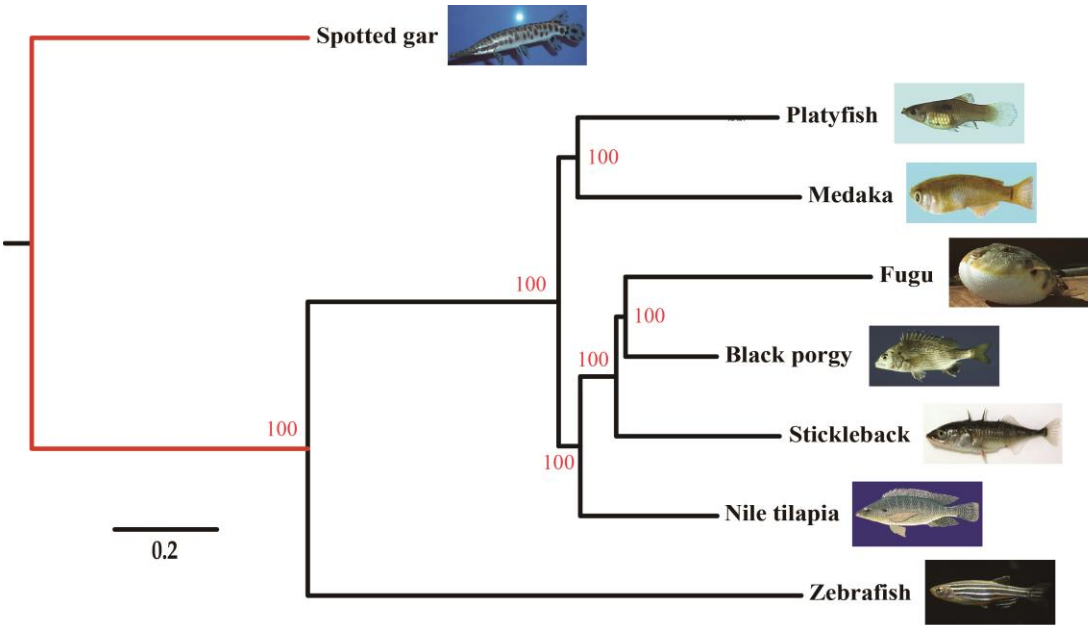

Figure 2. Phylogeny of ray-finned fishes. Spotted gar was used as the outgroup. The bootstrap supporting value for the topology is 100. These pictures in the phylogenetic tree were downloaded from the Fishbase.

Ethics approval and consent to participate

All animal experiments in this study were implemented in the light of the guidelines

of the Animal Ethics Committee and ratified by the Institutional Review Board on Bioethics and Biosafety of BGI, China

Availability of supporting data

The raw sequencing reads of all libraries and the transcriptome data have been deposited in the NCBI SRA database with accession numbers of SRA541936 and SRA587358. Supporting data are available in the GigaScience database, GigaDB.

Author's contributions

ZyZ, QS, and PX conceived the project. JX, CJ, JQ, FZ, HxL, HlL, DS, ZR and JC extracted the genomic DNA and performed genome sequencing. KZ, SC, ZwZ, XY, JZ, CB and JL assembled the genome and analyzed the data. TG, RG and JX participated discussions and provided valuable advice for revision. KZ, QS, ZyZ, PX, ZwZ and SC prepared the manuscript.

Acknowledgements

This work was supported by Aquatic Sanxin Engineering Major Project of Jiangsu Province (No. D2015-17), Key Research and Development (Modern Agriculture) Program of Jiangsu Province (No. BE2016326), Fund for Independent Innovation of Agricultural Science and Technology of Jiangsu Province (No. CX(17)2021), Aquatic Sanxin Engineering Project of Jiangsu Province (No.Y2016-23), Jiangsu Innovation Ability Construction Program (No. BM2015017), Nantong Applied Basic Research Program (No.MS12015071), Nantong Applied Basic Research Program (No. MS12015070 & MS12016029), and Zhenjiang Leading Talent Program for Innovation and Entrepreneurship.

Competing interests

The authors declare that they have no competing interests.

References

1. Gonzalez EB, Umino T, Nagasawa K. Stock enhancement programe for black sea bream, *Acanthopagrus schlegelii* (Bleeker), in Hiroshima Bay, Japan: a Review. Aquaculture Research 2008;**39**:1307-1315.

2. Zhang Y, Øverland M, Xie S et al. Mixtures of lupin and pea protein concentrates can efficiently replace high-quality fish meal in extruded diets for juvenile black sea bream (*Acanthopagrus schlegelii*). Aquaculture 2012;**354**:68-74.

3. Guo Z, Zhang W, Zhou Y et al. Feeding ratio and frequency affects cadmium bioaccumulation in black sea bream *Acanthopagrus schlegelii*. Aquaculture Environment Interactions 2015;**7**(2):135-145.

4. Murata O. Studies on the breeding of cultivated marine fishes. Bulletin of Fishery Laboratory, Kinki University 1998;**6**:1-101.

5. Kim YS, Biswas A, Ji SC et al. Phytase in soybean meal diet improves phosphorus availability of hybrid, female red sea bream *Pagrus major*× male black sea bream *Acanthopagrus schlegelii*. Aquaculture Science 2015;**63**(2):159-167.

6. Caporaso JG, Lauber CL, Walters WA et al. Ultra-high-throughput microbial community analysis on the Illumina HiSeq and MiSeq platforms. The ISME Journal 2012;**6**(8):1621-1624.

7. Li R, Yu C, Li Y et al. SOAP2: an improved ultrafast tool for short read alignment. Bioinformatics 2009;25(15):1966-1967.

8. Liu B, Shi Y, Yuan J et al. Estimation of genomic characteristics by analyzing k-mer frequency in denovo genome projects. Quantitative Biology 2013;**35**(s1-3):62-67.

9. Luo R, Liu B, Xie Y et al. SOAPdenovo2: an empirically improved memory-efficient short-read de novo assembler. Gigascience 2012;**1**:18.

10. Boetzer M, Henkel CV, Jansen HJ et al. Scaffolding pre-assembled contigs using SSPACE. Bioinformatics 2011;**27**:578-579.

11. Howe K, Clark M D, Torroja C F, et al. The zebrafish reference genome sequence and its relationship to the human genome. Nature 2013;**496**(7446):498.

12. Schartl M, Walter R B, Shen Y, et al. The genome of the platyfish, *Xiphophorus maculatus*, provides insights into evolutionary adaptation and several complex traits. Nature Genetics 2013;**45**(5):567-572.

13. Chen S, Zhang G, Shao C, et al. Whole-genome sequence of a flatfish provides

insights into ZW sex chromosome evolution and adaptation to a benthic lifestyle. Nature Genetics 2014;**46**(3):253-260

14. Xu P, Zhang X, Wang X, et al. Genome sequence and genetic diversity of the common carp, *Cyprinus carpio*. Nature Genetics 2014;**46**(11):1212-1219.
15. Wang Y, Lu Y, Zhang Y et al. The draft genome of the grass carp (*Ctenopharyngodon idellus*) provides insights into its evolution and vegetarian adaptation. Nature Genetics 2015;**47**(6):625-631.
16. Lien S, Koop BF, Sandve SR et al. The Atlantic salmon genome provides insights into rediploidization. Nature 2016;**533**:200-205.
17. Lin Q, Fan S, Zhang Y et al. The seahorse genome and the evolution of its specialized morphology. Nature 2016;**540**(7633):395-399.
18. Yang J, Chen X, Bai J et al. The Sinocyclocheilus cavefish genome provides insights into cave adaptation. BMC Biology 2016;**14**(1):1.
19. Parra G, Bradnam K, Korf I. CEGMA: a pipeline to accurately annotate core genes in eukaryotic genomes. Bioinformatics 2007;**23**(9):1061-1067.
20. Simão FA, Waterhouse RM, Ioannidis P et al. BUSCO: assessing genome assembly and annotation completeness with single-copy orthologs. Bioinformatics 2015;**31**(19):3210-3212.
21. Tarailo-GraovacM, Chen N. Using RepeatMasker to identify repetitive elements in Genomic sequences. Current Protocols in Bioinformatics 2009;chapter 4: unit **4** 10.
22. Maziade M, Bouchard S, Gingras N et al. Long-term stability of diagnosis and symptom dimensions in a systematic sample of patients with onset of schizophrenia in childhood and early adolescence. II: Postnegative distinction and childhood predictors of adult outcome. The British Journal of Psychiatry 1996;**169**(3):371-378.
23. Mario S, Oliver K, Irfan G et al. AUGUSTUS: ab initio prediction of alternative transcripts. Nucleic Acids Research 2006;**34**:435-439.
24. Burge C, Karlin S. Prediction of complete gene structures in human genomic DNA. Journal of Molecular Biology 1997;**268**(1):78-94.

25. Birney E, Clamp M, Durbin R. GeneWise and Genomewise. Genome Research 2004;**14**(5):988-995.

26. Trapnell C, Pachter L, Salzberg SL. TopHat: discovering splice junctions with RNA-Seq. Bioinformatics 2009;**25**(9):1105-1111.

27. Trapnell C, Williams BA, Pertea G et al. Transcript assembly and quantification by RNA-Seq reveals unannotated transcripts and isoform switching during cell differentiation. Nature Biotechnology 2010;**28**(5):511-515.

28. Elsik CG, Mackey AJ, Reese JT et al. Creating a honey bee consensus gene set. Genome Biology 2007;**8**(1):90-105.

29. Finn RD. Pfam: the protein families database. Nucleic Acids Research 2014;**42**(Database issue):D222-230.

30. Attwood TK. The PRINTS database: A resource for identification of protein families. Briefings in Bioinformatics 2002;**3**(3):252-263.

31. Bru C, Courcelle E, Beausse Y et al. The ProDom database of protein domain families: more emphasis on 3D. Nucleic Acids Research 2005;**33**(Database issue):212-215.

32. Letunic I, Copley RR, Schmidt S et al. SMART 4.0: towards genomic data integration. Nucleic Acids Research 2004;**32**(Databaseissue):D142-D144.

33. Boeckmann B, Bairoch A, Apweiler R et al. The Swiss-Prot knowledgebase and its supplement TREMBL in 2003. Nucleic Acids Research 2003;**31**(1):365-370.

34. Hunter S, Apweiler R, Attwood TK et al. InterPro: the integrative protein signature database. Nucleic Acids Research 2009;**37**(suppl 1):D211-D215.

35. Hingamp P, Broek AEVD, Stoesser G et al. The EMBL nucleotide sequence database. Molecular Biotechnology 1999;**12**(3):255-267.

36. Kanehisa M, Goto S. KEGG: kyoto encyclopedia of genes and genomes. Nucleic Acids Research 2000;**27**(1):29-34.

37. Cunningham F, Amode MR, Barrell D et al. Ensembl 2015. Nucleic Acids Research 2014;**43**(Database issue):D662-629.

38. Li L, Stoeckert CJ, Roos DS. OrthoMCL: identification of ortholog groups for eukaryotic genomes. Genome Research 2003;**13**(9):2178-2189.

39. Edgar RC. MUSCLE: multiple sequence alignment with high accuracy and high throughput. Nucleic Acids Research 2004;**32**(5):1792-1797.

40. Guindon S, Dufayard JF, Lefort V et al. New algorithms and methods to estimate maximum-likelihood phylogenies: assessing the performance of PhyML 3.0. Systematic Biology 2010;**59**(3):307-321.

41. Wu GC, Chang CF. The switch of secondary sex determination in protandrous black porgy, *Acanthopagrus schlegeli*. Fish Physiology and Biochemistry 2013;**39**(1):33-38.

42. Xiao YM, Chen L, Liu J et al. Contrast expression patterns of *jnk1* during sex reversal of the rice-field eel. Journal of Experimental Zoology Part B: Molecular and Developmental Evolution 2010;**314**(3):242-256.

43. Webster KA, Schach U, Ordaz A et al. *Dmrt1* is necessary for male sexual development in Zebrafish. Developmental Biology 2017;**422**(1):33-46.

44. Mount DW. Using the basic local alignment search tool (blast). Cold Spring Harbor Protocols 2007;**2007**(7):pdb.top17.

45. Wu GC, Du JL, Lee YH et al. Current status of genetic and endocrine factors in the sex change of protandrous black porgy, *Acanthopagrus schlegeli* (Teleostean). Annals of the New York Academy of Sciences 2005;1040(1):206-214.

46. Wu G C, Chiu P C, Lin C J et al. Testicular *dmrt1* is involved in the sexual fate of the ovotestis in the protandrous black porgy. Biology of Reproduction 2012; 86(2).

47. Wu G C, Tomy S, Nakamura M et al. Dual roles of *cyp19a1a* in gonadal sex differentiation and development in the protandrous black porgy, *Acanthopagrus schlegeli*. Biology of Reproduction 2008;79(6):1111-1120.

48. Chen S, Zhang G, Shao C et al. Whole-genome sequence of a flatfish provides insights into ZW sex chromosome evolution and adaptation to a benthic lifestyle. Nature Genetics 2014;**46**(3):253-260.

49. Cui Z, Liu Y, Wang W et al. Genome editing reveals *dmrt1* as an essential male sex-determining gene in Chinese tongue sole (*Cynoglossus semilaevis*). Scientific Reports 2017;**7**:42213.

50. Nanda I, Kondo M, Hornung U, et al. A duplicated copy of *dmrt1* in the sex-determining region of the Y chromosome of the medaka, *Oryzias latipes*. Proceedings of the National Academy of Sciences 2002; 99(18):11778-11783.

51. Masuyama H, Yamada M, Kamei Y, et al. Dmrt1 mutation causes a male-to-female sex reversal after the sex determination by Dmy in the medaka. Chromosome Research 2012;20(1):163-176.

52. Dong X, Chen S, Ji X et al. Molecular cloning, characterization and expression analysis of *sox9a* and *foxl2* genes in half-smooth tongue sole (*Cynoglossus semilaevis*). Acta Oceanologica Sinica 2011;**30**(1):68-77.

53. Li H, Xu W, Zhang N et al. Two Figla homologues have disparate functions during sex differentiation in half-smooth tongue sole (*Cynoglossus semilaevis*). Scientific Reports 2016;**6**:28219.

54. Xie QP, He X, Sui YN et al. Haploinsufficiency of *sf1* Causes Female to Male Sex Reversal in Nile Tilapia, *Oreochromis niloticus*. Endocrinology 2016;**157**(6):2500-2514.

55. Rodriguez-Mari A, Yan YL, Bremiller RA et al. Characterization and expression pattern of zebrafish Anti-Mu¨llerian hormone (*Amh*) relative to *sox9a*, *sox9b*, and *cyp19a1a*, during gonad development. Gene Expression Patterns 2005;**5**:655–667.

56. Raghuveer K, Garhwal R, Wang DS et al. Effect of methyl testosterone-and ethynyl estradiol-induced sex differentiation on catfish, *Clarias gariepinus*: expression profiles of *dmrt1*, Cytochrome P450 aromatases and 3 beta-hydroxysteroid dehydrogenase. Fish Physiology and Biochemistry 2005;**31**(2):143-147.

57. Ye D, Lv D, Song P et al. Cloning and characterization of a rice field eel *vasa-like* gene cDNA and its expression in gonads during natural sex transformation. Biochemical Genetics 2007;**45**(3-4):211-224.

58. Xiao YM, Chen L, Liu J et al. Contrast expression patterns of *jnk1* during sex reversal of the rice field eel. Journal of Experimental Zoology Part B 2010;**314**(3):242-256.

59. Böhne A, Wilson CA, Postlethwait JH et al. Variations on a theme:Genomics of sex determination in the cichlid fish *Astatotilapia burtoni*. BMC Genomics 2016;**17**(1):883.

60. Bernard P, Harley V. Wnt4 action in gonadal development and sex determination. The International Journal of Biochemistry & Cell Biology 2007;**39**(1):31–43.

61. Hu Q, Zhu Y, Liu Y et al. Cloning and characterization of *wnt4a* gene and evidence for positive selection in half-smooth tongue sole (*Cynoglossus semilaevis*). Scientific Reports 2014;**4**:7167.

62. Matsui T, Raya Á, Kawakami Y et al. Noncanonical Wnt signaling regulates midline convergence of organ primordia during zebrafish development. Genes & Development 2005;**19**(1):164-175.

63. Nicol B, Guerin A, Fostier A, et al. Ovary-predominant *wnt4* expression during gonadal differentiation is not conserved in the rainbow trout (*Oncorhynchus mykiss*). Molecular Reproduction and Development 2012;**79**(1):51-63.

64. Lüking A, Stahl U, Schmidt U. The protein family of RNA helicases. Critical Reviews in Biochemistry and Molecular Biology 1998;33(4):259-296.

65. Yoon C, Kawakami K, Hopkins N. Zebrafish *vasa* homologue RNA is localized to the cleavage planes of 2-and 4-cell-stage embryos and is expressed in the primordial germ cells. Development 1997;124(16):3157-3165.

66. Krøvel AV, Olsen LC. Sexual dimorphic expression pattern of a splice variant of zebrafish *vasa* during gonadal development. Developmental biology 2004;271(1):190-197.

67. Fujimura K, Conte MA, Kocher TD. Circular DNA intermediate in the duplication of Nile tilapia *vasa* genes. PLoS One 2011;6(12):e29477.

68. Xiao YM, Jiang MG, Luo Z W, et al. Identification and analysis of the *jnk1* gene in polyploid hybrids of red crucian carp (*Carassius auratus* red var.) and common carp (*Cyprinus carpio* L.). Genetics and Molecular Research 2014;13(1):906-919.
