# Supplementary material for: Draft genome of the protandrous Chinese black porgy, Acanthopagrus schlegelii
Source: Gigascience. 2018 Feb 26;7(4):giy012. doi: 10.1093/gigascience/giy012 (PMC5893958; doi:10.1093/gigascience/giy012)
Supplement: Response-to-Reviewer-Comments_Original-Submission.pdf [file giy012_response-to-reviewer-comments_original-submission.pdf]

Author's Response To Reviewer Comments

Dear Editor,
Thanks for your kind help. We also appreciate the instructive comments from the two reviewers. According to their suggestions, we performed additional analyses and made a careful revision on our previous manuscript. Our point-by-point responses are attached for your consideration.
We removed the conclusion about compact genome and revised the “high quality” statement. Addition of the section on Author’s contributions was done in accordance with your advice. By the way, the current version was revised with help from MDPI, a professional English editing company.
Best regards,
Qiong Shi, PhD, Professor
BGI
Shenzhen 518083
China

Reviewer 1:
General comments
Hermaphroditism is an interesting method of reproduction, where individuals change sex during their lifetime, or produce both eggs and sperm at the same time. While the genotypic basis for sex-determination in gonochoristic species (those that do not change sex) is being elucidated in more and more species, knowledge about the genotypic basis for sex change in hermaphrodite fishes is currently lacking.

The authors produce a genome assembly of the protandrous (male first) Chinese black porgy, Acanthopagrus schlegelii, annotates it, and do a survey of genes that are known to be involved in sex determination and differentiation. I greatly support publication of genome assemblies, and teleost genome assemblies in particular. The more data available for analysis for scientists, the better. However, I find the claims regarding the high quality of the genome assembly to be unsubstantiated. Quality is a difficult measure, but if it has to be used, then it has to be shown that something is better than something else, or at least equal to something of high quality. If the authors had shown that more complete genes are found with CEGMA and BUSCO than in other teleost genome assemblies, then I would have been convinced that this assembly is of high quality. They do not do that. As the genome assembly is presented in this manuscript, it does not warrant the modifier 'high quality'.

Answer: Thanks for your comments. You are right. We should compare our results, for example the scaffold N50 value, with others. We also replaced “high quality” with a statement of “a relatively high level”. Please see more details about related changes on lines 111-116 of the revised manuscript.

The authors also claim that black porgy has a compact genome, but do not shown much data that supports this. It has a similar genome size to many other teleosts, such as medaka, platyfish, Atlantic cod, Amazon molly, and substantially larger than species with known compact genomes such as fugu, tetraodon, stickleback and pipefishes. If the authors want to make a point of the supposed compactness of the black porgy genome, they need to make a much stronger case than

this.

Answer: Thanks for your advice. The claim about compact genome was removed in the revised manuscript.

The authors do a survey of different genes associated with sex determination and differentiation in different species, but it is not clear to me why this is done. No further analysis than stating that these are present was done. Was the presence of these genes surprising? Then that should be stated properly. While the methods of sex determination and differential might not be conserved through teleosts, I do think we would expect the presence of most of these genes in most teleosts. In addition, the purpose for this analysis needs to be justified. Was it performed as a quality control of the genome assembly?

Answer: Thanks for your comments and advice. In fact, some genes have been confirmed to be involved in sex change of the black porgy (lines 188-190). We mentioned them for a quality control of the genome assembly, as you mentioned, and also provide more information (such as scaffold location and copy number, see more details in Table 3) to support the sex-change phenomenon. The latter will be definitely helpful for further investigation on molecular mechanisms of sex change. Please find more explanations on lines 177-241 of the revised manuscript. Interestingly, we also proposed possible existence of sex chromosome in this teleost fish (lines 239-241).

The specific programs, version and settings used for some of the analyses are not stated. Please address this because it is important for assessing the method and approach used, in addition to reproducibility.

Answer: Thanks for your nice advice. Information about version and settings for each specific program was provided in the revised manuscript.

I cannot recommend publication of this manuscript before the issues I highlight above and below are addressed.

Specific comments

Abstract:
Please do not use abbreviations such as "what's" in the abstract.

Answer: Yes, it is done. Thanks for your advice. Please find corresponding change (Furthermore) on line 36 of the revised manuscript.

I am not sure that the black porgy is a "perfect" model for investigating sex change. It might very well be a good model.

Answer: Thanks for your advice. We changed "perfect" to "good" on line 36 of the revised manuscript.

Please change "…due to its protandrous hermaphrodite" to something like "…due to it being a protandrous hermaphrodite".

Answer: Yes, it is done. Please find corresponding change on line 37 of the revised manuscript.

Please distinguish between a genome and a genome assembly. You can obtain a high-quality genome assembly, but not a high quality genome.

Answer: Yes, you are right. Please find corresponding change on line 38 of the revised manuscript.

How do the authors assess "high-quality sequence reads"? High quality compared to what?

Answer: Thanks for your question. In fact, before assembly of the sequencing reads, SOAPfilter v2.2 software (Li et al. 2009) [7] with default parameters (-y -p -g 1 -o clean -M 2 -f 0) was utilized to remove low-quality raw reads (including reads with 10 or more non-sequenced/low-quality bases), PCR duplicates and adaptor sequences. We hence changed “high-quality” to “clean” on line 40 and added related procedure on lines 88-91 of the revised manuscript.

Please use lower case "scaffold" instead of capital letter in "…achieved Scaffold N50…".

Answer: Yes, it is done (on lines 43 and 111 of the revised manuscript).

Please use correct SI system units. Capital letter K, such as in Kb, is not a valid SI-system prefix (seehttps://en.wikipedia.org/wiki/Metric_prefix if unsure). kb is the correct abbreviation.

Answer: Thanks for your good advice. We corrected them on lines 45 and 150 of the revised manuscript.

The last sentence of the findings part of the abstract should be rewritten to more correct and accurate English.

Answer: Thanks for your nice comments. We rewrote the sentence in the revised manuscript (on lines 45-48) as follows.
By performing a comparative genomic analysis, we found three types of genes potentially associated with sex change, which are useful for the prediction of related genetic basis for the interesting protandrous hermaphrodite.

How do the authors assess the high quality of the genome assembly? High quality compared to what?

Answer: Thanks for your good question. In fact, we ran CEGMA and BUSCOv2 using the actinopterygii dataset on our genome assembly to evaluate the genome quality. The CEGMA results suggest that 90.7% CEGs are complete and 92.3% are partial; The BUSCO score was 91.0%, (C:91.0% [S:88.1%,D:2.9%], F:1.1%, M:7.9%, n:4584). We also compared our results, for example the scaffold N50 value, with others. We already replaced “high quality” with a

statement of “a relatively high level” on lines 111-124. Hence, here we provided a draft genome assembly of the Chinese black porgy (please find the statement on line 49 of our revised manuscript).

Data description:
How do the authors assess that the hybrid of Japanese seabream and Chinese black porgy is "excellent"? It can be "good" or "important", but I am unsure of how it can be "excellent".

Answer: Thanks for your advice. We changed "excellent" to "good" on line 70 of the revised manuscript.

I find the description of "improving the quality of sequencing reads" lacking. First, it is not possible to improve the quality of these. The sequencing machine sets the quality of these. I would have written something along the lines of "Before assembly of the sequencing reads, those with low-quality bases…". How was the removal of these reads done? Which program and which settings? Again, in what way is the resulting clean reads of "high-quality"? Is it necessary to specify the quality of these?

Answer: Thanks for the nice comments. We changed “high-quality” to “clean” on line 40. We also added the following sentence in the revised manuscript (on lines 88-91) for a clear statement.
Before assembly of the sequencing reads, SOAPfilter v2.2 software (Li et al. 2009) [7] with default parameters (-y -p -g 1 -o clean -M 2 -f 0) was utilized to remove low-quality raw reads (including reads with 10 or more non-sequenced/low-quality bases), PCR duplicates and adaptor sequences.

How can it be stated that the result will be a good quality genome assembly before running SOAPdenovo2? Better skip the word "quality" here, and just write "To obtain a genome assembly, we ran SOAPdenovo2…"

Answer: Thanks for your nice advice. Yes, it is done. Please find the change on line 99 of the revised manuscript.

In the assembly section, the authors suddenly mention "error corrected [sic]" reads, without mentioning how these were created. Please describe how the error-correction was performed, which program and which settings.

Answer: Thanks for your suggestions. Sorry for the mistake. The sentence was rewritten on lines 101-103, and a clear statement was added on lines 88-91 of the revised manuscript.

Why applying SSPACE on the scaffolds created by SOAPdenovo2? Does it create even longer and better scaffolds? How was the optimized parameters found?

Answer: Yes, the SSPACE was employed to create longer and better scaffolds. In order to find the optimized parameters, we tested multiple different settings. The -K option of SOAPdenovo2 was set at 25, 27, 29, 31 and 35 for trial. Finally, we observed that the –K set at 27 could

generate a good genome assembly. The other parameters of SOAPdenovo were defaulted without change.

Again, "scaffold" with lower case first letter, and not capital.

Answer: Yes, it is done (on line 111 of the revised manuscript).

I like that CEGMA and BUSCO was used to validate the genome assembly. However, it is not specified whether or not the 92 % of CEGs found were complete or not. 92 % partial hits of CEGs are not especially impressive, but 92 % of complete hits are of course better.

Answer: The sentence was changed as follows on line 119.
The estimates suggest that 90.7% CEGs are complete and 92.3% are partial.
Hence, we stated that we obtained a draft genome of the black porgy (line 49).

In addition it is not specified which dataset was used for BUSCO. For BUSCOv3, the possible datasets (also called assessment sets) are Eukaryota, Metazoa, Vertebrata or Actinopterygii. However, while BUSCO version 3 is mentioned, it does not look like version 3 was used. From the number of genes found (n: 843), it looks like version 1 of BUSCO was run on the metazoan dataset, see Simão et al 2015. Since BUSCOv3 is mentioned, I would rather see the performance on the Actinopterygii dataset (found at http://busco.ezlab.org/) with BUSCOv3. Then http://www.biorxiv.org/content/early/2017/08/17/177485 should also be referred, where BUSCOv3 is described. For examples of performance of BUSCO on fish genome assembles, see for instance Jansen et al 2017 with BUSCOv3 run on the European eel genome assembly with the vertebrate dataset, finding 79.8 % complete genes (of 2586), or Liu et al 2017 where they find 81.4% complete and 9.1% partial vertebrate BUSCO genes (of 3023) using BUSCOv1 in the blunt snout bream genome assembly.
With the availability of the genome assembly, I downloaded it and ran CEGMA and BUSCOv2 using the actinopterygii dataset on it. While the results might vary from run to run or computer to computer, I find 86.3 % complete and 89.1 % partial genes with CEGMA. With BUSCOv2 I find 91.0 % complete genes (C:91.0%[S:88.1%,D:2.9%],F:1.1%,M:7.9%,n:4584). This is less than some of the genome assemblies I have looked at, but the authors should do their own comparisons.
After addressing these two issues (partial or complete CEGMA, correct dataset and version of BUSCO), then the authors can then assess whether or not the genome assembly is of high quality by comparing to other fish genome assemblies, for instance those two mentioned or other relevant ones.

Answer: Thanks for your comments. According to your suggestions, we ran CEGMA and BUSCOv2 using the actinopterygii dataset on our genome assembly. The CEGMA estimates suggest that 90.7% CEGs are complete and 92.3% are partial.

Table A. Statistics of the completeness of the genome based on 248 CEGS.
Prots Completeness (%)
Complete 225 90.73
Group 1 62 93.94

Group 2 48 85.71
Group 3 55 90.16
Group 4 60 92.31
Partial 229 92.34
Group 1 63 95.45
Group 2 49 87.50
Group 3 57 93.44
Group 4 60 92.31

The final BUSCO score was 91.0%, (C:91.0% [S:88.1%, D:2.9%], F:1.1%, M:7.9%, n:4584). These results from CEGMA and BUSCO suggested the assembled genome covers majority of the gene space.
You are right. The CEGMA and BUSCO scores are good, but not outstanding. We hence revised these sentences (on lines 119-124 of the new manuscript).

It is good a de novo repeat library was created. However, direct comparisons of repeat content between different species are not straightforward. For instance, the difference of 23.70 % repeats in black porgy compared to 25.4 % in Atlantic cod and 25.2 % in stickleback is likely not significant. Different approaches would give different numbers. Also, at 739.6 Mbp Chinese black porgy might have a smaller genome than some other fishes, but it is not especially compact compared to fugu, tetraodon, stickleback or the different pipefish and sea horse genomes.

Answer: Thanks for the nice comment. We removed these sentences about comparisons of repeat content between different fish species in the revised manuscript.

The authors might confuse GENSCAN (identifying gene structures) with GENESCAN (analysis of sequencing data from ABI sequencer).

Answer: Sorry for the mistake. It was corrected on line 134 of the revised manuscript.

Use of transcriptome data can greatly improve the annotation of a genome assembly, but I am missing a better description of the sequencing data. Which program and which settings were used to remove low-quality bases, adapter and duplicated sequences? I am a bit surprised that TopHat1.2 was used. From the website (http://ccb.jhu.edu/software/tophat/index.shtml) it seems it was released in 2011. While researchers should keep up to date on which versions of relevant software is used, there might be specific reasons why some software used is out-of-date. However, 6 years old software is a bit of a stretch. Please use a newer version, and consider using HISAT2 instead.

Answer: Thanks for the nice advice. SOAPfilter v2.2 software (Li et al. 2009) [7] with default parameters (-y -p -g 1 -o clean -M 2 -f 0) was utilized to remove low-quality raw reads (including reads with 10 or more non-sequenced/low-quality bases), PCR duplicates and adaptor sequences. According to your suggestion, the newer version (TopHat2.1.1) was applied to predict gene structures. Please find more details about the changes on lines 88-92 and 147-147 of the revised manuscript.

The low number of annotated genes surprised me. Many fish species usually have about 20,000 genes or more annotated. These seem few and short, and I wonder if something went wrong during the annotation process. For instance, annotated fish genome assemblies in Ensembl of similar size (platyfish, medaka, and Amazon molly) have mean gene lengths around 13 - 17 kbp. However, the authors write that the average transcript length is 8.5 Kb (should be kb). In addition, the average number of exons per gene in those species is 11-15, while in black porgy it is 8.7. Too much of the assembly might have been masked for instance. I would like the authors to address this by doing a good validation of the annotation or redoing the annotation if it is faulty. BUSCO can also be used to validate the annotation, and I downloaded the predicted CDS and ran BUSCO on them. I found 65.4 % complete genes using the actinopterygii dataset (C:65.4% [S:63.0%, D:2.4%], F:4.6%, M:30.0%, n:4584). I am unsure how this compares to other species. From my experience, it is not unusual to find fewer genes in the predicted transcriptome than in the genome assembly.

Answer: Thanks for the nice comments. Annotation of the genome assembly was redone. The final gene set contains 19,465 genes, with an average transcript length of 17.3 kb (on lines 148-149 of the revised manuscript).
We ran BUSCOv2 again on the predicted CDS, and the final BUSCO score was 85.2% (C:85.2% [S:82.2%, D:3.0%], F:2.9%, M:11.9%, n:4584). For the genome assembly, the BUSCO score reached to 91.0%, (C:91.0% [S:88.1%, D:2.9%], F:1.1%, M:7.9%, n:4584).
By the way, please find more details about Annotation on lines 125-156.

I protest to the use of "proved" and "nice" when describing functional motifs and domains in the predicted genes/proteins. Rather write that "90 % of the predicted genes from the assembled genome contain at least one related functional…". The last sentence of this paragraph (starting with "These nice data...") can be skipped.

Answer: Thanks for your nice advice. We rewrote this sentence (on lines 154-156 of the new manuscript) and removed the last sentence of this paragraph in the revised manuscript.

Phylogenetic analysis:
Why was the phylogenetic analysis done? Was the placement of black porgy among teleosts uncertain? I guess that Betancur-R et al 2017 might have come out too late to be used in the writing of this manuscript, but in that publication several other Acanthopagrus species are investigated and placed in the phylogeny. It is puzzling to perform an analysis without using it to draw any conclusions (expectations in gene content for instance).

Answer: Yes, the phylogenetic analysis, on a genomic view, was performed to investigate the placement of black porgy among teleosts. We removed certain conclusions since they were not supported with solid evidence.

Was the predicted transcript sequence for each protein used, or was each amino acid in the protein sequence from the MUSCLE alignment changed into the most common codon? If the latter, that would be strange and a lot of signal would be lost, if the first, that is a valid and proper approach. After looking at the script, it seems that the first is the approach used here. Please describe this section better.

Answer: Thanks for your nice advice. You are right. The protein alignments were changed to corresponding transcript sequences using an in-house perl script. All these nucleotide sequences of each species were integrated into a supergene, which were used to build a phylogenetic tree using PhyML. Please find the corresponding changes on lines 167-172 of the revised manuscript.

I do not agree with the assertion that black porgy has a "close relationship" with fugu. These two species are separated by almost 100 million years of evolution (Betancur-R et al 2017) and are not close. The presumed closeness of these two species does not seem to affect any of the conclusions or speculations in this paper. Of the species included in the phylogeny, platyfish and medaka both have around 600-700 Mbp size genome assemblies, tilapia and zebrafish have a bit larger (1-1.4 Gbp), while stickleback and fugu have smaller assemblies around 400-450 Mbp. It seems strange to compare directly to fugu when the size of the genome of black porgy is 200-300 Mbp larger, and quite similar in size to medaka and platyfish. That is, it is not especially compact.

Answer: Thanks for the nice comments. We reconstructed the phylogenetic tree and the bootstrap supporting value for the topology was set at 100. We also referred to the phylogenetic tree, which was built by Betancur-R et al and found that several other Acanthopagrus species (Acanthopagrus latus, Acanthopagrus catenula) have a closer relationship with fugu than other species investigated in this study.
You are right, the genome of Porgy is not especially compact. Hence, we removed this sentence in the revised manuscript.

Analysis of three types of genes for sex change:
What is the purpose of this analysis? Were all of the genes downloaded from NCBI found in the genome assembly? How was homology asserted? Homology presumes common ancestry, which would need to be tested in some way. A highly confident BLAST hit could be enough for this, but the authors do not state what cut-offs were used.

Answer: Thanks for your comments.
The black porgy is a good model for investigation on the molecular mechanisms of sex change. In this present study, we provided a genomic survey on these genes associated with sex-change phenomenon in the assembled genome, which will offer a useful genetic resource for studying the interesting protandrous hermaphrodite.
Protein sequences of the three main types of genes potentially associated with sex change were downloaded from NCBI and used for homology searches against the black porgy genome with tBlastn (version2.2.19). We chose alignments with coverage > 70% and identity > 70% for further prediction of gene structures using Genewise (version 2.2.0).
Please find corresponding changes on lines 179-186 of the revised manuscript.

The authors report that Wnt4, vasa and JNK1 are found in multiple copies in the genome assembly, and claim that these might be related to whole-genome duplication. Is it the teleost specific whole-genome duplication they think of, or do they propose additional genome duplication in the lineage leading to black porgy? If the latter, this should be investigated in more detail and properly argued. There are some genome duplications in teleosts, the lineage leading

to carps and salmonids are two well-known examples.

Answer: Thanks for your advice. Black porgy is a diploid species, hence multiple copies of wnt4, vasa and JNK1 in the genome assembly may be resulted from the teleost-specific whole genome duplication. Although the current assembly is the first draft of black porgy genome, more investigations are on-going.

This manuscript lacks several declaration sections. While it is stated in the text that "All animal experiments in this study were implemented in the light of the guidelines of the Animal Ethics Committee and ratified by the Institutional Review Board on Bioethics and Biosafety of BGI", it would be good to have this as a separate section also.

Answer: Yes, it’s done. Please find the separate section on lines 319-322 of the revised manuscript.

With 22 authors, and 6 of them contributing equally, I would also like to see an "author's contributions" section.

Answer: Yes, it’s done. Please find the separate section of Author’s contributions on lines 327-332 of the revised manuscript.

Reviewer #2:
The present Data Note reports the genome assembly of the black porgy, a teleost fish species of economic importance for the fisheries industry and of interest to understand ontogenetic sex change among vertebrates.

Standard methods have been used to generate a genome assembly of acceptable quality and the dataset has re-use potential. However, there are several issues that should be addressed before the manuscript is ready for publication. Please see a list below.
In addition, the level of writing needs to be improved with the help of a native speaker.

Answer: Thanks for your advice. The current version was revised with help from MDPI, a professional English editing company.

Specific points:
l. 61: confirm that "sea bread" is indeed a common name for this species

Answer: Sorry for the mistake. It was removed on line 60 of the revised manuscript.

l. 103 and l. 109/110: How were the parameters for SOAPdenovo and SSPACE optimized?

Answer: Thanks for the good question. In fact, in order to find the optimized parameters, we tested multiple different settings. The -K option of SOAPdenovo2 was originally set at 25, 27, 29, 31 and 35 for trial. Finally, we observed that the –K set at 27 could generate a good genome assembly. The other parameters of SOAPdenovo were defaulted without any change. For the

SSPACE, we employed its default parameters. We corrected this section with more information on lines 99-101 & 106-108 of the revised manuscript.

l. 114-116: The other fish genome assemblies to which the black porgy assembly is compared appear random. Why did you choose these few assemblies for comparison?

Answer: Thanks for the nice comments. In fact, we referred to the scaffold N50 values of multiple representative fish genome assemblies, which were published in good journals such as Nature and Nature Genetics. More data about zebrafish, platyfish, coelacanth, half-smooth tongue sole, elephant shark and common carp were also provided in the revised manuscript (lines 111-116).

l. 117-124: The CEGMA and BUSCO scores are good, but not outstanding. It is difficult to grasp the quality of the assembly from just providing these numbers and the authors could for example obtain CEGMA/BUSCO scores from the other fish genomes in l.114-116 for a more meaningful evaluation and comparison.

Answer: Thanks for your good advice. Based on your advice, we revised the sentence as follows: These results from CEGMA and BUSCO suggested the assembled genome covered majority of the gene space. Please find the corresponding change on lines 123-124 of the revised manuscript.

l.129-133: I do not follow the conclusion that the black porgy has a particularly compact genome and is prominently sparse in repeat content. It is within the range of e.g. cod and stickleback, while zebrafish is a known outlier with an unusual high repeat content. The estimated porgy genome size also does not imply a specifically compact genome.

Answer: Sorry for the misunderstanding. We remove the statement of compact genome in the revised manuscript.

l. 138: How did you obtain the "homologous proteins of several reported fish"?

Answer: The homologous proteins of several reported fishes, including zebrafish, Japanese puffer, stickleback and medaka, were downloaded from Ensembl release 75. Please find the new information on lines 135-138 of the revised manuscript.

l. 142-144: At which level did you generate the mixture of transcriptomic data. Did you mix mRNA, cDNA, RNA-seq libraries or reads in silico?

Answer: Sorry for the misleading description. In fact, we mixed cDNAs from these tissues, which was transcribed from mRNAs. Please see more details on lines 141-143 of the revised manuscript.

Phylogenomic analysis:
- How did you chose the species to include in the analysis?
- Please provide bootstrap values for your tree?
- Do you obtain a similar tree with different, e.g. Bayesian, methods?

- How does the obtained tree compare to the known phylogenetic position of black porgy?

Answer: Thanks for the nice comments. The selected fish species were representative and widely used for genomics study since their data are very good. In additional, we provided bootstrap value for the tree in the revised manuscript (lines 172-173).
According to your suggestion, we reconstructed the phylogenetic tree using MrBayes (Version 3.2), and obtained the same tree topology from both the Bayesian phylogenetic and the PhyML phylogenetic methods. We also referred to the phylogenetic tree built by Betancur-R et al and found that several other Acanthopagrus species (Acanthopagrus latus, Acanthopagrus catenula) have a closer relationship with fugu. Hence we removed the statement about closer relationship to fugu.

The grouping of porgy with pufferfish leads the authors to imply that there is shared genome compaction among them. Is this supported by knowledge about genome compaction in the pufferfish lineage? Again, I don't agree with the conclusions that the porgy genome is particularly compact.

Answer: You are right, hence we removed the sentence about compact genome in the revised manuscript.

l. 195-197: It would be important to mention that a duplicate of dmrt1 is the male sex determination gene in medaka.

Answer: Yes, it’s done. Please find the addition of such information on lines 201-203 of the revised manuscript.

l. 213: The term isoform should only be used for splice variants and not for gene duplicates such as sox9a and sox9b, which were generated during the teleost genome duplication.

Answer: You are right. We changed “isoforms” to “isotypes” on lines 210 & 216 of the revised manuscript.

Please follow the established gene nomenclature for fish genes.

Answer: Yes, it’s done. Thanks for your advice.

Table 3: Gene name should be oct4, not 4-Oct.

Answer: Sorry for the mistake. We corrected it in the revised Table 3 of the new manuscript.

Analysis of candidate genes for sex determination and sex change:
It is curious that there are a number of genes in the list with multiple copies in the assembly. Please provide more details on these cases. Are these extra copies different alleles, copy number variants or potential redundancy artifacts of the genome assembly? How do these extra copies relate to the teleost whole genome duplication and other known duplications of these genes in fish and vertebrates in general?

Finally, it would be good to provide the predicted sequences of the candidate genes as a separate sequence file.

Answer: Thanks for the nice comments. Several genes, such as Wnt4, vasa and JNK1, with multiple copies in the assembly may be copy number variants. We cannot rule out potential redundancy artifacts of the genome assembly, hence we are going to clone them before submission to NCBI for public availability.

l. 230-238: Is the similarity of genetic linkage of some of the sex-related genes in porgy and rice eel a specific characteristic of these two lineages or are they rather an indication of conserved synteny among teleost fishes in general?
Please explain better how genes distributed among several scaffolds (1, 2, 3, 11, 15) are a potential indication for a sex chromosome. Also, would you expect a species with ontogenetic sex change to have a sex chromosome at all? Please explain.

Answer: Sorry, we didn’t check the synteny conservation between the two fish species because the ricefield eel work has not been published yet. However, although we didn’t expect existence of a sex chromosome, it is still possible since these genes are distributed on only a few of scaffolds. The data are similar to ore previously reported Asian arowana (Bian et al., 2016, Scientific Reports, 6:24501), which was identified with sex chromosomes.
