# Supplementary material for: Draft genome of the protandrous Chinese black porgy, Acanthopagrus schlegelii
Source: Gigascience. 2018 Feb 26;7(4):giy012. doi: 10.1093/gigascience/giy012 (PMC5893958; doi:10.1093/gigascience/giy012)
Supplement: Response-to-Reviewer-Comments_Revision-1.pdf [file giy012_response-to-reviewer-comments_revision-1.pdf]

Author's Response To Reviewer Comments

Dear Editor,
Thanks for your kind help. We also appreciate the instructive comments from the two reviewers. According to their suggestions, we performed additional analyses and made a careful revision on our previous manuscript. Our point-by-point responses are attached for your consideration.

Please also include the details on your BUSCO analysis in the manuscript (and we will also ask you to upload BUSCO results to GigaDB). Why was Busco v2 used, when the latest version is v3?

Answer: Thanks for your good advice. We also ran BUSCO v3 on the genome assembly, and the final BUSCO score was 89.1% (C:89.1% [S:86.2%, D:2.9%], F:2.5%, M:8.4%, n:4584). For the predicted gene set, the new BUSCO score reached 85.5%, (C:85.5% [S:82.3%, D:3.2%], F:2.8%, M:11.7%, n:4584). Please find more details about these revisions on lines 118-121 and 148-150 in the revised manuscript.
In addition, we uploaded corresponding BUSCO results to the GigaDB.

Highlighting revisions to the manuscript in color can help us and the reviewers to quickly assess your next version.

Answer: Yes, it's done. We highlighted all the revisions in yellow for your convenience.

Finally, please note that GigaScience allows a maximum number of 3 indications of co-first authorship and a maximum number of 2 co-corresponding authors. Please revise accordingly.

Answer: Thanks for your comments. We revised the authorship information in accordance with your instructions. Three co-first authors (Prof. Zhang, Mr. Zhang and Dr. Chen) and two co-corresponding authors (Profs. Xu and Shi) are marked in the revised manuscript.

Best regards,
Qiong Shi, PhD, Professor
BGI
Shenzhen 518083
China

P.S. point-by-point responses to the reviewers' comments.

Reviewer #1: I thank the authors for addressing my comments in a satisfactory fashion. I have a couple of comments, more regarding choice of words.
Lines 111-112, and elsewhere in the manuscript: I am unsure if I would have chosen to discuss the N50 length of the scaffolds as "reaching a relatively high level among sequenced fish species". Do you mean length instead of level?

Answer: Thanks for your question and advice. You are right. We changed "level" to "length" on

line 110 of the revised manuscript.

While the manuscript discusses the manner of whole-genome duplication well, I found the wording in the answer a bit strange: "Black porgy is a diploid species, hence multiple copies of wnt4, vasa and JNK1 in the genome assembly may be resulted from the teleost-specific whole genome duplication." Most gene assemblers output a haploid assembly, based on a diploid genome, but sometimes both haplotypes are output. Do you mean that this has happened in this case? Or that the multiple copies have been retained since the whole-genome duplication in the ancestor to the teleosts?

Answer: Thanks for your good questions. Yes, you are right. The multiple copies may be retained since the whole-genome duplication in the ancestor to the teleost. Please find corresponding changes on lines 222-223 in the revised manuscript.

Reviewer #2: The revised version has been improved, but several of the reviewer comments have not been addressed appropriately. I therefore encourage the authors to take the following points into full consideration when revising the current version of the study:good questions
1. Comparison to other fish genome assemblies: I think that the assembly should only be compared to other teleost fishes. Elephant shark (cartilaginous fish) and coelacanth (lobe-finned fish), although being 'fish' in the broader sense, are no more justifiable to compare to than tetrapods.

Answer: Thanks for your comments and advice. We removed elephant shark and coelacanth in the revised manuscript. Please find more details on lines 111-114 in the revised manuscript.

2. BUSCO analysis: You mention that you used the BUSCOv2 actinopterygii dataset in the response to the reviewers, but this information needs to be included in the manuscript as well.

Answer: Thanks for your advice. According to the reviewer one’s opinions, we updated BUSCO v2 to v3, although the final results are similar. We hence added the updated information to the revised manuscript (on lines 118-121 and 148-150 of the revised manuscript).

3. Phylogenetic analysis:
3.1 The phylogenetic tree obtained with MrBayes should be included as a supplementary figure.

Answer: Thanks for your advice. However, please be informed that a Data Note paper do not provide space for any supplementary document. We rebuilt the phylogenetic tree using MrBayes (Version 3.2, with the GTR+gamma model), which is the same in topology as our Figure 2 in the main content.

3.2 In the response to the reviewers you mention that you removed the statement of a 'close relationship' of black porgy to fugu, but it is still in the manuscript (l. 174).

Answer: Sorry for the mistake. We removed it now in the new manuscript. Please see more details on lines 172-173 of the revised manuscript.

3.3 Furthermore, in the revised version it is newly stated that the phylogenetic tree 'suggests a lower neutral evolutionary rate than any other investigated teleost' l. 175). It is unclear how this statement is substantiated by the data presented. Is this referring to the branch lengths in the phylogenetic tree? Porgy and tilapia do not seem to be very different. The conclusion of a specifically slow rate of molecular evolution in black porgy has to be supported with a statistical tests such as relative rate and two-cluster tests.

Answer: Thanks for the nice comments and advice. Yes, you are right. We hence removed the conclusions due to the lack of sufficient evidence in the revised manuscript. Please see more details on lines 172-173.

4. Survey of sex related genes: This part still needs major improvement.
4.1. First of all, it still remains unresolved why there are multiple copies of many of these genes in the genome assembly. In the response to reviewer 1, it is stated that '… hence multiple copies of wnt4, vasa and JNK1 in the genome assembly may be resulted from the teleost-specific whole genome duplication.'; in response to reviewer 2: 'Several genes, such as Wnt4, vasa and JNK1, with multiple copies in the assembly may be copy number variants.' As mentioned in the previous reviews, this really needs to be addressed in more detail. For example, the table states that there are 15 copies of wnt4 in porgy. As the authors correctly state, there is wnt4a and wnt4b in vertebrates, as well as potential additional paralogs from the teleost genome duplication etc. All genes reported here need to be analyzed by phylogenetic methods to appropriately establish their orthology to other fishes and vertebrates. While I appreciate that the authors are in the process of cloning these individual copies, their sequences from the genome assembly need be submitted to NCBI and/or provided as supplements as part of the current article.

Answer: Thanks for your nice comments and advice. Sorry for the misleading statements. In fact, the multiple copies of wnt4, vasa and jnk1 in the genome assembly may be retained since the whole-genome duplication in the ancestor to the teleost. Please find more details of the corrections on lines 222-249 of the revised manuscript.
According to your advice, we employed all the predicted protein sequences to rebuild a new phylogenetic tree using PhyML, and we still observed that they were clustered with each corresponding homologue from other vertebrates (data not shown). Please find corresponding changes on lines 186-188 of the revised manuscript.
We also uploaded these data, including the ML tree and the predicted coding sequences and protein sequences of these genes, to the GigaDB.

4.2 Secondly, as mentioned in my previous report, the reasoning for the existence of a putative sex chromosome in black porgy needs to be supported beyond the fact that some of the sex-related genes are located on the same scaffold. Is the linkage of these specific genes in the black genome assembly unique or are these general blocks of conserved synteny across teleosts? The authors state: "our data demonstrate that the distribution of these 3 types of genes in the black porgy genome is similar to that in Chinese ricefield eel" but give no further explanation in which ways the porgy is similar to the rice eel. Again, if this refers to linkage of specific genes, it would need to be demonstrated that the linkage of these genes is unique to both porgy and ricefield eel. In their response to the reviewer, the authors further mention that the data are similar to arowana,

which has identified sex chromosomes, but give no further explanation in which ways porgy and arowana are similar to support the conclusion that a sex chromosome might be present in porgy. Without further details, such conclusion remains unsubstantiated and should be removed.

Answer: Thanks for your nice comments and advice. The linkage of these specific genes in the black genome assembly seems to be unique. However, we don’t have solid evidence to support existence of a sex chromosome in the porgy. We hence removed these conclusions in the revised manuscript (on lines 243-249). Meanwhile, more details about the similarity to ricefield eel were provided on lines 246-248 in the revised manuscript.

5. It would be better to mention the information on medaka dmrt1 after the statement on tongue sole dmrt1 (l. 198).

Answer: Thanks for your advice. It’s done on lines 200-202 of the revised manuscript.

6. Gene duplicates should be called paralogs, not 'isotypes'.

Answer: Sorry for our mistake. We corrected it on line 217 in the revised manuscript

7. Citations in the later part of the main text seem to out of order (starting at about citation 48 and following). Please double-check that the citations in the text and the bibliography correspond to each other.

Answer: Thanks for your careful checking. We corrected the citation orders in the revised manuscript.

8. Throughout the manuscript and tables, again, please follow the gene nomenclature conventions for teleost fish (see https://wiki.zfin.org/display/general/ZFIN+Zebrafish+Nomenclature+Guidelines).

Answer: Thanks for your comments. It’s done for all the gene names.
