# Supplementary material for: Draft genome of the protandrous Chinese black porgy, Acanthopagrus schlegelii
Source: Gigascience. 2018 Feb 26;7(4):giy012. doi: 10.1093/gigascience/giy012 (PMC5893958; doi:10.1093/gigascience/giy012)
Supplement: Response-to-Reviewer-Comments_Revision-2.pdf [file giy012_response-to-reviewer-comments_revision-2.pdf]

Author's Response To Reviewer Comments

Dear editor,
Thanks for your kind help. We also appreciate the instructive comments from the two reviewers. According to their suggestions, we made a careful revision, especially in addition of the actinopterygian BUSCO set (lines 120-121), definitions for the BUSCO values (lines 122-124), and a new column for the known vertebrate paralogs in the revised Table 3. Please see more details in the highlighted texts. Our point-by-point responses are also attached for your consideration.
Best regards,
Qiong Shi, PhD, Professor
BGI
Shenzhen 518083
China

Reviewer reports:
Reviewer #2:
The manuscript has further improved but some issues remain:
1. It is not explicitly mentioned in the text that the actinopterygian BUSCO set was used, so please revise:

Answer: Thanks for your good advice. We added the information on lines 120-121 of the revised manuscript.

l. 120: The final BUSCO score reached 89.1%, (C:89.1% [S:86.2%, D:2.9%], F:2.5%, M:8.4%, Actinopterygii gene set, n:4584).
l. 148: … and the final BUSCO score was up to 85.5% (C:85.5% [S:82.3%, D:3.2%], F:2.8%, M:11.7%, Actinopterygii gene set, n:4584).
It would also be helpful for the readers to define C, S, D, F, and M in the main text.

Answer: Thanks for the advice. Definitions of these values are added on lines 122-124 of the revised manuscript.

2. Table 3 unfortunately remains unrevised with regard to gene orthologies. Now that the authors have performed PhyML analyses and submitted results to GigaDB in support of the genes' actual orthologies (see response to reviewers; but a reference to the GigaDB data should also be put in the main text), they finally would need to revise the table and provide individual rows for known vertebrate and teleost paralogs based on these phylogenetic trees, such as (but not limited to): wnt4a, wnt4b; sox9a, sox9b, etc. Such change of table 3 in my opinion is necessary for publication, as it will also enable the authors to confirm whether or not some of the extra copies found in the black porgy genome are derived from the teleost fish genome duplication (as currently speculated in l. 222-223).
Btw. I don't think that oct4/pou5f1 does exist in teleosts, see publication PMID 23659605. The sequence reported is likely a different pou5f gene.

Answer: Thanks for your nice comments and advice. According to your suggestions, we revised the Table 3 with a new column for the known vertebrate paralogs.
You are right. pou5f1/oct4 has been extinct in teleosts, while it survives in tetrapods (Frankenberg and Renfree, 2013). Sorry for the mistake, we hence changed the oct4 to pou2 in the revised Table 3. As we know, teleost pou2 is an orthologue gene of the mammalian pou5f1/oct4, and it has been well characterized in zebrafish. Pou5f1 and pou2 are reported to have a conserved role in the regulation of pluripotency as well as germ cell maintenance and neural patterning in vertebrates (Frankenberg and Renfree, 2013; Khan et al, 2012).

Reference
Frankenberg S, Renfree M B. On the origin of POU5F1. BMC biology, 2013, 11(1): 56.
Khan A, Nakamoto A, Okamoto S, et al. Pou2, a class V POU-type transcription factor in zebrafish, regulates dorsoventral patterning and convergent extension movement at different blastula stages. Mechanisms of Development, 2012, 129(9): 219-235.
