# Supplementary material for: Draft genome of the protandrous Chinese black porgy, Acanthopagrus schlegelii
Source: Gigascience. 2018 Feb 26;7(4):giy012. doi: 10.1093/gigascience/giy012 (PMC5893958; doi:10.1093/gigascience/giy012)
Supplement: Response-to-Reviewer-Comments_Revision-3.pdf [file giy012_response-to-reviewer-comments_revision-3.pdf]

Author's Response To Reviewer Comments

Dear editor,
Thanks for your kind help. According to your suggestions, we made a careful revision. Addition of a new column for the copy number in other teleosts was performed in the revised Table 3. Please see more details in the highlighted texts.
Best regards,
Qiong Shi, PhD, Professor
BGI
Shenzhen 518083
China

Most of your revisions are very helpful, but in terms of the revised table 3, I'm afraid this is not quite what the reviewer had in mind.
The reviewer recommended: ".... to revise the table and provide individual rows for known vertebrate and teleost paralogs based on these phylogenetic trees, such as (but not limited to): wnt4a, wnt4b; sox9a, sox9b, etc. Such change of table 3 in my opinion is necessary for publication, as it will also enable the authors to confirm whether or not some of the extra copies found in the black porgy genome are derived from the teleost fish genome duplication (as currently speculated in l. 222-223). "
You have added an extra column (not rows) mentioning other species' orthologs/paralogs, but it's not easy to compare the number of extra copies /paralogs present in other species and in the black porgy, and thus to estimate whether or not some of the extra copies could be derived from the teleost genome duplication.
What the reviewer has rather in mind, as far as I understand, is to add individual *rows* for the other species' genes and indicate the number of copies present in other vertebrates for each of the genes, so that the numbers can be easily compared to the number of copies in the Porgy. (Actually, this may be easier to demonstrate in a separate table - that would be fine too, of course).
I am sorry for the back and forth regarding this point of the reviewer, but the referee has indicated that the comparison of orthologs/ paralogs is a quite important bit of information that should be displayed more clearly.

Answer: Thanks for your nice comments and advice. The previous column for known vertebrate paralogs was removed. However, it seems to be difficult to add rows for comparison of gene copy numbers. We hence revised the Table 3 with a new column for the copy number in other teleosts.
