# Supplementary material for: Draft genome of the protandrous Chinese black porgy, Acanthopagrus schlegelii
Source: Gigascience. 2018 Feb 26;7(4):giy012. doi: 10.1093/gigascience/giy012 (PMC5893958; doi:10.1093/gigascience/giy012)
Supplement: Reviewer-1-Report_Original-Submission -- Ole K Tørresen — 05 Sep 2017 Reviewed [file giy012_reviewer-1-report_original-submission.pdf]

Reviewer Report

Title: Draft Genome of the Protandrous Chinese Black Porgy, Acanthopagrus schlegelii

Version: Original Submission **Date:** 05 Sep 2017

Reviewer name: Ole K Tørresen

Reviewer Comments to Author:

General comments
Hermaphroditism is an interesting method of reproduction, where individuals change sex during their lifetime, or produce both eggs and sperm at the same time. While the genotypic basis for sex-determination in gonochoristic species (those that do not change sex) is being elucidated in more and more species, knowledge about the genotypic basis for sex change in hermaphrodite fishes is currently lacking.

The authors produce a genome assembly of the protandrous (male first) Chinese black porgy, Acanthopagrus schlegelii, annotates it, and do a survey of genes that are known to be involved in sex determination and differentiation. I greatly support publication of genome assemblies, and teleost genome assemblies in particular. The more data available for analysis for scientists, the better. However, I find the claims regarding the high quality of the genome assembly to be unsubstantiated. Quality is a difficult measure, but if it has to be used, then it has to be shown that something is better than something else, or at least equal to something of high quality. If the authors had shown that more complete genes are found with CEGMA and BUSCO than in other teleost genome assemblies, then I would have been convinced that this assembly is of high quality. They do not do that. As the genome assembly is presented in this manuscript, it does not warrant the modifier 'high quality'.

The authors also claim that black porgy has a compact genome, but do not shown much data that supports this. It has a similar genome size to many other teleosts, such as medaka, platyfish, Atlantic cod, Amazon molly, and substantially larger than species with known compact genomes such as fugu, tetraodon, stickleback and pipefishes. If the authors want to make a point of the supposed compactness of the black porgy genome, they need to make a much stronger case than this.

The authors do a survey of different genes associated with sex determination and differentiation in different species, but it is not clear to me why this is done. No further analysis than stating that these are present was done. Was the presence of these genes surprising? Then that should be stated properly. While the methods of sex determination and differential might not be conserved through teleosts, I do think we would expect the presence of most of these genes in most teleosts. In addition, the purpose for this analysis needs to be justified. Was it performed as a quality control of the genome assembly?

The specific programs, version and settings used for some of the analyses are not stated. Please address this because it is important for assessing the method and approach used, in addition to reproducibility.

I cannot recommend publication of this manuscript before the issues I highlight above and below are addressed.

Specific comments

Abstract:
Please do not use abbreviations such as "what's" in the abstract.

I am not sure that the black porgy is a "perfect" model for investigating sex change. It might very well be a good model.

Please change "...due to its protandrous hermaphrodite" to something like "...due to it being a protandrous hermaphrodite".

Please distinguish between a genome and a genome assembly. You can obtain a high-quality genome assembly, but not a high quality genome.

How do the authors assess "high-quality sequence reads"? High quality compared to what?

Please use lower case "scaffold" instead of capital letter in "...achieved Scaffold N50...".

Please use correct SI system units. Capital letter K, such as in Kb, is not a valid SI-system prefix (see https://en.wikipedia.org/wiki/Metric_prefix if unsure). kb is the correct abbreviation.

The last sentence of the findings part of the abstract should be rewritten to more correct and accurate English.

How do the authors assess the high quality of the genome assembly? High quality compared to what?

Data description:
How do the authors assess that the hybrid of Japanese seabream and Chinese black porgy is "excellent"? It can be "good" or "important", but I am unsure of how it can be "excellent".

I find the description of "improving the quality of sequencing reads" lacking. First, it is not possible to improve the quality of these. The sequencing machine sets the quality of these. I would have written something along the lines of "Before assembly of the sequencing reads, those with low-quality bases...". How was the removal of these reads done? Which program and which settings? Again, in what way is the resulting clean reads of "high-quality"? Is it necessary to specify the quality of these?

How can it be stated that the result will be a good quality genome assembly before running SOAPdenovo2? Better skip the word "quality" here, and just write "To obtain a genome assembly, we

ran SOAPdenovo2..."

In the assembly section, the authors suddenly mention "error corrected [sic]" reads, without mentioning how these were created. Please describe how the error-correction was performed, which program and which settings.

Why applying SSPACE on the scaffolds created by SOAPdenovo2? Does it create even longer and better scaffolds? How was the optimized parameters found?

Again, "scaffold" with lower case first letter, and not capital.

I like that CEGMA and BUSCO was used to validate the genome assembly. However, it is not specified whether or not the 92 % of CEGs found were complete or not. 92 % partial hits of CEGs are not especially impressive, but 92 % of complete hits are of course better.

In addition it is not specified which dataset was used for BUSCO. For BUSCOv3, the possible datasets (also called assessment sets) are Eukaryota, Metazoa, Vertebrata or Actinopterygii. However, while BUSCO version 3 is mentioned, it does not look like version 3 was used. From the number of genes found (n: 843), it looks like version 1 of BUSCO was run on the metazoan dataset, see Simão et al 2015. Since BUSCOv3 is mentioned, I would rather see the performance on the Actinopterygii dataset (found at http://busco.ezlab.org/) with BUSCOv3. Then http://www.biorxiv.org/content/early/2017/08/17/177485 should also be referred, where BUSCOv3 is described. For examples of performance of BUSCO on fish genome assembles, see for instance Jansen et al 2017 with BUSCOv3 run on the European eel genome assembly with the vertebrate dataset, finding 79.8 % complete genes (of 2586), or Liu et al 2017 where they find 81.4% complete and 9.1% partial vertebrate BUSCO genes (of 3023) using BUSCOv1 in the blunt snout bream genome assembly.

With the availability of the genome assembly, I downloaded it and ran CEGMA and BUSCOv2 using the actinopterygii dataset on it. While the results might vary from run to run or computer to computer, I find 86.3 % complete and 89.1 % partial genes with CEGMA. With BUSCOv2 I find 91.0 % complete genes (C:91.0%[S:88.1%,D:2.9%],F:1.1%,M:7.9%,n:4584). This is less than some of the genome assemblies I have looked at, but the authors should do their own comparisons.

After addressing these two issues (partial or complete CEGMA, correct dataset and version of BUSCO), then the authors can then assess whether or not the genome assembly is of high quality by comparing to other fish genome assemblies, for instance those two mentioned or other relevant ones.

It is good a de novo repeat library was created. However, direct comparisons of repeat content between different species are not straightforward. For instance, the difference of 23.70 % repeats in black porgy compared to 25.4 % in Atlantic cod and 25.2 % in stickleback is likely not significant. Different approaches would give different numbers. Also, at 739.6 Mbp Chinese black porgy might have a smaller genome than some other fishes, but it is not especially compact compared to fugu, tetraodon,

stickleback or the different pipefish and sea horse genomes.

The authors might confuse GENSCAN (identifying gene structures) with GENESCAN (analysis of sequencing data from ABI sequencer).

Use of transcriptome data can greatly improve the annotation of a genome assembly, but I am missing a better description of the sequencing data. Which program and which settings were used to remove low-quality bases, adapter and duplicated sequences? I am a bit surprised that TopHat1.2 was used. From the website (http://ccb.jhu.edu/software/tophat/index.shtml) it seems it was released in 2011. While researchers should keep up to date on which versions of relevant software is used, there might be specific reasons why some software used is out-of-date. However, 6 years old software is a bit of a stretch. Please use a newer version, and consider using HISAT2 instead.

The low number of annotated genes surprised me. Many fish species usually have about 20,000 genes or more annotated. These seem few and short, and I wonder if something went wrong during the annotation process. For instance, annotated fish genome assemblies in Ensembl of similar size (platyfish, medaka, and Amazon molly) have mean gene lengths around 13 - 17 kbp. However, the authors write that the average transcript length is 8.5 Kb (should be kb). In addition, the average number of exons per gene in those species is 11-15, while in black porgy it is 8.7. Too much of the assembly might have been masked for instance. I would like the authors to address this by doing a good validation of the annotation or redoing the annotation if it is faulty. BUSCO can also be used to validate the annotation, and I downloaded the predicted CDS and ran BUSCO on them. I found 65.4 % complete genes using the actinopterygii dataset (C:65.4%[S:63.0%,D:2.4%],F:4.6%,M:30.0%,n:4584). I am unsure how this compares to other species. From my experience, it is not unusual to find fewer genes in the predicted transcriptome than in the genome assembly.

I protest to the use of "proved" and "nice" when describing functional motifs and domains in the predicted genes/proteins. Rather write that "90 % of the predicted genes from the assembled genome contain at least one related functional…". The last sentence of this paragraph (starting with "These nice data...") can be skipped.

Phylogenetic analysis:
Why was the phylogenetic analysis done? Was the placement of black porgy among teleosts uncertain? I guess that Betancur-R et al 2017 might have come out too late to be used in the writing of this manuscript, but in that publication several other Acanthopagrus species are investigated and placed in the phylogeny. It is puzzling to perform an analysis without using it to draw any conclusions (expectations in gene content for instance).

Was the predicted transcript sequence for each protein used, or was each amino acid in the protein sequence from the MUSCLE alignment changed into the most common codon? If the latter, that would be strange and a lot of signal would be lost, if the first, that is a valid and proper approach. After looking at the script, it seems that the first is the approach used here. Please describe this section better.

I do not agree with the assertion that black porgy has a "close relationship" with fugu. These two species are separated by almost 100 million years of evolution (Betancur-R et al 2017) and are not close. The presumed closeness of these two species does not seem to affect any of the conclusions or speculations in this paper. Of the species included in the phylogeny, platyfish and medaka both have around 600-700 Mbp size genome assemblies, tilapia and zebrafish have a bit larger (1-1.4 Gbp), while stickleback and fugu have smaller assemblies around 400-450 Mbp. It seems strange to compare directly to fugu when the size of the genome of black porgy is 200-300 Mbp larger, and quite similar in size to medaka and platyfish. That is, it is not especially compact.

Analysis of three types of genes for sex change:
What is the purpose of this analysis? Were all of the genes downloaded from NCBI found in the genome assembly? How was homology asserted? Homology presumes common ancestry, which would need to be tested in some way. A highly confident BLAST hit could be enough for this, but the authors do not state what cut-offs were used.

The authors report that Wnt4, vasa and JNK1 are found in multiple copies in the genome assembly, and claim that these might be related to whole-genome duplication. Is it the teleost specific whole-genome duplication they think of, or do they propose additional genome duplication in the lineage leading to black porgy? If the latter, this should be investigated in more detail and properly argued. There are some genome duplications in teleosts, the lineage leading to carps and salmonids are two well-known examples.

This manuscript lacks several declaration sections. While it is stated in the text that "All animal experiments in this study were implemented in the light of the guidelines of the Animal Ethics Committee and ratified by the Institutional Review Board on Bioethics and Biosafety of BGI", it would be good to have this as a separate section also.

With 22 authors, and 6 of them contributing equally, I would also like to see an "author's contributions" section.

Level of Interest

Please indicate how interesting you found the manuscript: An article whose findings are important to those with closely related research interests

Quality of Written English

Please indicate the quality of language in the manuscript: Needs some language corrections before being published

Declaration of Competing Interests

Please complete a declaration of competing interests, considering the following questions:

- Have you in the past five years received reimbursements, fees, funding, or salary from an organisation that may in any way gain or lose financially from the publication of this manuscript, either now or in the future?
- Do you hold any stocks or shares in an organisation that may in any way gain or lose financially from the publication of this manuscript, either now or in the future?
- Do you hold or are you currently applying for any patents relating to the content of the manuscript?
- Have you received reimbursements, fees, funding, or salary from an organization that holds or has applied for patents relating to the content of the manuscript?
- Do you have any other financial competing interests?
- Do you have any non-financial competing interests in relation to this paper?

If you can answer no to all of the above, write 'I declare that I have no competing interests' below. If your reply is yes to any, please give details below.

I declare that I have no competing interests

I agree to the open peer review policy of the journal. I understand that my name will be included on my report to the authors and, if the manuscript is accepted for publication, my named report including any attachments I upload will be posted on the website along with the authors' responses. I agree for my report to be made available under an Open Access Creative Commons CC-BY license (http://creativecommons.org/licenses/by/4.0/). I understand that any comments which I do not wish to be included in my named report can be included as confidential comments to the editors, which will not be published.

I agree to the open peer review policy of the journal

To further support our reviewers, we have joined with Publons, where you can gain additional credit to further highlight your hard work (see: https://publons.com/journal/530/gigascience). On publication of this paper, your review will be automatically added to Publons, you can then choose whether or not to claim your Publons credit. I understand this statement.

Yes
