# Supplementary material for: Draft genome of the protandrous Chinese black porgy, Acanthopagrus schlegelii
Source: Gigascience. 2018 Feb 26;7(4):giy012. doi: 10.1093/gigascience/giy012 (PMC5893958; doi:10.1093/gigascience/giy012)
Supplement: Reviewer-1-Report_Revision-1 -- Ole K Tørresen — 07 Nov 2017 Reviewed [file giy012_reviewer-1-report_revision-1.pdf]

Reviewer Report

Title: Draft Genome of the Protandrous Chinese Black Porgy, Acanthopagrus schlegelii

Version: Revision 1 **Date:** 07 Nov 2017

Reviewer name: Ole K Tørresen

Reviewer Comments to Author:

I thank the authors for addressing my comments in a satisfactory fashion. I have a couple of comments, more regarding choice of words.

Lines 111-112, and elsewhere in the manuscript: I am unsure if I would have chosen to discuss the N50 length of the scaffolds as "reaching a relatively high level among sequenced fish species". Do you mean length instead of level?

While the manuscript discusses the manner of whole-genome duplication well, I found the wording in the answer a bit strange: "Black porgy is a diploid species, hence multiple copies of wnt4, vasa and JNK1 in the genome assembly may be resulted from the teleost-specific whole genome duplication." Most gene assemblers output a haploid assembly, based on a diploid genome, but sometimes both haplotypes are output. Do you mean that this has happened in this case? Or that the multiple copies have been retained since the whole-genome duplication in the ancestor to the teleosts?

Level of Interest

Please indicate how interesting you found the manuscript: An article whose findings are important to those with closely related research interests

Quality of Written English

Please indicate the quality of language in the manuscript: Acceptable

Declaration of Competing Interests

Please complete a declaration of competing interests, considering the following questions:

- Have you in the past five years received reimbursements, fees, funding, or salary from an organisation that may in any way gain or lose financially from the publication of this manuscript, either now or in the future?
- Do you hold any stocks or shares in an organisation that may in any way gain or lose financially from the publication of this manuscript, either now or in the future?

- Do you hold or are you currently applying for any patents relating to the content of the manuscript?
- Have you received reimbursements, fees, funding, or salary from an organization that holds or has applied for patents relating to the content of the manuscript?
- Do you have any other financial competing interests?
- Do you have any non-financial competing interests in relation to this paper?

If you can answer no to all of the above, write 'I declare that I have no competing interests' below. If your reply is yes to any, please give details below.

I declare that I have no competing interests

I agree to the open peer review policy of the journal. I understand that my name will be included on my report to the authors and, if the manuscript is accepted for publication, my named report including any attachments I upload will be posted on the website along with the authors' responses. I agree for my report to be made available under an Open Access Creative Commons CC-BY license (http://creativecommons.org/licenses/by/4.0/). I understand that any comments which I do not wish to be included in my named report can be included as confidential comments to the editors, which will not be published.

I agree to the open peer review policy of the journal

To further support our reviewers, we have joined with Publons, where you can gain additional credit to further highlight your hard work (see: https://publons.com/journal/530/gigascience). On publication of this paper, your review will be automatically added to Publons, you can then choose whether or not to claim your Publons credit. I understand this statement.

Yes
