# Supplementary material for: Draft genome of the protandrous Chinese black porgy, Acanthopagrus schlegelii
Source: Gigascience. 2018 Feb 26;7(4):giy012. doi: 10.1093/gigascience/giy012 (PMC5893958; doi:10.1093/gigascience/giy012)
Supplement: Reviewer-2-Report_Original-Submission -- Ingo Braasch — 11 Sep 2017 Reviewed [file giy012_reviewer-2-report_original-submission.pdf]

Reviewer Report

Title: Draft Genome of the Protandrous Chinese Black Porgy, Acanthopagrus schlegelii

Version: Original Submission **Date:** 11 Sep 2017

Reviewer name: Ingo Braasch

Reviewer Comments to Author:

The present Data Note reports the genome assembly of the black porgy, a teleost fish species of economic importance for the fisheries industry and of interest to understand ontogenetic sex change among vertebrates.

Standard methods have been used to generate a genome assembly of acceptable quality and the dataset has re-use potential. However, there are several issues that should be addressed before the manuscript is ready for publication. Please see a list below.
In addition, the level of writing needs to be improved with the help of a native speaker.

Specific points:

l. 61: confirm that "sea bread" is indeed a common name for this species

l. 103 and l. 109/110: How were the parameters for SOAPdenovo and SSPACE optimized?

l. 114-116: The other fish genome assemblies to which the black porgy assembly is compared appear random. Why did you choose these few assemblies for comparison?

l. 117-124: The CEGMA and BUSCO scores are good, but not outstanding. It is difficult to grasp the quality of the assembly from just providing these numbers and the authors could for example obtain CEGMA/BUSCO scores from the other fish genomes in l.114-116 for a more meaningful evaluation and comparison.

l. 129-133: I do not follow the conclusion that the black porgy has a particularly compact genome and is prominently sparse in repeat content. It is within the range of e.g. cod and stickleback, while zebrafish is a known outlier with an unusual high repeat content. The estimated porgy genome size also does not imply a specifically compact genome.

l. 138: How did you obtain the "homologous proteins of several reported fish"?

l. 142-144: At which level did you generate the mixture of transcriptomic data. Did you mix mRNA, cDNA, RNA-seq libraries or reads in silico?

Phylogenomic analysis:
- How did you chose the species to include in the analysis?
- Please provide bootstrap values for your tree?
- Do you obtain a similar tree with different, e.g. Bayesian, methods?
- How does the obtained tree compare to the known phylogenetic position of black porgy?

The grouping of porgy with pufferfish leads the authors to imply that there is shared genome compaction among them. Is this supported by knowledge about genome compaction in the pufferfish lineage? Again, I don't agree with the conclusions that the porgy genome is particularly compact.

l. 195-197: It would be important to mention that a duplicate of dmrt1 is the male sex determination gene in medaka.

l. 213: The term isoform should only be used for splice variants and not for gene duplicates such as sox9a and sox9b, which were generated during the teleost genome duplication.

Please follow the established gene nomenclature for fish genes.

Table 3: Gene name should be oct4, not 4-Oct.

Analysis of candidate genes for sex determination and sex change:
It is curious that there are a number of genes in the list with multiple copies in the assembly. Please provide more details on these cases. Are these extra copies different alleles, copy number variants or potential redundancy artifacts of the genome assembly? How do these extra copies relate to the teleost whole genome duplication and other known duplications of these genes in fish and vertebrates in general?
Finally, it would be good to provide the predicted sequences of the candidate genes as a separate sequence file.

l. 230-238: Is the similarity of genetic linkage of some of the sex-related genes in porgy and rice eel a specific characteristic of these two lineages or are they rather an indication of conserved synteny among teleost fishes in general?
Please explain better how genes distributed among several scaffolds (1, 2, 3, 11, 15) are a potential indication for a sex chromosome. Also, would you expect a species with ontogenetic sex change to have a sex chromosome at all? Please explain.

Level of Interest

Please indicate how interesting you found the manuscript: An article whose findings are important to those with closely related research interests

Quality of Written English

Please indicate the quality of language in the manuscript: Needs some language corrections before being published

Declaration of Competing Interests

Please complete a declaration of competing interests, considering the following questions:

- Have you in the past five years received reimbursements, fees, funding, or salary from an organisation that may in any way gain or lose financially from the publication of this manuscript, either now or in the future?
- Do you hold any stocks or shares in an organisation that may in any way gain or lose financially from the publication of this manuscript, either now or in the future?
- Do you hold or are you currently applying for any patents relating to the content of the manuscript?
- Have you received reimbursements, fees, funding, or salary from an organization that holds or has applied for patents relating to the content of the manuscript?
- Do you have any other financial competing interests?
- Do you have any non-financial competing interests in relation to this paper?

If you can answer no to all of the above, write 'I declare that I have no competing interests' below. If your reply is yes to any, please give details below.

I declare that I have no competing interests.

I agree to the open peer review policy of the journal. I understand that my name will be included on my report to the authors and, if the manuscript is accepted for publication, my named report including any attachments I upload will be posted on the website along with the authors' responses. I agree for my report to be made available under an Open Access Creative Commons CC-BY license (http://creativecommons.org/licenses/by/4.0/). I understand that any comments which I do not wish to be included in my named report can be included as confidential comments to the editors, which will not be published.

I agree to the open peer review policy of the journal

To further support our reviewers, we have joined with Publons, where you can gain additional credit to further highlight your hard work (see: https://publons.com/journal/530/gigascience). On publication of

this paper, your review will be automatically added to Publons, you can then choose whether or not to claim your Publons credit. I understand this statement.

Yes
