# Supplementary material for: Draft genome of the protandrous Chinese black porgy, Acanthopagrus schlegelii
Source: Gigascience. 2018 Feb 26;7(4):giy012. doi: 10.1093/gigascience/giy012 (PMC5893958; doi:10.1093/gigascience/giy012)
Supplement: Reviewer-2-Report_Revision-1 -- Ingo Braasch — 07 Nov 2017 Reviewed [file giy012_reviewer-2-report_revision-1.pdf]

Reviewer Report

Title: Draft Genome of the Protandrous Chinese Black Porgy, Acanthopagrus schlegelii

Version: Revision 1 **Date:** 07 Nov 2017

Reviewer name: Ingo Braasch

Reviewer Comments to Author:

The revised version has been improved, but several of the reviewer comments have not been addressed appropriately. I therefore encourage the authors to take the following points into full consideration when revising the current version of the study:

1. Comparison to other fish genome assemblies: I think that the assembly should only be compared to other teleost fishes. Elephant shark (cartilaginous fish) and coelacanth (lobe-finned fish), although being 'fish' in the broader sense, are no more justifiable to compare to than tetrapods.

2. BUSCO analysis: You mention that you used the BUSCOv2 actinopterygii dataset in the response to the reviewers, but this information needs to be included in the manuscript as well.

3. Phylogenetic analysis:

3.1 The phylogenetic tree obtained with MrBayes should be included as a supplementary figure.

3.2 In the response to the reviewers you mention that you removed the statement of a 'close relationship' of black porgy to fugu, but it is still in the manuscript (l. 174).

3.3 Furthermore, in the revised version it is newly stated that the phylogenetic tree 'suggests a lower neutral evolutionary rate than any other investigated teleost' l. 175). It is unclear how this statement is substantiated by the data presented. Is this referring to the branch lengths in the phylogenetic tree? Porgy and tilapia do not seem to be very different. The conclusion of a specifically slow rate of molecular evolution in black porgy has to be supported with a statistical tests such as relative rate and two-cluster tests.

4. Survey of sex related genes: This part still needs major improvement.

4.1. First of all, it still remains unresolved why there are multiple copies of many of these genes in the genome assembly. In the response to reviewer 1, it is stated that '... hence multiple copies of wnt4, vasa and JNK1 in the genome assembly may be resulted from the teleost-specific whole genome duplication.'; in response to reviewer 2: 'Several genes, such as Wnt4, vasa and JNK1, with multiple copies in the assembly may be copy number variants.' As mentioned in the previous reviews, this really needs to be addressed in more detail. For example, the table states that there are 15 copies of wnt4 in

porgy. As the authors correctly state, there is wnt4a and wnt4b in vertebrates, as well as potential additional paralogs from the teleost genome duplication etc. All genes reported here need to be analyzed by phylogenetic methods to appropriately establish their orthology to other fishes and vertebrates. While I appreciate that the authors are in the process of cloning these individual copies, their sequences from the genome assembly need be submitted to NCBI and/or provided as supplements as part of the current article.

4.2 Secondly, as mentioned in my previous report, the reasoning for the existence of a putative sex chromosome in black porgy needs to be supported beyond the fact that some of the sex-related genes are located on the same scaffold. Is the linkage of these specific genes in the black genome assembly unique or are these general blocks of conserved synteny across teleosts? The authors state: "our data demonstrate that the distribution of these 3 types of genes in the black porgy genome is similar to that in Chinese ricefield eel" but give no further explanation in which ways the porgy is similar to the rice eel. Again, if this refers to linkage of specific genes, it would need to be demonstrated that the linkage of these genes is unique to both porgy and ricefield eel. In their response to the reviewer, the authors further mention that the data are similar to arowana, which has identified sex chromosomes, but give no further explanation in which ways porgy and arowana are similar to support the conclusion that a sex chromosome might be present in porgy. Without further details, such conclusion remains unsubstantiated and should be removed.

5. It would be better to mention the information on medaka dmrt1 after the statement on tongue sole dmrt1 (l. 198).

6. Gene duplicates should be called paralogs, not 'isotypes'.

7. Citations in the later part of the main text seem to out of order (starting at about citation 48 and following). Please double-check that the citations in the text and the bibliography correspond to each other.

8. Throughout the manuscript and tables, again, please follow the gene nomenclature conventions for teleost fish (see https://wiki.zfin.org/display/general/ZFIN+Zebrafish+Nomenclature+Guidelines).

Level of Interest

Please indicate how interesting you found the manuscript: An article whose findings are important to those with closely related research interests

Quality of Written English

Please indicate the quality of language in the manuscript: Needs some language corrections before being published

Declaration of Competing Interests

Please complete a declaration of competing interests, considering the following questions:

- Have you in the past five years received reimbursements, fees, funding, or salary from an organisation that may in any way gain or lose financially from the publication of this manuscript, either now or in the future?
- Do you hold any stocks or shares in an organisation that may in any way gain or lose financially from the publication of this manuscript, either now or in the future?
- Do you hold or are you currently applying for any patents relating to the content of the manuscript?
- Have you received reimbursements, fees, funding, or salary from an organization that holds or has applied for patents relating to the content of the manuscript?
- Do you have any other financial competing interests?
- Do you have any non-financial competing interests in relation to this paper?

If you can answer no to all of the above, write 'I declare that I have no competing interests' below. If your reply is yes to any, please give details below.

I declare that I have no competing interests.

I agree to the open peer review policy of the journal. I understand that my name will be included on my report to the authors and, if the manuscript is accepted for publication, my named report including any attachments I upload will be posted on the website along with the authors' responses. I agree for my report to be made available under an Open Access Creative Commons CC-BY license (http://creativecommons.org/licenses/by/4.0/). I understand that any comments which I do not wish to be included in my named report can be included as confidential comments to the editors, which will not be published.

I agree to the open peer review policy of the journal

To further support our reviewers, we have joined with Publons, where you can gain additional credit to further highlight your hard work (see: https://publons.com/journal/530/gigascience). On publication of this paper, your review will be automatically added to Publons, you can then choose whether or not to claim your Publons credit. I understand this statement.

Yes
