# Supplementary material for: Draft genome of the protandrous Chinese black porgy, Acanthopagrus schlegelii
Source: Gigascience. 2018 Feb 26;7(4):giy012. doi: 10.1093/gigascience/giy012 (PMC5893958; doi:10.1093/gigascience/giy012)
Supplement: Reviewer-2-Report_Revision-2 -- Ingo Braasch — 08 Dec 2017 Reviewed [file giy012_reviewer-2-report_revision-2.pdf]

Reviewer Report

Title: Draft Genome of the Protandrous Chinese Black Porgy, Acanthopagrus schlegelii

Version: Revision 2 **Date:** 08 Dec 2017

Reviewer name: Ingo Braasch

Reviewer Comments to Author:

The manuscript has further improved but some issues remain:

1. It is not explicitly mentioned in the text that the actinopterygian BUSCO set was used, so please revise:

l. 120: The final BUSCO score reached 89.1%, (C:89.1% [S:86.2%, D:2.9%], F:2.5%, M:8.4%, Actinopterygii gene set, n:4584).

l. 148: ... and the final BUSCO score was up to 85.5% (C:85.5% [S:82.3%, D:3.2%], F:2.8%, M:11.7%, Actinopterygii gene set, n:4584).

It would also be helpful for the readers to define C, S, D, F, and M in the main text.

2. Table 3 unfortunately remains unrevised with regard to gene orthologies. Now that the authors have performed PhyML analyses and submitted results to GigaDB in support of the genes' actual orthologies (see response to reviewers; but a reference to the GigaDB data should also be put in the main text), they finally would need to revise the table and provide individual rows for known vertebrate and teleost paralogs based on these phylogenetic trees, such as (but not limited to): wnt4a, wnt4b; sox9a, sox9b, etc. Such change of table 3 in my opinion is necessary for publication, as it will also enable the authors to confirm whether or not some of the extra copies found in the black porgy genome are derived from the teleost fish genome duplication (as currently speculated in l. 222-223).
Btw. I don't think that oct4/pou5f1 does exist in teleosts, see publication PMID 23659605. The sequence reported is likely a different pou5f gene.

Level of Interest

Please indicate how interesting you found the manuscript: An article whose findings are important to those with closely related research interests

Quality of Written English

Please indicate the quality of language in the manuscript: Needs some language corrections before being published

Declaration of Competing Interests

Please complete a declaration of competing interests, considering the following questions:

- Have you in the past five years received reimbursements, fees, funding, or salary from an organisation that may in any way gain or lose financially from the publication of this manuscript, either now or in the future?
- Do you hold any stocks or shares in an organisation that may in any way gain or lose financially from the publication of this manuscript, either now or in the future?
- Do you hold or are you currently applying for any patents relating to the content of the manuscript?
- Have you received reimbursements, fees, funding, or salary from an organization that holds or has applied for patents relating to the content of the manuscript?
- Do you have any other financial competing interests?
- Do you have any non-financial competing interests in relation to this paper?

If you can answer no to all of the above, write 'I declare that I have no competing interests' below. If your reply is yes to any, please give details below.

NA

I agree to the open peer review policy of the journal. I understand that my name will be included on my report to the authors and, if the manuscript is accepted for publication, my named report including any attachments I upload will be posted on the website along with the authors' responses. I agree for my report to be made available under an Open Access Creative Commons CC-BY license (http://creativecommons.org/licenses/by/4.0/). I understand that any comments which I do not wish to be included in my named report can be included as confidential comments to the editors, which will not be published.

I agree to the open peer review policy of the journal

To further support our reviewers, we have joined with Publons, where you can gain additional credit to further highlight your hard work (see: https://publons.com/journal/530/gigascience). On publication of this paper, your review will be automatically added to Publons, you can then choose whether or not to claim your Publons credit. I understand this statement.

Yes
